# Hsa-miR-99b/let-7e/miR-125a Cluster Regulates Pathogen Recognition Receptor-Stimulated Suppressive Antigen-Presenting Cells

**DOI:** 10.3389/fimmu.2018.01224

**Published:** 2018-06-18

**Authors:** Dagmar Hildebrand, Mariel-Esther Eberle, Sabine Marie Wölfle, Franziska Egler, Delal Sahin, Aline Sähr, Konrad A. Bode, Klaus Heeg

**Affiliations:** ^1^Medical Microbiology and Hygiene, Centre for Infectious Diseases, University Hospital Heidelberg, Heidelberg, Germany; ^2^German Center for Infection Research (DZIF), Brunswick, Germany

**Keywords:** suppressive antigen-presenting cell, miRNA, hsa-miR-99b/let-7e/miR-125a cluster, signal transducer and activator of transcription 3, programmed death-ligand 1, indolamin-2,3-dioxygenase

## Abstract

Antigen-presenting cells (APCs) regulate the balance of our immune response toward microbes. Whereas immunogenic APCs boost inflammation and activate lymphocytes, the highly plastic cells can switch into a tolerogenic/suppressive phenotype that dampens and resolves the response. Thereby the initially mediated inflammation seems to prime the switch of APCs while the strength of activation determines the grade of the suppressive phenotype. Recently, we showed that pathogen recognition receptor-mediated pro-inflammatory cytokines reprogram differentiating human blood monocytes *in vitro* toward an immunosuppressive phenotype through prolonged activation of signal transducer and activator of transcription (STAT) 3. The TLR7/8 ligand R848 (Resiquimod) triggers the high release of cytokines from GM-CSF/IL-4-treated monocytes. These cytokines subsequently upregulate T cell suppressive factors, such as programmed death-ligand 1 (PD-L1) and indolamin-2,3-dioxygenase (IDO) through cytokine receptor-mediated STAT3 activation. Here, we reveal an essential role for the microRNA (miR, miRNA) hsa-miR-99b/let-7e/miR-125a cluster in stabilizing the suppressive phenotype of R848-stimulated APCs on different levels. On the one hand, the miR cluster boosts R848-stimulated cytokine production through regulation of MAPkinase inhibitor Tribbles pseudokinase 2, thereby enhancing cytokine-stimulated activation of STAT3. One the other hand, the STAT3 inhibitor suppressor of cytokine signaling-1 is targeted by the miR cluster, stabilizing the STAT3-induced expression of immunosuppressive factors PD-L1 and IDO. Finally, hsa-miR-99b/let-7e/miR-125a cluster regulates generation of the suppressive tryptophan (Trp) metabolite kynurenine by targeting the tryptophanyl-tRNA synthetase WARS, the direct competitor of IDO in terms of availability of Trp. In summary, our results reveal the hsa-miR-99b/let-7e/miR-125a cluster as an important player in the concerted combination of mechanisms that stabilizes STAT3 activity and thus regulate R848-stimulated suppressive APCs.

## Introduction

Antigen-presenting cells (APCs) such as monocytes, macrophages, and dendritic cells (DCs) are highly plastic in nature and central players in the immune response. Depending on the environment, APCs can facilitate immunity or favor tolerance *via* promoting anergy and deletion of T cells or regulatory T cells (Tregs) (1–3) Examples of APCs that prevent an immune response can be found in special niches of our body, such as the gastrointestinal tract and the liver, where they impede an overreaction against commensal bacteria, but at the same time possess the potential to switch to an immunogenic population after infection (4–7). Furthermore, suppressive APCs may downregulate already induced immune reactions. For example, macrophages that are attracted to the side of infection and eliminate dead neutrophils switch into a phenotype that facilitates killing of pathogens and promotes wound healing, yet inhibits effector T cells (8, 9). Thus, the initiated immune response primes its own resolution.

Although the range of tolerogenic/suppressive APCs is broad, some of the conducted immune inhibitory reactions appear to be universal and thus looked upon checkpoint mechanisms. Besides anti-inflammatory cytokine effects, inhibitory checkpoint mechanisms include programmed death-ligand 1 (PD-L1) binding to programmed cell death 1 (PD-1) on T cells (10–12) and modulation through indolamin-2,3-dioxygenase (IDO)-catalyzed tryptophan metabolites (13–15).

To investigate regulation and function of inhibitory mechanisms, we established an *in vitro* system to generate suppressive APCs (16). While stimulation of blood-derived monocytes with GM-CSF/IL-4 typically led to differentiation of immature DCs (iDCs), additional stimulation with the TLR7/8- and NLR-activator R848 (Resiquimod) at the very beginning provoke the differentiation of suppressive APC. Phenotypically, these APCs suppress CD3/28- and DC-stimulated T cell activation in MLRs and prime Tregs. Importantly, the suppressive phenotype is highly dependent on activation of signal transducer and activator of transcription (STAT) 3. We have shown that PRR-mediated pro-inflammatory cytokines activate the key transcription factor STAT3 and the prolonged activity ultimately induces a shift in gene expression and induction of several T cell suppressive factors (16, 17). STAT3 is known to be a key transcription factor in immune suppression. Thus, different regulatory mechanisms supervise its activity on the post-transcriptional level. Recently, the role of miRNAs as modulators of STAT3 activity have been increasingly discussed (18). miRNAs are evolutionarily conserved, small (19–23 nucleotides) non-coding RNAs. They regulate gene expression through binding predominantly but not exclusively within the 3′untranslated region of protein-coding mRNAs. As miRNAs do not require a perfect base pair match with their target sequences, one miRNA can block translation of various mRNAs and one mRNA can be targeted by different miRNAs (19–22). miRNAs are known to have a major role in cell differentiation and fixation of cellular phenotypes (23, 24). Nevertheless, their impact on terminal differentiation, such as differentiation of blood monocytes toward activating DCs or suppressing myeloid cells, is poorly understood.

In this study, we set out to clarify whether miRNAs contribute to stabilizing the activity of STAT3, which in turn promotes the suppressive APC phenotype. Here, we show that the hsa-miR-99b/let-7e/miR-125a cluster, which was shown to be directly induced by PAMP-stimulated NF-κB signaling (25) and cytokine receptor-activated STAT3 transcription factor (18) is highly upregulated in R848-stimulated suppressive APCs. The hsa-miR-99b/let-7e/miR-125a cluster can be found on chromosome 19 in the q13.41 region and is mostly due to miR-125a known for its regulatory role is hematopoietic stem cell development and apoptosis (26). We show that the hsa-miR-99b/let-7e/miR-125a cluster stabilizes STAT3 activation by targeting and downregulating a suppressor of MAPkinase signaling termed Tribbles pseudokinase 2 (TRIB2) (27). Restorage of TRIB2 through inhibition of the hsa-miR-99b/let-7e/miR-125a cluster by specific antagomiRs (amiRs) resulted in a reduced Mapkinase p42/44 and p38 activation and signaling leading to diminished cytokine production. Furthermore, the miR cluster stabilizes activation of STAT3 by targeting the respective feedback inhibitor of cytokine receptor signaling suppressor of cytokine signaling-1 (SOCS1) (28). Both mechanisms correlated with a decreased STAT3 activation and eventually a reduced induction of the STAT3-dependent target genes PD-L1 and IDO that mediate inhibition of T cell proliferation. Moreover, the miR cluster was found to target WARS, a direct IDO competitor for Trp, and thereby led to enhanced generation of the immunosuppressive Trp metabolite kynurenine. Collectively, our data demonstrate that conservation of the suppressive phenotype can be brought about by multiple pathways and mechanisms, the hsa-miR-99b/let-7e/miR-125a cluster playing an important role in this process.

## Materials and Methods

### Reagents

Recombinant cytokines were purchased from PeproTech (Hamburg, Germany). R848 was bought from ALEXIS (Lausen, Switzerland). Antibodies for FACS analyses were acquired from BD Biosciences (Heidelberg, Germany). All Western blot antibodies were purchased from Cell Signaling Technology (Danvers, MA, USA).

### Isolation of Primary Human Monocytes

PBMCs were isolated from fresh blood or buffy coat from healthy donors by density-gradient centrifugation (Biocoll separating solution, 1.077 g/ml, Biochrom AG, Berlin, Germany). After washing with PBS, CD14^+^ cells were magnetically labeled with beads (Miltenyi Biotec) and selected *via* the autoMACS separator (autoMACS, program: possel, Miltenyi Biotec, Bergisch Gladbach, Germany) twice. Untouched CD4^+^ T cells were isolated by the CD4^+^ T Cell Isolation Kit (Miltenyi Biotec) and autoMACS program: depletes. Cells were cultured in RPMI 1640 (Sigma-Aldrich, Taufkirchen, Germany) supplemented with 100 IU/ml of penicillin, 100 µg/ml streptomycin, and 10% heat-inactivated fetal calf serum (PromoCell, Heidelberg, Germany) at 37°C in a humidified atmosphere in the presence of 5% CO_2_.

### Stimulation

Monocyte cultures (1 × 10^6^ cells/ml) were supplemented with rhGM-CSF and rhIL-4 at final concentrations of 10 and 20 ng/ml, respectively. To obtain the R848-stimulated APCs, 5 µg/ml R848 were provided in addition. Cells were then cultured at 37°C, 95% humidity and 5% CO_2_ for another 24 or 72 h.

### miRNA Array

Analysis of miRNA profile was performed as follows. R848-stimulated APCs and iDCs were generated as described above (see Isolation of Primary Human Monocytes and Stimulation). Cells were seeded in 24-well format in three replicates. After 72 h in culture, 4 × 10^6^ cells were harvested, lysed, and total RNA was isolated using the High Pure RNA isolation kit (Roche, Mannheim, Germany). Thereupon, at least 500 ng of purified RNA was applied to miRNA analysis. Concentration and purity of RNA was determined *via* Gel analysis using the Nano 2100 bioanalyzer, version 2.6 (DE54700489, Agilent Technologies, Berlin, Germany). miRNA Expression profiling was performed by DKFZ-Genomics and Proteomics Core Facility (TP3, DKFZ, Heidelberg, Germany). In brief, fluorescently labeled miRNAs were prepared according to the respective protocol from Agilent (“miRNA Complete Labeling and Hyb Kit,” Agilent Technologies, Berlin, Germany). Afterward, 200 ng of labeled miRNA samples was hybridized for at least 20 h at 55°C on Agilent human miRNA Microarray Release 16.0, 8 × 60k. Expression Microarrays were scanned using Agilent Scanner G2505C. The scanned images were analyzed with Feature Extraction Software (Agilent Technologies) using default settings.

The miRNA array data discussed in this publication have been deposited in NSBIs Gene Expression Omnibus [GEO; (29)] and are accessible through GEO series accession number GSE 114390 (https://www.ncbi.nlm.nih.gov/geo/query/acc.cgi?acc=GSE114390).

### MicroRNA Knockdown and Stimulation of Monocytes

Knockdown of miRNAs was obtained by transfection with oligodeoxynucleotide (ODN)/amiR constructs added directly after seeding at final concentrations of 100 nM. Specificity was conferred by a fully 2′-O-methylated oligoribonucleotide (ORN) sequence complementary to the miRNA of interest. A poly CT phosphorothioate-modified ODN sequence separated from the 3′-end of the ORN by a carbon chain linker served to provide both additional stability and facilitate uptake. amiR constructs were custom synthesized by IBA (Göttingen, Germany) with sequences as follows: polyCT 125a (5′-CTC TCT CTC TCT CTC TCT CTC T-2 × (CH2)3-3′-AGG GAC UCU GGG AAA UUG GAC ACU-5′), polyCT 99b (5′-CTC TCT CTC TCT CTC TCT CTC T-2 × (CH2)3-3′-GUG GGC AUC UUG GCU GGA ACG-5′), polyCT let-7e (5′-CTC TCT CTC TCT CTC TCT CTC T-2 × (CH2)3-3′-ACUCCAUCCUCCAACAUAUCA-5′), polyCT cel (5′-CTC TCT CTC TCT CTC TCT CTC T-2 × (CH2)3-3′-UCA CAA CCU CCU AGA AAG AGUA-5′). After overnight culture ± ODN/amiR constructs, cells were differentiated into iDCs or R848-stim.APCs.

### Carboxyfluorescein Succinimidyl Ester (CFSE)-Proliferation Assay

Monocytes were treated as reported. After 3 days, allogeneic, CFSE-labeled, CD3/CD28-activated CD4^+^ T cells were added in a ratio of 1:2. For CFSE-labeling, T cells were stained for 10 min at r.t. in 0.3 mM CFSE/PBS (Molecular Probes, San Diego, CA, USA) and washed afterward with PBS containing 10% FCS. Three to five days after start of coculture, cell divisions were analyzed by determining the FITC signal using a FACScanto.

### Flow Cytometry

Three to five days after stimulation, monocytes were analyzed for surface markers with antibody staining: anti-PD-L1-PE (BD Biosciences, Heidelberg, Germany) and anti-HLA-DR-FITC (eBioscience, Frankfurt/Main, Germany). Mean fluorescence was recorded using the FACS DIVA V 4.12 software on a FACS Canto (BD Biosciences). Overlays were performed with the Weasel v2.5 software (WEHI, Melbourne, VIC, Australia). FOXP3 expression in T cells was assessed using an anti-human FoxP3 Staining Kit (e-Biosciences, San Diego, CA, USA). Previous to intracellular staining, cells were stained with anti-CD25-APC antibody (BD Biosciences, Heidelberg, Germany).

### Quantitative Reverse Transcription PCR (qRT-PCR)

Total RNA was extracted from 2 × 10^6^ cultured primary human monocytes using the high pure RNA isolation kit (Roche, Mannheim, Germany) according to the manufacturer’s protocol. RNA preparations for miRNA expression analyses were carried out using the Ambion miRVana kit (Thermo Fischer Scientific, Karlsruhe, Germany) according to the manufacturer’s instructions. In brief, 2 × 10^6^ cells were harvested by centrifugation, lysed and subjected to organic extraction with acid phenol:chloroform, followed by solid-phase extraction based on glass-fiber filters for efficient enrichment of small RNA species. RNA preparations enriched or not for small RNA species were then quantified by spectrophotometry (NanoDrop ND-100 Spectrometer, Peqlab, Erlangen, Germany) and equal amounts were reverse transcribed using the miScript II RT (Qiagen, Hilden, Germany) and Reverse Aid First Strand cDNA synthesis kits (Thermo Fischer Scientific, Karlsruhe, Germany), respectively. Obtained cDNA was used for quantitative PCR utilizing the “SYBR green ROX mix” (Thermo Fischer Scientific, Karlsruhe, Germany) and the following sequence-specific primers with regard to expression of protein-coding genes: actin fwd 5′-AGA GCT ACG AGC TGC CTG AC-3′, actin rev 5′-AGC ACT GTG TTG GCG TAC AG-3′, SOCS1 fwd 5′-TCC CCC TCA ACC CCG T-3′, SOCS1 rev 5′-CAT CCG CTC CCT CCA ACC-3′, TRIB2 fwd 5′-CAA GCT GCG GAA ATT CAT CT-3′, TRIB2 rev 5′-GTA GCT GCC ACT GGT GTT CA-3′, WARS fwd 5′-CGC AGA GGC ATC TTC TTC TC-3′, WARS rev 5′-TGA CCA AGG GCA CGT TAA AT-3′. miRNA expression was determined in a similar manner using the miScript SYBR green PCR kit (Qiagen, Hilden, Germany) and the corresponding primers: has-miR-125a 5′-UCC CUG AGA CCC UUU AAC CUG UGA-3′, has-miR-99b 5′-CAC CCG UAG AAC CGA CCU UGC G-3′, has-let-7e 5′-UGA GGU AGG AGG UUG UAU AGU U-3′.

### Enzyme-Linked Immunosorbent Assay (ELISA)

Commercially available ELISA kits (BD OptEIA ELISA Set; BD Biosciences Pharmingen, Heidelberg, Germany) were utilized for IL-6 and IL-10 detection in cell-free supernatants according to the manufacturer’s instructions. Absorbance measurements were performed on a SUNRISE Absorbance reader (Tecan, Salzburg, Austria) and analyzed with Magellan software.

### Western Blotting

2 × 10^6^ cells were harvested by 10 min centrifugation at 1,300 × *g* and 4°C, after which they were washed with 1 ml of PBS. Lysis was then performed in 50 µl of RIPA buffer (50 mM Tris–HCl, pH 7.4; 1% Igepal; 0.25% sodium deoxycholate; 150 mM NaCl; 1 mM EDTA; 1 mM PMSF; 1 mg/ml each aprotinin, leupeptin, and pepstatin; 1 mM Na3VO4; and 1 mM NaF). Samples were vortexed and incubated 30 min on ice. Lysates were then cleared *via* centrifugation at 14,000 × *g* for 20 min and equal amounts were used for separation by SDS-PAGE (12.5%). After semi-dry transfer onto nitrocellulose membranes (Whatman Protran nitrocellulose membrane; neoLab, Heidelberg, Germany), the latter were blocked with 5% (w/v) BSA in TBS/0.1% (v/v) Tween-20 for 2 h at RT. Probing was performed with the antibodies indicated and detection was based on enhanced chemiluminescence (ECL; Perkin Elmer, Groningen, Netherlands). For analyzing un-phosphorylated and phosphorylated forms of the same protein, stripping of membranes was performed in stripping buffer [2% (w/v) SDS, 62.5 mM Tris–HCl pH 6.8, 0.7% (v/v) β-mercaptoethanol] for 30 min at 50°C. Extensive washing with TBS/0.1% (v/v) Tween-20 was followed by another blocking step and re-probing with the corresponding antibodies.

### Statistical Analysis

The comparison of two data groups were analyzed by Mann–Whitney *U* test.

### Ethical Statement

This study was carried out in accordance with the recommendations of the ethics committee of the Medizinische Fakultät Heidelberg with written informed consent from all subjects. All subjects gave written informed consent in accordance with the Declaration of Helsinki. The study (taking of blood samples from healthy donors and treatment of blood leukocytes with microbial stimuli) was reviewed and approved by the ethics committee of Medizinische Fakultät Heidelberg.

## Results

**Figure 1:**
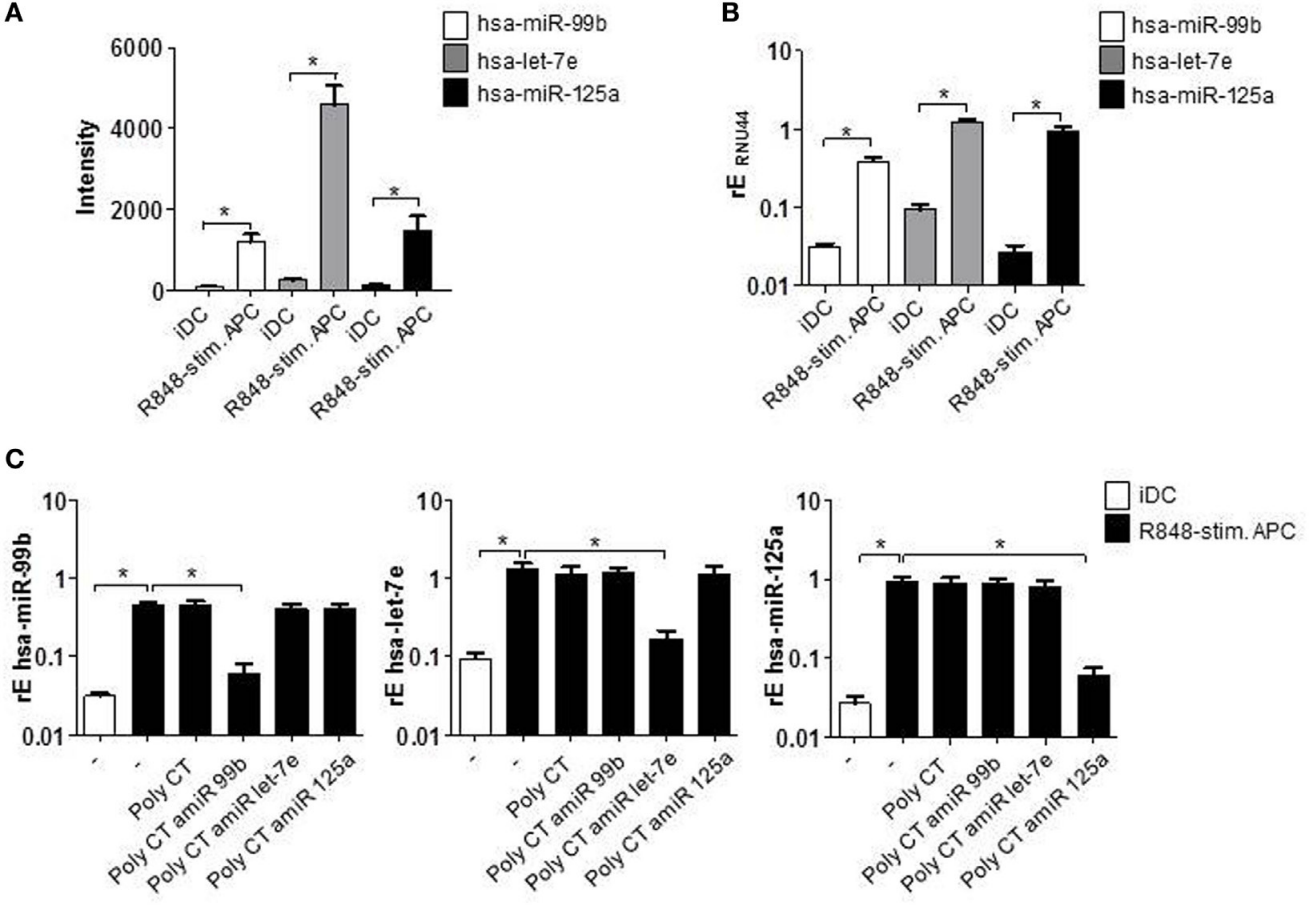
CD14^+^ monocytes were isolated from blood of healthy donors and stimulated with GM-CSF and IL-4 or GM-CSF, IL-4, and R848 to generate immature DCs (iDCs) and R848-stimulated suppressive APCs (R848-stim.APC), respectively. **(A)** miRNA abundance. iDCs and R848-stim.APCs were analyzed by miRNA array. Shown is the intensity (abundance of probe-target hybridization) of hsa-miR-99b, hsa-let-7e, and hsa-miR-125a. **(B)** RNA was isolated and cDNA produced. Induction of miRNAs was analyzed by quantitative (q) RT PCR using sequence-specific primer (RNU44 was detected as endogenous control for normalization) and SYBR Green Master mix. **(C)** Monocytes were treated with antagomiRs (amiRs) against miR-99b, let-7e, or miR-125a or just the poly CT sequence as control. The next day, cells were stimulated to differentiate to iDCs or R848-stim.APCs. After 24 h, cells were harvested, RNA was purified, cDNA was produced, and qRT PCR was performed. **(A–C)** Mean values and SD of three individual experiments.

To investigate the role of miRNAs in perpetuation of the functional phenotype of R848-stimulated suppressive APCs, we first compared miRNA expression profiles of R848-APCs to iDCs (Table S1 in Supplementary Material). By target prediction softwares and literature research, we identified promising upregulated as well as downregulated miRNAs and decided to concentrate on the hsa-miR-99b/let-7e/miR125a cluster as the most prominent upregulated cluster (intensity shown in Figure 1A).

### Knockdown of hsa-miR-99b/let-7e/miR-125a Cluster

After having confirmed the upregulation of the hsa-miR-99b/let-7e/miR-125a cluster by quantitative reverse transcriptase (qRT) PCRs (Figure 1B), we checked the actual influence of the miRNAs on the suppressive APC phenotype. To this end, we blocked their function by the use of miRNA-targeting amiRs. amiRs are synthetically produced constructs that contain a short RNA sequence complementary to the miRNA sequence, linked to a short DNA sequence (poly CT) that serves as vehicle and allows the transition through the cell membrane. Importantly, amiRs are fully O-methylated and thereby not stimulatory and the DNA part is PTO (phosphothioate) modified to gain enhanced stability (30) (Figure S1 in Supplementary Material). In the experimental setup, monocytes isolated from blood of healthy donors were stimulated the day after isolation with GM-CSF, IL-4, and R848 (TLR7/8 ligand) to induce R848-stimulated suppressive APC. To block the respective miRNAs, cells were pretreated with amiRs 18 h prior to stimulation. The performed qRT-PCRs show the effective and specific knockdown of amiR-targeted miRNAs. Treatment with the poly CT vehicle alone had no measurable effect on the cells in terms of miRNA expression (Figure 1C).

### amiRs Against hsa-miR-99b/let-7e/miR-125a Cluster Partly Restores T Cell Activation Ability of R848-Stimulated Suppressive APCs

**Figure 2:**
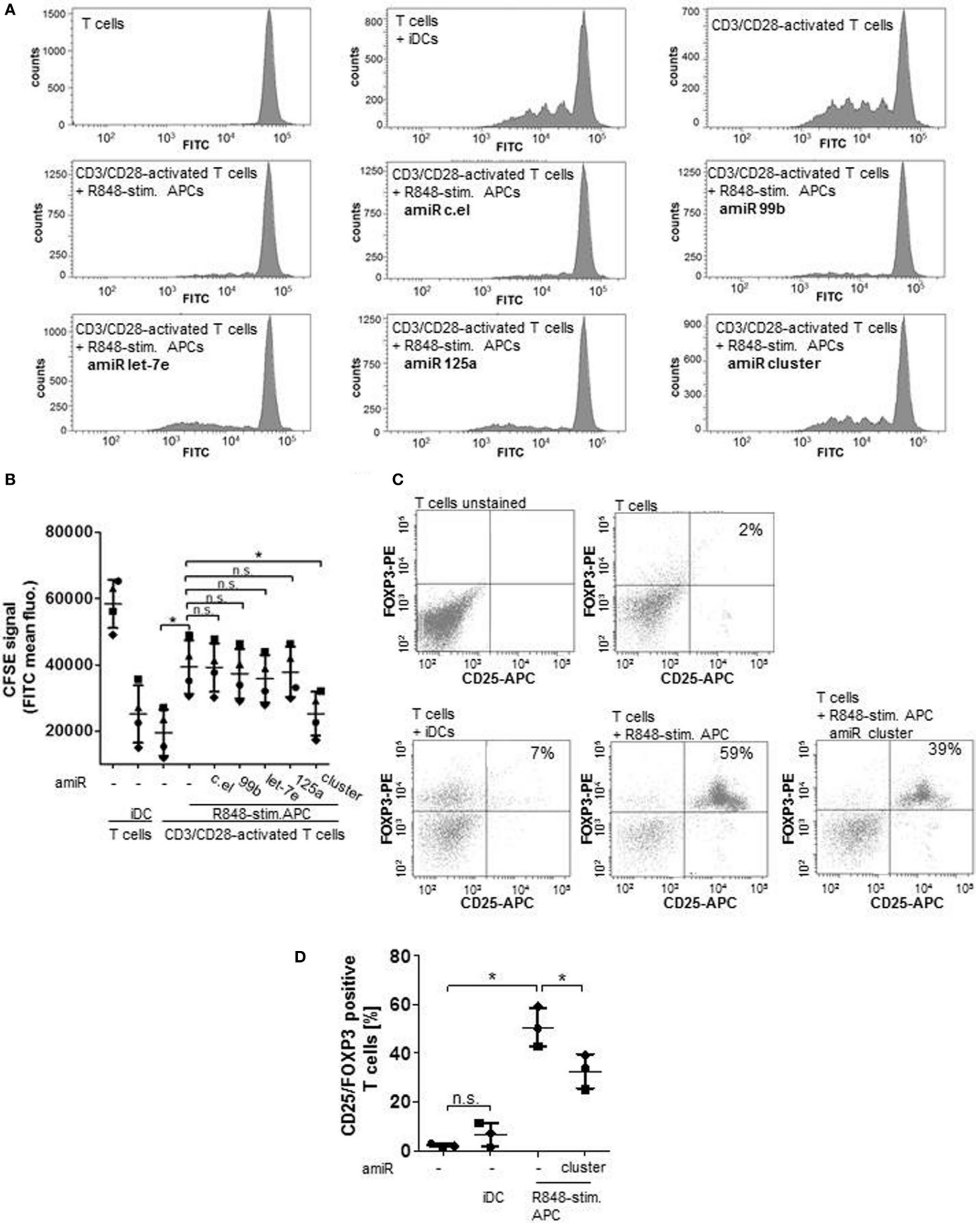
Cells were treated with antagomiRs (amiRs) against miR-99b, let-7e, or miR-125a, the whole miR cluster or against a miRNA from *C. elegans* (c.el) as control. After differentiation of cells into R848-stim.APCs, allogeneic, carboxyfluorescein succinimidyl ester (CFSE)-labeled, CD3/CD28-activated CD4^+^ T cells were added in a ratio of 2:1. Three to five days later, cell divisions were analyzed by determining the FITC signal by a FACSCanto. **(A)** Histograms that depict decreasing CFSE (FITC) signal of activated, proliferating T cells that were cultured alone or in combination with R848-stim.APCs ± amiRs as indicated. As control T cells were cultured with immature DCs (iDCs). **(B)** According to the quantification of **(A)** and three more donors. Each symbol represents one donor. **(C)** T cells were cocultured with iDCs or R848-stim.APCs ± amiR (cluster). After 5 days, T cells were double stained with anti-CD25-APC and anti-FOXP3-PE antibodies and analyzed at the FACSCanto. **(D)** Quantification of **(C)** and two more donors. Statistics: *p ≤ 0.05 by Mann–Whitney *U* test.

To address the question whether the observed upregulation of hsa-miR-99b/let-7e/miR-125a cluster is actually important for the suppressive phenotype, coculture experiments with allogeneic CFSE-labeled, T cells were performed. As the CFSE (FITC) signal decreases with cell divisions, proliferation can easily be monitored. The histograms in Figure 2A and the associated quantification in Figure 2B show, as expected, that T cells in iDC supplemented cultures and T cells that were activated with anti-CD3 and anti-CD28-coated beads proliferated intensively (Figures 2A,B). Furthermore, R848-stimulated APCs inhibited the CD3/CD28-driven proliferation of T cells significantly (Figures 2A,B). Treatment with amiRs against *C. elegans* (c.el) miRNAs (negative control) had no effect. Administration of single amiRs against miR-99b, let-7e, or miR-125a failed to diminish the T cell suppression significantly. However, inhibition of the whole hsa-miR-99b/let-7e/miR-125a cluster by amiRs restored clearly and significantly the CD3/CD28-mediated activation of cocultured T cells (Figures 2A,B). As R848-stimulated suppressive APCs do promote expansion of Tregs (16), we further analyzed the frequency of CD25/FOXP3 double-positive Tregs after 5 days of coculture. The dot blot graphs and associated quantification in Figures 2C,D reveal that the coculture of T cells and iDCs contained 7% CD25/FOXP3-positive Tregs. Suppressive APCs increased the number of Tregs to 59% and treatment with amiRs against the hsa-miR-99b/let-7e/miR-125a cluster reduced the number of Tregs significantly to 39%. These data confirmed an important role of the miR-99b/let-7e/miR-125a cluster in the T cell suppressing phenotype of R848-stimulated APC.

### hsa-miR-99b/let-7e/miR-125a Cluster-Mediated Regulation of R848-Stimulated Cytokine Production

**Figure 3:**
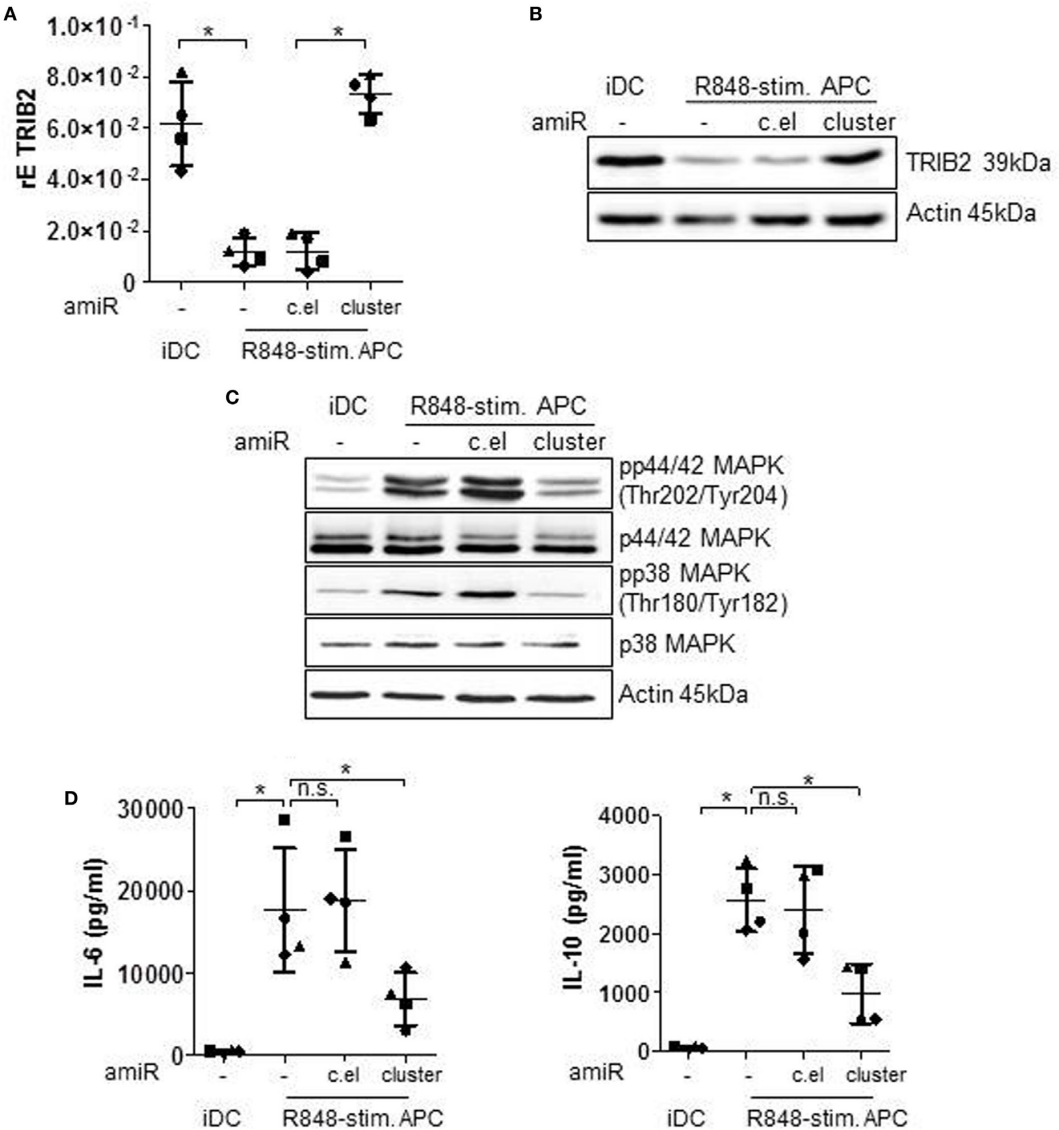
Immature DCs (iDCs) and R848-stim.APCs, treated with either a mix of antagomiRs (amiRs) against the cluster of miR-99b, let-7e, and miR-125a or amiRs against *C. elegans* miRNA (control) or untreated were analyzed as follows. **(A)** TRIB2 gene expression was analyzed by qRT PCRs. **(B,C)** For Western blot analysis, equal amounts of protein lysates were blotted and probed with antibodies against TRIB2 **(B)**, phosphorylated and total p44/42 mitogen-activated protein kinase (MAPK) and p38 MAPK **(C)** or Actin (loading control). **(D)** Supernatants were used for Enzyme-Linked Immunosorbent Assay analysis to quantify released IL-6 and IL-10 amounts. **(B,C)** Shown is one representative Western blot out of three repeats. **(A,D)** Each symbol represents one donor. Shown is mean and SD. Statistics: *p ≤ 0.05 by Mann–Whitney *U* test.

According to target prediction softwares one direct target of the hsa-miR-99b/let-7e/miR-125a cluster is TRIB2 (Tribbles pseudokinase 2). The pseudokinase TRIB2 acts as scaffold protein and mediates ubiquitination of substrates (27). Thereby TRIB2 negatively regulates MAPkinase signaling (31), the cascade that is a hallmark of R848-stimulated APCs. The qRT PCR analyses in Figure 3A show that TRIB2 is significantly downregulated in R848-stimulated APCs and the Western blot data (Figure 3B) confirm that result on protein level. Treatment with amiRs against hsa-miR-99b/let-7e/miR-125a restored the expression of TRIB2 to an expression level comparable to iDCs (Figures 3A,B). The control amiR c.el induced no measurable effect on TRIB2. Furthermore, we checked the phosphorylation and thereby activation status of MAPkinase family members p42/44 and p38. p42/44 and p38 phosphorylation was downregulated after amiR cluster treatment (Figure 3C) corroborating the restored TRIB2 expression. The reduced MAPkinase signaling correlated with a significant reduction of the MAPkinase-inducible cytokines IL-6 and IL-10 (Figure 3D).

### hsa-miR-99b/let-7e/miR-125a Cluster-Mediated Regulation of STAT3 Transcription Factor

**Figure 4:**
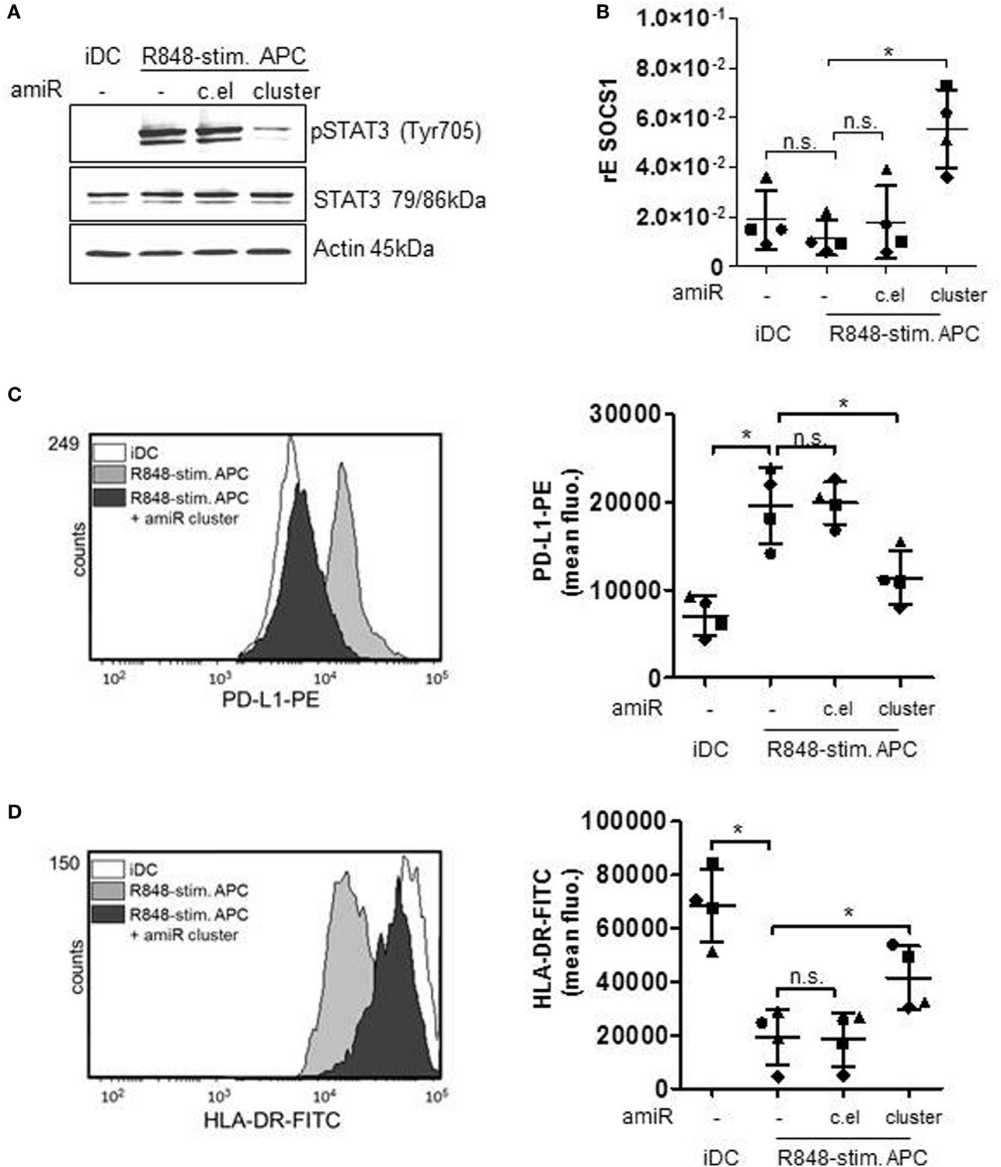
Monocytes were either treated with antagomiR (amiR) mix against miR-99b, let-7e, and miR-125a cluster (amiR cluster), amiR against c.el or left untreated. The next day cells were differentiated for 72 h into immature DCs (iDCs) (GM-CSF/IL-4) or R848-stim.APCs (GM-CSF/IL-4/R848). **(A)** Lysates were produced and applied for Western blot analyses for the detection of phosphorylated and total signal transducer and activator of transcription (STAT) 3. Actin was detected as loading control. Shown is one blot as representative example out of three. **(B)** RNA was isolated, cDNA produced, and qRT PCRs performed to analyze mRNA levels of suppressor of cytokine signaling-1 (SOCS1). **(C,D)** Cells were stained with specific antibodies against programmed death-ligand 1 (PD-L1) **(C)** or HLA-DR **(D)** and analyzed by flow cytometry. Overlays were produced by WEASEL software. Associated quantification displays four donors with mean and SD. Statistics: *p ≤ 0.05 by Mann–Whitney *U* test.

According to our previous work, the high amounts of induced IL-6 are responsible for a STAT3-induced upregulation of suppressing factors and eventually for the dampening of cocultured T cell responses through PD-L1 and IDO (17). Accordingly, the reduction of released cytokines IL-10 and IL-6 should result in a less pronounced cytokine receptor-mediated phosphorylation of STAT3. Figure 4A shows that amiR cluster treatment indeed strongly reduced STAT3 activation in R848-stimulated suppressive APCs. Notably, the amount of pSTAT3 downregulation through amiR cluster treatment was higher as expected from the cytokine data shown in Figure 3D. amiR cluster-treated cells released less cytokines but still more than iDCs. This suggested that further miRNA-mediated pathways for pSTAT3 regulation exist. Indeed, SOCS1, a negative feedback inhibitor of STAT3 (32), is a predicted target of let-7e. Figure 4B shows that SOCS1 is slightly downregulated in R848-stimulated APCs compared with iDCs. Moreover, treatment with amiR cluster clearly restored and augmented SOCS1 expression beyond to the levels observed in iDCs. These data suggest that hsa-miR-99b/let-7e/miR-125a cluster does not only boost activation of STAT3 but additionally stabilizes the activated form of the transcription factor through inhibiting its negative regulation.

### Regulation of Immunosuppressive Factors Through STAT3

**Figure 5:**
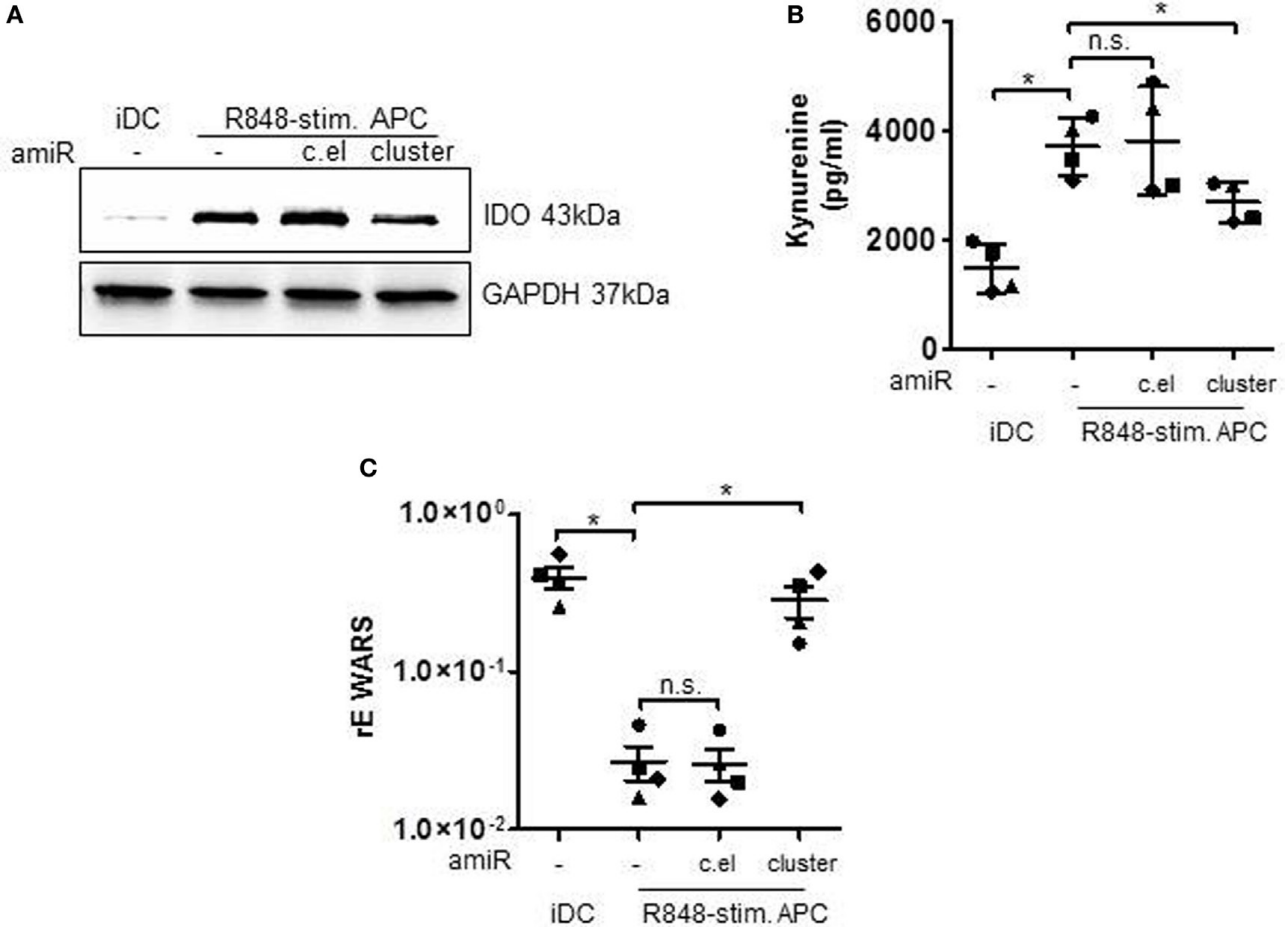
Immature DCs (iDCs) and R848-stimulated APCs, treated with either antagomiRs (amiRs) against miRNA cluster (miR-99b/let-7e/miR-125a) or against c.el miRNA (control) or untreated were analyzed in the following way. **(A)** Lysates were produced and Western blots for the detection of indolamin-2,3-dioxygenase (IDO) were performed. GAPDH served as loading control. Depicted is one example out of three experiments with similar results. **(B)** The amount of IDO-generated kynurenine was measured in the supernatant of the cells. **(C)** RNA was isolated, cDNA generated, and qRT PCRs with sequence-specific primers for WARS and SYBRGreen were performed. Results were normalized against Actin. **(B,C)** Each symbol represents one donor. Shown is mean and SD. Statistics: *p ≤ 0.05 by Mann–Whitney *U* test.

In the following, we checked whether the regulation of MAPkinase signaling and the resulting influence of STAT3 transcription factor would actually result in a modulated expression of STAT3-dependent T cell suppressive factors. Therefore, we treated primary cells with the amiR cluster and checked for suppressive factors. First, we performed flow cytometry analyses of PD-L1 surface expression. PD-L1 binding of PD-1 on T cells suppresses T cell proliferation (12, 33) according to recent publications mainly through inhibiting the CD28 costimulatory pathway (34). The histogram overlay and associated quantification show that amiR cluster treatment resulted in significantly less PD-L1 expression on R848-stimulated suppressive APCs (Figure 4C). A further characteristic of suppressive APCs is a distinct reduction of HLA-DR expression which is most probably due to the IL-6/pSTAT3 axes (35). In our flow cytometry analyses, inhibition of the miR cluster restored HLA-DR expression on R848-stimulated APCs significantly, although not to the level presented on iDCs (Figure 4D). Moreover, we determined an influence of the miR cluster on IDO. The enzyme IDO initiates tryptophan catabolism to *N*-formyl-kynurenine which can inhibit T cell activation and induce Tregs (36, 37). IDO had been identified as an essential factor in T cell suppression through R848-stimulated suppressive APCs (17). Figure 5A shows that the induction of IDO can be inhibited by amiR treatment, albeit not completely. Furthermore, the supernatants of amiR cluster-treated cells contained significant less IDO-generated kynurenine (Figure 5B). Whereas the miR-mediated regulation of IDO could be an indirect effect mediated through pSTAT3 (38), the reduction of kynurenine could be caused through the tryptophanyl-tRNA synthetase WARS. WARS competes with IDO for tryptophan (39) and according to the data in Figure 5C. WARS is clearly downregulated in suppressive APCs through the hsa-miR-99b/let-7e/miR-125a cluster.

## Discussion

Recently, we showed that IL-6 boosted by IL-1β reprograms differentiating blood monocytes through the key transcription factor STAT3 toward a suppressive myeloid cell type (17). Notably, key regulators of immunity such as STAT3 are under tight control. Activated through cytokine receptor signaling, the transcription factor terminates its own action through induction of negative feedback inhibitors such as SOCS1 (40).

A prolonged activation of STAT3 is a hallmark of the R848-stimulated suppressive APC described here. Obviously, this stabilized STAT3 activation hints to alterations of the negative regulatory mechanisms of STAT3 expression. Therefore, we set out to clarify the underlying mechanism. Here, we show that the miRNA cluster hsa-miR-99b/let-7e/miR-125a stabilizes activation of the key regulator STAT3, which in turn facilitates the induction of immunosuppressive factors, responsible for T cell inhibition.

Non-coding RNAs, such as miRNAs and small-interfering (si)RNAs, which are implicated in post-transcriptional RNA silencing, represent most of the human transcriptome. Through comparative genomics analyses and high-throughput experimental studies, the high number of potential direct miRNA targets has been revealed. Typically, one single miRNA, with some reported exceptions, does not alter the protein expression level of a targeted mRNA to a vast extent. Rather, distinct miRNAs act in conjunction and thus fine tune a respective mRNA expression in the cell. Therefore, it is obvious to study the influence of miRNA clusters rather than individual miRNAs.

In this study, we concentrated on the hsa-miR-99b/let-7e/miR-125a cluster. The three miRNAs are highly upregulated in R848-stimulated suppressive APCs. The expression of the cluster is probably controlled by TLR7/8-mediated activation of NF-κB since NF-kB p65 binds to its promoter and inhibition or depletion of NF-κB results in impaired expression of the cluster (25). Although related miRNAs are transcribed often as one primary RNA transcript, the level of individual mature miRNAs might differ during maturation steps. In R848-induced suppressive APC, we found all three mature miRNAs of the miRNA cluster upregulated compared with iDCs. Therefore, it was consistent to inhibit the individual miRNAs of whole hsa-miR-99b/let-7e/miR-125a cluster.

An upregulation of the hsa-miR-99b/let-7e/miR-125a cluster or single miRNA cluster members was described in innate immune cells under different inflammatory conditions (41, 42). The miR cluster was shown to be induced through LPS stimulation in monocytes (43) and several publication show the TLR4- and TLR2-dependent induction of miR-125a and let-7e in macrophages (44–46). Widely consistent the infection-induced upregulation of hsa-miR-99b/let-7e/miR-125a cluster members is associated with downregulation of the response and assumed to prevent the organism from an overwhelming immune reaction. Several mechanisms have been proposed to account for this. For example, Androulidaki et al. suggest a negative feedback mechanism and show that TLR4-induced let-7e targets TLR4, terminating TLR-mediated cytokine production (44). Furthermore, miR-125a is hypothesized to suppress classical activation of macrophages through LPS while promoting the alternative activation to M2 macrophages (47). This would initiate the regeneration phase after the T effector cell response. An additional hypothesis proposes that cytokine and cytokine receptor targeting, as shown for *Mycobacterium tuberculosis*-induced miR-99b, should be seen as immune evasion strategy of the bacterium (46).

Our study confirms the role of hsa-miR-99b/let-7e/miR-125a cluster in modulating innate immune cells after TLR activation although by so far undescribed mechanisms that regulate the STAT3-dependent functional phenotype of R848-stimulated suppressive APCs. First of all, the miR cluster boosts TLR-induced IL-6 production through targeting Mapkinase inhibitor TRIB2. As a consequence, cytokine receptor-transduced activation of STAT3 is enhanced. Indeed, the pseudokinase TRIB2 has been recognized as important regulator of TLR-mediated monocyte activation through regulation of Mapkinase signaling. Eder et al. showed that TRIB2 inhibits extracellular signal-regulated kinase (ERK) and Jun kinase activation by binding mitogen-activated protein kinase (MAPK) complexes, MKK7 and MEK1 in monocyte. Stimulation of monocytes with LPS downregulated TRIB2 expression and enhanced monocyte activation assessed by enhanced production of IL-8 (27). In our system, the inhibition of hsa-miR-99b/let-7e/miR-125a cluster restored the downregulated expression of TRIB2 and thus reduced the activation of p42/44 (ERK) and p38. Eventually, the impaired R848-mediated MAPK activation after miRNA inhibition led to a significantly lower production of IL-6 and consecutively to a diminished activation of STAT3.

Moreover, hsa-miR-99b/let-7e/miR-125a cluster stabilizes the activated state of the transcription factor through targeting the STAT3 inhibitor SOCS1. SOCS1 is a key regulator of cytokine signaling and is crucial for maintaining balance in the immune system. However, whether SOCS1 in myeloid cells facilitates or restricts tolerance is discussed controversially. Once LPS-stimulated SOCS1 arrests TLR-mediated NF-κB signaling and therefore conditions LPS tolerance (48). On the other hand, SOCS1 breaks tolerance through the downregulation of STAT3 activation. For example in Hodgkin lymphoma, a high mutation rate in SOCS1 gene correlates with hyperactivation of JAK2 a gained PD-L1 expression (49, 50). As PD-L1 binding to its receptor PD-1 on T cells inhibits lymphocyte activation loss-of-function mutation of SOCS1 would mediate therefore a tolerogenic myeloid phenotype. So far, the most studied miRNA that targets SOCS1 is miRNA 155 (51–53). Interestingly in a study investigating the miRNA profile of monocyte-derived immunogenic DCs, miRNA 155 was found to be strongly upregulated (54). Whether this upregulation affects SOCS1 expression was not evaluated. According to our predicted target analyses, the 3′UTS of SOCS1 can be also bound by let-7e which suggests a direct effect of the cluster. Our data show a lower expression of SOCS1 in R848-stimulated APCs in comparison to iDCs that can be restored through inhibition hsa-miR-99b/let-7e/miR-125a cluster. Presumably, this restorage of SOCS1 contributes to the lower STAT3 activation and finally the lower induction of immunosuppressive factors PD-L1 and IDO.

Finally, hsa-miR-99b/let-7e/miR-125a cluster facilitates the immunosuppressive R848-stimulated APC phenotype by additionally promoting production of IDO-generated kynurenine through targeting WARS, the direct competitor of IDO in terms of Trp availability.

In summary, our study highlights the role of miRNA-mediated gene regulation in differentiation and preservation of a suppressive APC phenotype. Our data show that inhibition of the whole hsa-miR-99b/let-7e/miR-125a cluster, but not the knockdown of single cluster members, significantly diminishes the T cell suppressive character of R848-stimulated suppressive APCs. Key miRNA targets as TRIB2, SOCS1, and WARS have been identified. This points to an important interplay between the three miRNAs and reveal the requirement for more detailed studies in the future. As STAT3 dysregulation contributes to many pathological situations during infectious or neoplastic diseases (55) a broader understanding of miRNAs–STAT3 interactions could help to manipulate them for developing novel therapeutic approaches.

## Ethics Statement

This study was carried out in accordance with the recommendations of the ethics committee of the Medizinische Fakultät Heidelberg with written informed consent from all subjects. All subjects gave written informed consent in accordance with the Declaration of Helsinki. The study (taking of blood samples from healthy donors and treatment of blood leukocytes with microbial stimuli) was reviewed and approved by the ethics committee of Medizinische Fakultät Heidelberg.

## Author Contributions

KH and DH designed the study with essential input from M-EE, SW, and KB. KH and DH wrote the manuscript. DH, AS, DS, and FE performed the experiments. All the authors read the manuscript.

## Conflict of Interest Statement

The authors declare that the research was conducted in the absence of any commercial or financial relationships that could be construed as a potential conflict of interest.
