# Supplementary material for: Hsa-miR-99b/let-7e/miR-125a Cluster Regulates Pathogen Recognition Receptor-Stimulated Suppressive Antigen-Presenting Cells
Source: Front Immunol. 2018 Jun 18;9:1224. doi: 10.3389/fimmu.2018.01224 (PMC6015902; doi:10.3389/fimmu.2018.01224)
Supplement: Supplementary file 1 [file data_sheet_1.PDF]

Supplementary Material

Hsa-miR-99b/let-7e/miR-125a cluster regulates pathogen recognition receptor-stimulated suppressive APCs

Dagmar Hildebrand[1], Mariel-Esther Eberle[1], Sabine Wölfle[1], Franziska Egler[1], Delal Sahin[1], Aline Sähr[1], Konrad A. Bode[1], Klaus Heeg[1,2]

[1]Medical Microbiology and Hygiene, Centre for Infectious Diseases, University Hospital Heidelberg, Heidelberg, Germany

[2] DZIF

*** Correspondence:** Dagmar Hildebrand, Ph.D., Medical Microbiology and Hygiene, Department of Infectious Diseases, University Hospital Heidelberg, Im Neuenheimer Feld 324, Heidelberg 69120, Germany

dagmar.hildebrand@med.uni-heidelberg.de

Supplementary Figures

AntagomiR (amiR) constructs:

DNA PolyCT PTO - 2X (C3 carbon chain linker) - **RNA fully 2'-O-methylated**

amiR 99b 5p

5' – CTC TCT CTC TCT CTC TCT CTC T– 2 x $(CH_2)_3$ – **3' – GUG GGC AUC UUG GCU GGA ACG – 5'**

amiR 125a 5p

5' – CTC TCT CTC TCT CTC TCT CTC T– 2 x $(CH_2)_3$ – **3' – AGG GAC UCU GGG AAA UUG GAC ACU–5'**

amiR let-7e

5' – CTC TCT CTC TCT CTC TCT CTC T– 2 x $(CH_2)_3$ – **3' ACUCCAUCCUCCAACAUAUCA– 5'**

Suppl Fig.1 Synthetically produced AntagomiRs (amiRs) constructs amiR 99b, amiR 125a and amiR let-7e. Constructs contain a short RNA sequence complementary to the target miRNA sequence, linked to a short DNA sequence (poly CT) that serves as vehicle and allows the transition through the cell membrane. amiRs are fully o-methylated.
