# Supplementary material for: Hsa-miR-99b/let-7e/miR-125a Cluster Regulates Pathogen Recognition Receptor-Stimulated Suppressive Antigen-Presenting Cells
Source: Front Immunol. 2018 Jun 18;9:1224. doi: 10.3389/fimmu.2018.01224 (PMC6015902; doi:10.3389/fimmu.2018.01224)
Supplement: Supplementary file 2 [file table_1.PDF]

Suppl. Table 1 **miRNA expression data**

TargetID	R848-stim APC.mean (n=3)	R848 std	iDC mean (n=3)	iDC std
hsa-let-7e-5p	4589,253	471,574	243,854	56,914
hsa-miR-125a-5p	1497,746	361,230	108,357	29,993
hsa-miR-99b-5p	1190,160	192,272	93,340	17,636
hsa-miR-4281	18241,000	7356,149	1439,250	541,582
hsa-miR-146a-5p	17309,504	2634,565	1408,961	379,337
hsa-miR-4281	15989,466	6146,776	1317,891	508,369
hsa-miR-155-5p	4645,869	289,043	417,691	26,449
hsv2-miR-H6	1848,194	640,535	173,936	49,639
hsa-miR-3663-3p	2326,077	893,574	242,289	79,753
hsa-miR-155-5p	1515,412	485,344	179,694	20,808
hsv2-miR-H6	1115,738	281,666	136,984	25,918
hsa-miR-3663-3p	2291,266	975,798	289,457	55,858
hsa-miR-9-3p	447,410	68,981	60,518	5,723
hsa-miR-3665	2978,502	1453,023	458,532	106,045
hsa-miR-9-3p	332,122	63,176	54,450	5,583
hsa-miR-9-5p	358,418	117,403	59,541	7,070
hsa-miR-1915-3p	2454,788	851,336	411,644	109,056
hsa-miR-1915-3p	2358,064	746,497	396,894	101,779
hsa-miR-3665	1670,528	743,921	313,787	64,666
hsa-miR-1268a	869,739	633,500	189,285	27,154
hsv1-miR-H18	435,741	169,757	98,068	17,898
hsa-miR-762	1083,087	230,533	249,717	26,569
hsa-miR-1290	2067,182	1693,676	502,432	116,475
hsa-miR-150-3p	368,454	95,407	102,223	18,042
hsa-miR-9-5p	176,993	62,566	49,494	6,353
hsa-miR-3648	262,052	131,362	74,930	13,972
hsa-miR-3656	354,732	206,060	104,161	10,771
hsa-miR-147b	172,983	12,524	51,800	8,078
hsv1-miR-H18	411,558	151,292	123,498	18,275
hsa-miR-150-3p	297,925	68,421	91,800	9,575
hsa-miR-3656	378,798	216,880	117,204	11,774
hsa-miR-451a	321,293	226,083	99,564	26,461
hsa-miR-1225-5p	1836,723	763,705	577,265	92,069
hsa-miR-3648	288,701	149,816	93,190	20,125
hsa-miR-718	304,801	147,511	99,376	16,900
hsv2-miR-H10	579,842	257,667	194,719	38,362
hsa-miR-150-3p	272,898	76,858	91,995	11,011
hsa-miR-2861	1126,159	598,962	384,260	83,868
hsa-miR-1225-5p	1625,431	649,249	557,344	95,935
hsa-let-7i-5p	30441,818	6491,181	10439,517	1356,941
hsa-miR-181b-5p	354,579	69,608	121,795	10,905
hsa-let-7i-5p	22691,133	6237,248	7935,543	519,015

hsa-miR-193a-3p	1037,478	178,495	364,012	56,920
hsa-miR-150-3p	270,638	77,396	95,250	11,416
hsa-miR-451a	235,092	158,953	82,819	21,461
hsa-miR-630	1367,239	1091,970	485,353	262,104
hsa-miR-940	373,083	98,557	132,549	10,563
hsa-miR-150-5p	2741,060	1457,650	985,027	508,171
hsa-miR-630	1295,367	1080,154	467,045	261,320
hsa-miR-4327	243,630	66,413	88,037	10,686
hsa-miR-34b-5p	599,055	203,567	218,340	21,858
hsa-miR-150-5p	1353,049	510,799	497,597	232,831
hsa-miR-34b-5p	762,523	262,725	286,914	38,643
hsa-miR-222-3p	409,804	19,827	154,380	23,139
hsa-miR-29a-3p	27084,244	2728,387	10235,077	1444,352
hsa-miR-1268a	352,641	154,958	133,944	14,673
hsa-miR-4327	242,305	72,402	92,988	7,956
hsa-miR-1207-5p	989,755	449,304	385,142	68,355
hsa-miR-146b-5p	5875,307	489,375	2287,522	104,066
hsa-miR-181b-5p	235,629	48,244	92,468	8,522
hsa-miR-3188	150,129	58,888	59,581	7,846
hsv2-miR-H10	390,561	163,644	155,091	27,161
hsa-miR-940	257,558	93,486	105,932	9,986
hsa-miR-181c-5p	226,611	46,712	93,250	6,206
hsa-miR-29a-3p	12332,147	484,496	5145,701	562,885
hsa-miR-4322	140,818	12,838	58,775	8,684
hsa-miR-29b-3p	7236,973	757,140	3028,027	249,225
hsa-miR-1207-5p	844,273	368,019	355,702	66,299
hsa-miR-4322	136,752	18,830	57,671	7,259
hsa-miR-3188	137,353	47,471	59,118	6,563
hsa-miR-29b-3p	27513,306	2243,947	11973,532	866,188
hsa-miR-2861	375,862	168,191	164,769	36,726
hsa-miR-7-5p	554,562	96,251	243,506	27,042
hsa-miR-193a-3p	347,768	93,641	152,957	19,919
hsa-miR-1246	10352,407	7487,980	4567,485	2671,577
hsa-miR-34a-5p	9388,887	1875,185	4145,441	607,796
hsa-miR-371a-5p	157,548	76,466	72,451	10,377
hsa-miR-638	992,188	459,166	460,042	90,608
kshv-miR-K12-3	668,762	168,739	313,989	32,517
hsa-miR-638	525,708	208,872	248,247	57,212
hsa-miR-125a-3p	253,574	63,764	121,193	16,465
hsa-miR-3937	121,876	21,869	58,344	6,814
hsa-miR-125a-3p	257,491	77,013	125,105	20,032
hsa-miR-181a-5p	1999,821	441,952	975,339	161,655
hsa-miR-135a-3p	318,916	164,483	155,629	24,460
hsa-miR-642b-3p	350,515	137,574	172,096	23,786
hsa-miR-3196	593,731	154,290	292,342	25,300
hsa-let-7i-3p	185,110	103,140	92,939	7,270
hsa-miR-642b-3p	328,312	129,537	165,381	23,932

hsv2-miR-H25	493,814	178,938	248,997	19,126
hsa-miR-3937	119,211	18,981	60,214	5,454
hsa-miR-4271	234,085	47,648	118,486	21,838
hsa-miR-3196	452,918	120,215	230,320	13,802
hsa-miR-4271	250,301	52,543	128,836	15,860
hsa-miR-34a-5p	5096,861	990,127	2668,009	546,032
hsv2-miR-H25	355,404	119,226	186,160	17,511
hsa-miR-193a-5p	132,193	17,937	69,287	7,454
hsa-miR-125a-3p	202,487	62,143	106,986	15,009
hsa-miR-7-5p	174,080	22,891	92,574	11,481
hsa-miR-4270	311,264	164,379	166,999	52,720
hsa-let-7i-3p	134,723	51,491	72,995	6,913
hsa-miR-762	262,232	63,959	142,930	12,640
hsa-miR-21-3p	795,632	277,355	436,718	23,511
hsa-miR-181a-5p	980,203	290,994	540,838	60,492
hsa-miR-181c-5p	130,385	26,489	72,033	7,107
hsv2-miR-H24	345,548	40,908	191,821	24,168
hsa-miR-135a-3p	247,657	113,873	137,587	29,722
hsa-miR-192-5p	162,655	33,406	90,722	9,199
hsa-miR-629-3p	167,468	59,260	93,997	18,347
hsa-miR-1181	217,993	51,324	123,045	12,048
hsa-miR-4257	141,179	26,107	80,023	6,924
hsa-miR-34b-3p	343,242	100,984	194,695	88,115
hsa-miR-34b-3p	351,500	106,411	200,183	89,608
hsa-miR-181d	132,613	16,579	75,982	8,113
hsa-miR-147b	81,480	9,736	46,685	5,048
hsa-miR-21-3p	1360,845	396,271	786,199	40,251
hcmv-miR-UL70-3p	122,302	39,612	70,709	10,524
hsv2-miR-H24	300,243	37,395	173,839	26,613
hsv1-miR-H17	126,048	30,883	73,101	12,094
hsa-miR-1181	181,000	44,509	105,390	9,962
hsa-miR-4270	287,063	152,827	167,815	45,852
hcmv-miR-UL70-3p	109,129	30,497	64,271	11,856
hsa-miR-629-3p	185,836	54,561	109,626	16,502
hsa-miR-134	164,487	56,882	97,132	14,106
hsa-miR-361-3p	566,653	43,445	336,105	28,870
hsa-miR-150-5p	276,393	67,542	164,158	63,583
hsa-miR-3170	131,623	29,833	78,397	10,796
hsa-miR-629-3p	164,599	48,355	98,299	14,593
hsa-miR-34a-3p	248,188	97,767	149,261	16,651
hsa-miR-549	72,934	8,008	43,999	5,208
hsa-miR-371a-5p	116,410	41,816	70,323	7,427
hsa-miR-3170	121,621	27,136	73,589	9,836
hsa-miR-572	204,892	66,846	124,206	25,862
hsa-miR-134	158,459	49,679	96,569	16,942
hsa-miR-3679-5p	439,909	194,902	269,275	65,595
hsa-miR-29c-3p	9344,994	767,558	5732,118	563,742

hsa-miR-629-3p	157,338	55,543	97,837	15,452
hsa-miR-663a	127,448	23,096	79,432	7,743
kshv-miR-K12-3	301,507	67,743	189,230	30,643
hsa-miR-144-3p	83,617	35,674	52,490	6,492
hsa-miR-146b-5p	18330,800	895,252	11548,577	832,905
hsa-miR-194-5p	109,278	9,077	69,027	8,379
hsa-miR-449b-3p	315,216	73,786	200,504	93,493
hsa-miR-144-3p	79,156	32,186	50,572	7,360
hsa-miR-373-5p	71,902	13,804	46,023	6,145
hsa-miR-192-5p	115,700	12,995	74,573	8,707
kshv-miR-K12-12*	479,445	92,454	309,526	135,885
hsa-miR-3935	237,665	58,911	153,502	46,904
hsa-miR-3679-5p	251,075	104,503	163,500	37,343
hsa-miR-874	262,606	42,483	171,068	12,509
hsa-miR-3935	234,643	55,583	152,908	48,834
hsa-miR-449b-3p	260,266	56,072	169,754	78,588
hsa-miR-1249	184,349	32,640	120,509	15,957
hsa-miR-548d-5p	99,133	29,085	65,263	7,188
hsa-miR-3646	172,088	41,603	113,685	23,620
hsa-miR-3923	134,162	47,853	88,993	16,130
hsa-miR-548y	94,270	25,651	62,824	6,550
hsa-miR-1909-5p	138,624	37,537	92,422	10,893
hsa-miR-2276	102,543	22,682	68,433	9,368
hsa-miR-210	2138,736	547,305	1429,003	949,704
hsa-miR-557	84,150	17,174	56,291	7,735
hsa-miR-29c-3p	7554,157	619,607	5066,241	498,976
kshv-miR-K12-10a*	280,568	59,593	188,256	87,348
hsa-miR-2276	101,495	24,180	68,182	9,027
hsa-miR-549	66,531	6,348	44,870	2,744
hsv1-miR-H8	150,398	53,117	101,632	13,600
hsa-miR-139-3p	119,274	13,588	80,655	10,290
hsa-miR-631	144,988	29,435	98,076	25,976
hsa-miR-3195	573,574	158,984	389,713	23,697
hsa-miR-137	66,275	7,771	45,080	6,723
hsa-miR-101-3p	1906,628	370,302	1298,407	107,888
hsa-miR-3117-3p	103,858	30,636	70,728	12,938
kshv-miR-K12-10a*	246,703	50,518	168,511	74,129
hsa-miR-548y	93,767	25,586	64,076	4,523
hsa-miR-371a-5p	102,717	29,606	70,206	5,747
hsa-miR-663a	89,939	16,398	61,785	5,917
kshv-miR-K12-12*	429,536	81,171	295,344	130,396
hsa-miR-1972	111,969	25,587	77,214	9,452
hsa-miR-654-3p	163,408	26,207	112,924	37,407
hsa-miR-4257	88,152	12,964	61,153	7,147
hsa-miR-19b-1-5p	104,478	22,552	72,485	10,976
hsa-miR-3646	166,261	46,054	115,352	22,788
hsa-miR-1249	165,758	23,450	115,135	16,316

hsa-miR-3117-3p	104,471	27,446	73,204	11,722
hsa-miR-3923	143,700	50,826	100,719	17,550
hsa-miR-215	95,262	8,825	66,773	6,761
hsa-miR-1909-5p	127,512	30,422	89,527	13,154
hsa-miR-3162-5p	1792,193	397,697	1258,703	139,350
hsa-miR-139-5p	67,946	7,612	48,012	5,783
hsa-miR-548d-5p	102,897	30,926	73,169	7,174
hsa-miR-548d-5p	114,859	34,818	81,692	7,975
hsa-miR-557	84,560	17,726	60,277	8,297
hsa-miR-19b-1-5p	99,817	23,967	71,322	17,563
hsa-miR-137	61,620	7,162	44,067	5,063
hsa-miR-654-3p	146,309	17,907	105,107	30,847
hsa-miR-29b-1-5p	141,675	9,538	101,792	15,460
hsa-miR-335-3p	119,514	12,076	86,109	15,338
hsa-miR-181d	87,031	8,408	62,724	7,236
hsa-miR-766-3p	253,945	44,157	183,486	27,044
hsa-miR-1281	302,283	57,456	219,583	49,107
hsa-miR-3923	131,270	41,648	95,408	18,727
hsa-miR-4259	106,453	32,313	77,408	13,118
hsa-miR-466	280,090	31,877	204,739	95,785
hsa-miR-1299	96,625	19,249	70,694	7,207
hsa-miR-4254	326,282	28,992	239,208	122,325
hsa-miR-296-5p	106,389	13,063	78,049	9,597
hsa-miR-1224-5p	107,055	21,123	78,566	9,932
hsa-miR-1825	201,418	38,716	148,217	32,425
hsa-miR-1281	240,172	45,095	176,810	43,812
hsa-miR-877-5p	64,279	9,886	47,439	1,703
hsa-miR-215	87,964	7,418	64,922	6,797
hsa-miR-222-3p	71,811	8,305	53,080	8,294
hsa-miR-197-3p	744,669	102,130	552,083	159,420
hsa-miR-877-3p	211,884	41,267	157,141	42,002
hsa-let-7i-3p	83,504	21,090	62,027	6,335
hsa-miR-194-5p	73,239	6,825	54,403	7,273
hsa-miR-101-3p	1016,207	215,267	755,459	111,710
hsa-miR-466	228,224	18,844	169,696	76,682
hsa-miR-4298	123,528	30,224	92,020	10,568
hsa-miR-328	170,888	32,362	127,310	23,133
hsv1-miR-H17	82,428	10,015	61,492	7,169
hsa-miR-548d-5p	107,424	26,139	80,183	8,168
hsa-miR-766-3p	207,826	41,003	155,301	23,868
kshv-miR-K12-10b	96,503	10,533	72,119	7,227
hsa-miR-1825	224,472	36,854	167,757	30,784
hsa-miR-1910	85,678	20,662	64,062	7,347
hsa-miR-449a	79,877	17,789	59,747	5,966
hsa-miR-1910	90,218	19,586	67,501	6,969
hsa-miR-3162-5p	1715,730	301,572	1291,742	145,973
hsa-miR-631	97,406	16,948	73,638	10,976

hsa-miR-4254	328,499	29,354	248,861	115,075
hsa-miR-548am-5p	108,030	28,217	81,928	6,029
hsa-miR-149-3p	61,295	7,835	46,722	6,474
hsa-miR-549	57,342	4,401	43,959	4,983
kshv-miR-K12-10b	64,824	7,352	49,749	6,708
hsa-miR-1228-3p	140,798	9,980	108,203	10,896
hsa-miR-483-3p	218,683	30,410	168,203	36,286
hsa-miR-877-3p	147,801	24,962	113,725	26,384
hsa-miR-363-3p	274,582	22,417	211,576	39,557
hsv2-miR-H23	79,327	15,283	61,229	8,908
kshv-miR-K12-12*	329,183	57,972	254,197	114,069
hsa-miR-548i	64,139	10,905	49,529	4,471
hsa-miR-1224-5p	104,183	18,688	80,548	9,926
hsa-miR-466	203,614	13,048	157,435	73,000
hsa-miR-1296	166,373	21,537	128,835	47,783
hsv2-miR-H23	78,932	20,696	61,181	8,201
hsa-miR-197-3p	463,038	61,308	359,637	129,573
hcmv-miR-US5-1	53,584	6,091	41,664	4,401
hsa-miR-181c-3p	101,481	21,885	78,958	15,990
hsa-miR-554	71,232	11,640	55,483	7,884
hsa-miR-885-5p	215,601	21,958	168,438	54,027
hsa-miR-652-3p	566,765	108,049	443,039	44,045
hsa-miR-335-3p	112,830	17,011	88,220	16,491
hsa-miR-885-5p	229,429	20,683	179,714	59,809
hsa-miR-671-5p	88,902	15,511	69,684	5,878
hsa-miR-937	173,393	16,559	135,914	32,428
hsa-miR-19b-1-5p	111,415	26,260	87,341	9,879
hsa-miR-498	61,497	9,070	48,214	4,942
hsa-miR-877-3p	141,435	24,309	110,999	16,111
hsa-miR-3620	97,574	17,139	76,697	10,054
hsa-miR-195-3p	98,820	15,167	77,694	7,710
hsa-miR-554	71,855	13,338	56,560	9,106
hsv2-miR-H9-3p	169,234	20,321	133,231	31,934
hsa-miR-4274	91,629	6,302	72,160	10,237
hsa-miR-887	76,729	15,647	60,431	11,073
hsa-miR-3622a-5p	59,557	7,772	46,918	6,055
hsa-miR-766-3p	176,742	31,077	139,420	36,501
hsa-miR-720	33559,663	2110,946	26490,147	5386,519
hsa-miR-3195	272,760	56,806	215,406	15,811
hsa-miR-335-3p	111,265	13,303	87,966	18,074
hsa-miR-193a-5p	68,704	6,150	54,410	7,097
hsa-miR-574-3p	745,503	169,330	590,477	97,437
hsa-miR-877-5p	70,282	16,021	55,757	6,228
hsa-miR-1299	90,946	17,635	72,164	6,326
hsa-miR-466	239,016	25,204	189,773	85,838
hsa-miR-210	1319,966	486,809	1049,142	675,804
hsa-miR-548q	73,036	12,175	58,139	9,667

hsa-let-7i-3p	72,401	15,447	57,651	3,381
hsv2-miR-H9-3p	167,152	20,972	133,107	32,455
hsa-miR-335-3p	119,945	15,427	95,783	19,731
hsa-miR-449c-3p	147,276	26,432	117,695	17,176
hsa-miR-1208	65,925	7,779	52,803	6,671
hsa-miR-296-5p	73,089	7,977	58,589	5,075
hsa-miR-3620	90,541	18,136	72,662	10,993
hsa-miR-146a-3p	63,781	15,654	51,196	6,818
hsa-miR-4259	77,921	16,267	62,564	7,843
hsa-miR-764	128,678	12,985	103,606	25,263
hsa-miR-548w	63,059	11,633	50,880	5,032
hsa-miR-195-3p	108,084	17,500	87,216	8,179
hsa-miR-937	188,223	20,521	151,897	36,197
hsa-miR-584-5p	78,908	5,847	63,683	13,282
hbv-miR-B2RC	82,609	19,123	66,703	9,532
hsa-miR-548i	60,476	8,852	48,844	3,217
hsa-miR-574-3p	816,371	182,458	659,713	98,093
hsa-miR-877-5p	66,523	11,507	53,805	7,214
hsv2-miR-H6*	415,933	39,410	336,420	145,224
hsv1-miR-H6-3p	309,275	36,560	250,231	86,503
hsa-miR-548q	69,521	12,457	56,271	8,401
hcmv-miR-US4	65,298	15,252	52,904	7,186
hsa-miR-363-3p	203,695	12,993	165,101	24,197
hsa-miR-4298	97,787	17,258	79,323	5,017
hsa-miR-764	134,439	17,136	109,172	31,653
hsa-miR-3940-3p	102,891	11,144	83,624	16,936
hsa-miR-548am-5p	122,483	29,831	99,662	9,980
hsa-miR-877-5p	61,922	16,533	50,470	6,635
hsa-miR-3938	56,377	4,824	45,963	6,129
hsa-miR-548w	69,179	13,512	56,434	6,442
hsa-miR-548aa	91,958	14,029	75,052	14,306
hsa-miR-937	110,438	7,331	90,208	14,669
hsa-miR-365a-3p	932,272	224,627	762,846	47,205
hsa-miR-3136-5p	92,605	19,646	75,890	10,803
hsv1-miR-H1	63,764	6,654	52,265	5,343
hsa-miR-613	58,402	6,766	47,987	5,970
hsa-miR-3136-5p	87,051	17,246	71,617	11,762
hsa-miR-1225-3p	82,594	8,917	67,988	6,312
hsa-miR-200c-5p	60,671	5,889	49,991	4,442
hsa-let-7b-3p	132,767	20,733	109,420	24,924
hsa-miR-596	49,051	5,101	40,481	6,553
hsa-let-7b-3p	130,934	19,217	108,192	22,502
hsa-miR-4312	107,477	13,099	88,818	14,548
hsa-miR-1226-5p	79,998	10,900	66,143	9,916
hsa-miR-186-5p	236,016	35,539	195,215	14,305
hsa-miR-4297	85,766	10,858	71,027	6,395
hsa-miR-1228-3p	116,195	8,584	96,237	7,347

hsa-miR-181a-3p	95,104	28,924	78,776	7,670
hsa-miR-361-3p	158,719	27,889	131,584	9,612
hsa-miR-1915-5p	51,154	7,640	42,463	5,396
hsa-miR-449c-3p	144,872	20,033	120,266	13,602
hsa-miR-3938	57,984	6,674	48,142	6,328
hsa-miR-572	60,318	9,004	50,082	6,604
hsa-miR-4319	67,679	10,485	56,200	8,250
hsa-miR-718	66,688	9,908	55,396	5,548
hsa-miR-181a-3p	110,173	30,370	91,538	9,217
hsa-miR-4269	72,269	10,198	60,049	9,464
hsa-miR-1290	92,143	23,793	76,591	11,146
hsa-miR-3132	89,334	9,306	74,262	9,807
hsa-miR-214-3p	59,931	6,065	49,841	7,346
hsa-miR-4314	63,629	10,386	52,985	5,277
hsa-miR-92b-3p	65,480	4,396	54,606	4,979
hsa-miR-887	75,027	14,305	62,580	9,634
hsa-miR-195-3p	78,857	11,427	65,872	9,659
hsa-miR-4312	96,372	9,934	80,517	12,262
hsa-miR-1225-3p	87,064	11,218	72,749	6,560
hsa-miR-548i	63,128	10,849	52,766	6,847
hsa-let-7d-3p	98,065	16,097	81,993	10,928
kshv-miR-K12-10b	47,579	5,228	39,804	3,516
hsa-miR-182-5p	72,962	7,606	61,045	14,274
hsa-miR-1226-5p	81,382	11,814	68,190	8,929
hsa-miR-1227	74,601	8,374	62,562	9,612
hsa-miR-99b-3p	68,677	6,448	57,614	9,130
hsa-let-7b-3p	141,539	14,906	118,741	26,762
hsa-miR-4274	83,854	8,784	70,363	10,269
hsa-miR-554	64,759	7,277	54,450	8,056
ebv-miR-BART7	66,774	6,227	56,155	6,447
hsa-miR-1208	62,648	9,130	52,700	5,807
hsa-miR-3610	66,802	17,944	56,232	9,894
hsv1-miR-H1*	361,288	25,644	304,235	131,707
hsa-miR-34a-3p	84,559	20,293	71,317	6,519
hsv2-miR-H5	78,328	10,913	66,080	10,667
hsa-let-7g-3p	66,415	3,980	56,091	5,423
hsa-miR-876-5p	85,456	10,897	72,204	16,610
hsa-miR-671-5p	81,286	13,138	68,683	4,611
hsa-miR-876-5p	89,042	8,890	75,239	19,029
hsa-miR-1234	104,417	7,367	88,277	11,566
hsa-miR-4290	257,657	30,179	217,904	60,991
hsa-miR-29a-5p	107,462	21,053	90,967	7,021
hsa-miR-887	71,268	13,551	60,383	9,286
hsa-miR-92b-3p	67,689	6,917	57,383	4,240
hsa-miR-328	136,837	17,593	116,026	23,287
hsv1-miR-H3*	58,360	6,782	49,486	7,057
hsa-miR-4319	70,938	10,314	60,154	8,986

hsa-miR-602	74,806	7,775	63,495	6,925
hsa-miR-33b-3p	82,545	16,668	70,066	9,344
hsa-miR-29b-1-5p	88,007	7,359	74,767	10,889
hsa-miR-181c-3p	93,838	19,031	79,907	12,362
hsa-miR-1224-3p	111,387	10,456	94,917	21,374
hsa-miR-554	70,292	10,369	59,913	9,467
hsa-miR-200a-3p	66,529	8,368	56,759	7,346
hsa-miR-3647-5p_v17.0	150,966	21,972	128,890	27,060
hsa-miR-615-3p	124,901	12,610	106,649	16,429
hsa-miR-584-5p	78,836	4,055	67,316	13,679
hsa-miR-4269	65,139	8,308	55,630	6,503
hsa-miR-200b-3p	79,858	10,515	68,250	8,780
hsa-miR-33b-3p	82,778	10,141	70,804	8,595
hsa-miR-492	57,540	6,691	49,226	5,978
hsa-miR-1296	129,379	13,688	110,697	41,708
hsa-miR-155-3p	63,260	9,016	54,165	4,758
hsa-miR-3917	66,610	8,970	57,054	7,299
hsa-miR-613	55,566	6,307	47,650	3,821
hsa-miR-766-3p	153,308	23,506	131,551	19,566
hsa-miR-146b-3p	65,721	5,760	56,414	5,953
hsa-miR-29b-1-5p	100,627	9,445	86,467	7,989
hsa-miR-22-3p	10321,656	731,904	8878,383	600,706
hsa-miR-31-5p	66,606	7,636	57,323	7,889
hcmv-miR-US4	65,581	18,103	56,447	4,890
hsa-miR-18b-3p	62,164	3,352	53,517	6,191
hsa-miR-514b-5p	58,908	5,528	50,740	5,938
hsa-miR-874	114,530	13,118	98,698	8,893
hsa-miR-195-3p	83,883	8,355	72,341	10,047
hsa-miR-3940-3p	92,494	9,448	79,858	17,246
hsa-miR-3187-3p	47,907	7,206	41,452	4,095
hsa-miR-483-3p	160,135	17,684	138,597	31,715
hsv1-miR-H1*	220,521	19,083	190,873	79,574
hsa-miR-3610	64,420	12,979	55,765	9,534
hsv1-miR-H6-3p	242,570	25,490	210,319	72,146
hsa-miR-186-5p	454,544	38,337	394,381	20,381
hsa-let-7g-3p	59,883	5,900	51,959	5,298
ebv-miR-BART7	57,489	5,977	49,909	6,102
hsa-miR-625-3p	63,840	7,484	55,441	7,334
hsa-miR-449a	62,634	9,700	54,397	7,130
hsa-let-7d-3p	108,026	14,045	93,841	11,410
hcmv-miR-UL148D	65,517	7,323	56,924	8,924
hsa-miR-3126-3p	60,664	4,966	52,740	8,231
ebv-miR-BART2-5p	62,344	8,021	54,223	5,608
hsa-miR-933	102,748	9,953	89,444	10,943
hsa-miR-876-5p	82,600	7,833	71,911	23,080
hsa-miR-1538	61,460	7,795	53,518	6,670
hcmv-miR-US4	64,803	13,259	56,467	8,365

hsa-miR-1183	73,189	16,157	63,848	7,623
hsa-miR-3676-3p	77,526	8,242	67,655	5,856
hsa-miR-3682-3p	66,704	6,157	58,219	6,571
hsa-miR-615-3p	91,233	10,058	79,637	18,005
hsa-miR-605	121,748	11,450	106,312	27,676
hsa-miR-3663-5p	66,906	8,636	58,452	6,336
hcmv-miR-US5-1	52,430	4,002	45,808	4,133
hsa-miR-147b	48,904	7,343	42,728	4,053
hsa-miR-3917	67,074	8,620	58,644	6,260
hsa-miR-493-3p	53,448	6,267	46,736	5,220
hsa-let-7f-1-3p	98,882	10,958	86,499	13,187
hsa-miR-548am-5p	142,687	30,105	124,833	15,548
hsa-miR-943	55,794	3,820	48,818	5,180
hsa-miR-4297	75,319	8,703	65,903	7,229
hsa-miR-510	55,047	4,388	48,185	5,434
hsa-miR-1238	103,887	10,092	90,991	6,904
hsa-miR-3141	111,184	22,545	97,408	18,704
hsa-let-7b-3p	101,084	6,634	88,576	12,075
ebv-miR-BART7	64,969	5,609	56,965	6,156
hsa-miR-449c-3p	125,967	19,106	110,487	15,598
hsa-miR-621	50,943	5,503	44,686	5,377
hsa-miR-877-3p	101,323	10,108	88,928	21,309
hsa-let-7f-1-3p	103,201	7,357	90,578	11,881
hsa-miR-3132	82,741	8,821	72,665	7,522
hsa-miR-3178	44,662	4,894	39,237	4,245
hsa-miR-1307-3p	85,147	8,230	74,815	6,092
hsa-miR-943	56,733	6,678	49,869	7,707
hsa-miR-876-5p	83,558	4,979	73,482	16,699
hsa-miR-613	61,344	8,969	53,957	6,484
hsa-miR-548am-5p	128,051	27,888	112,658	12,250
hsa-miR-548b-5p	53,226	4,602	46,838	5,655
hsa-miR-139-5p	53,717	5,646	47,291	6,031
hsa-miR-937	121,802	10,417	107,235	26,706
hsa-miR-1470	62,115	7,362	54,690	5,622
hsa-miR-3647-5p_v17.0	148,640	19,080	130,899	23,292
hsa-miR-26a-5p	10381,833	989,584	9148,157	831,922
hsv1-miR-H8	77,216	13,299	68,143	7,064
hsa-miR-3180-3p	49,504	5,702	43,694	5,809
hsa-miR-636	63,175	7,792	55,765	8,636
hsa-miR-3615	50,076	5,743	44,241	6,872
hsa-miR-4323	80,009	6,951	70,695	9,992
hsa-miR-4314	64,079	9,831	56,643	6,299
hsa-miR-200c-5p	61,834	10,885	54,679	7,049
hcmv-miR-US25-1	51,130	8,053	45,231	5,019
hsa-let-7d-3p	84,187	11,414	74,502	9,290
hsa-miR-1976	58,353	7,454	51,654	6,148
hsa-let-7g-3p	60,823	5,682	53,848	7,299

hsa-miR-4290	205,230	17,923	181,714	47,776
hsa-miR-3622b-3p	60,137	8,355	53,280	4,552
hsa-miR-93-3p	53,639	7,952	47,546	5,944
hsa-miR-1233	51,190	4,163	45,384	6,438
hsa-miR-3714	73,841	9,922	65,484	7,163
hsa-miR-498	56,046	7,390	49,706	5,197
hsa-miR-549	47,560	4,205	42,219	6,906
hsa-miR-144-5p	52,106	9,411	46,257	6,654
hsa-miR-486-5p	55,349	6,816	49,164	5,142
hsa-miR-3677-3p	45,983	5,500	40,849	5,154
hcmv-miR-US25-1	48,924	6,667	43,464	6,111
hsa-miR-619	51,752	6,484	45,981	5,572
hsa-miR-149-3p	50,117	6,651	44,562	5,449
hsa-miR-1237	73,404	7,171	65,274	7,027
hsa-miR-1470	66,668	9,813	59,301	6,597
hsa-miR-365a-3p	1143,981	257,435	1017,834	55,167
hsa-miR-149-5p	114,240	9,563	101,659	24,010
hsa-miR-4277	52,732	5,816	46,943	6,793
hsv2-miR-H20	58,994	6,231	52,530	5,144
hsa-let-7f-1-3p	107,459	7,262	95,701	9,836
hsa-miR-1234	120,335	7,332	107,267	17,271
hsa-miR-133a	56,651	4,781	50,503	5,316
hsa-miR-566	65,967	4,210	58,826	10,287
hcmv-miR-US33-3p	76,994	8,735	68,674	14,802
hsa-miR-640	75,554	8,906	67,394	10,550
hsa-miR-3124-5p	64,787	5,746	57,797	13,398
hsa-miR-3194-5p	76,091	14,548	67,897	6,878
hsa-miR-3676-3p	75,988	6,141	67,812	7,500
hsa-miR-346	54,716	5,554	48,834	6,235
hsa-miR-1229	75,316	7,750	67,219	6,351
hsa-miR-1183	71,984	11,572	64,299	10,768
ebv-miR-BART11-3p	47,160	5,028	42,160	6,012
hsa-miR-567	52,215	4,363	46,706	6,642
hsa-miR-1915-5p	46,214	5,428	41,358	5,029
hsa-miR-675-5p	41,048	5,416	36,740	5,516
hsa-miR-130a-3p	172,613	15,787	154,509	27,691
hsa-miR-548d-3p	50,240	6,115	45,008	4,442
ebv-miR-BHRF1-1	47,557	5,477	42,615	6,165
hsa-miR-33a-3p	77,712	5,656	69,640	16,429
hsa-miR-1911-3p	53,864	7,053	48,272	7,035
hsa-miR-381	87,134	8,631	78,101	9,389
hsa-miR-3679-3p	66,439	6,544	59,564	6,397
kshv-miR-K12-12*	57,661	6,640	51,698	8,605
hsa-miR-4262	49,214	4,883	44,180	7,366
hsa-miR-485-3p	158,652	17,226	142,460	24,216
hsa-miR-873-5p	51,110	4,729	45,894	4,779
hsa-miR-887	65,715	8,241	59,013	8,638

hsa-let-7f-1-3p	104,911	12,092	94,222	11,976
hsa-miR-641	56,432	6,157	50,692	7,421
hsa-miR-3180-5p	62,428	5,553	56,085	5,580
hsa-miR-218-2-3p	43,074	6,245	38,707	4,899
hsa-miR-3141	123,726	28,548	111,185	22,067
hsa-miR-1972	56,933	6,942	51,171	5,686
hsa-miR-520e	53,792	7,578	48,352	4,616
hsa-miR-1284	48,973	6,258	44,026	6,807
hsa-miR-635	46,013	5,902	41,385	3,560
hsa-miR-299-5p	69,481	6,476	62,499	9,097
hsa-let-7d-3p	77,791	8,468	69,980	7,310
hsa-miR-3182	49,853	5,872	44,849	6,461
hsv2-miR-H20	59,853	4,985	53,848	4,259
hsa-miR-182-5p	75,622	10,189	68,051	23,064
ebv-miR-BART1-5p	54,089	4,584	48,699	6,823
hsa-miR-135b-3p	40,248	6,109	36,258	3,196
hsv2-miR-H2	55,918	6,118	50,385	7,540
hsv2-miR-H6*	332,259	24,449	299,419	118,483
hsa-miR-486-5p	59,259	6,740	53,446	7,209
hsa-miR-890	41,341	5,023	37,300	3,576
hsa-miR-648	47,167	4,736	42,587	6,177
hsv1-miR-H8*	47,414	3,351	42,815	6,642
ebv-miR-BART17-3p	68,657	7,058	62,032	7,625
hsa-miR-641	52,135	5,376	47,115	6,988
hsa-miR-615-3p	80,375	8,475	72,645	19,010
hsa-miR-3622a-3p	63,001	8,392	56,949	6,697
hsa-miR-296-3p	50,863	3,916	45,990	5,715
hsa-miR-346	62,659	5,968	56,658	6,320
kshv-miR-K12-1*	68,452	7,821	61,903	8,266
ebv-miR-BART3*	97,849	14,528	88,510	11,090
hsa-miR-371a-5p	70,809	6,893	64,084	11,807
hsa-miR-1976	61,919	7,188	56,039	6,202
hsa-miR-138-1-3p	44,414	3,972	40,196	4,252
hsa-miR-4323	92,535	7,852	83,757	9,095
hsa-miR-4305	54,031	6,121	48,920	7,254
hcmv-miR-US33-3p	74,397	7,841	67,361	17,388
hsa-miR-138-1-3p	46,730	4,309	42,316	5,343
ebv-miR-BART10	81,417	10,446	73,742	7,397
hsv1-miR-H6-5p	85,002	20,182	77,016	9,857
hsa-miR-148a-5p	70,312	6,692	63,711	17,792
hsa-miR-593-3p	47,222	6,982	42,807	4,505
hsa-miR-4305	56,692	7,171	51,423	8,488
hsa-miR-605	106,070	8,572	96,216	20,690
hsa-miR-615-3p	96,376	7,520	87,459	18,225
hsa-miR-3677-3p	47,606	6,828	43,211	5,596
hsa-miR-550a-3p	82,793	5,830	75,187	9,535
hsa-miR-1280	1302,822	104,073	1183,834	253,855

hsa-miR-194-3p	56,625	4,743	51,457	6,798
hcmv-miR-US33-3p	77,125	7,612	70,105	19,720
hsa-miR-92b-3p	63,877	7,129	58,088	7,832
hsa-miR-214-3p	48,455	6,529	44,081	4,386
hsa-miR-20b-3p	45,720	5,363	41,594	5,252
hcmv-miR-US25-1	48,414	5,194	44,074	3,370
hsa-miR-33a-3p	77,220	7,754	70,308	17,633
hsa-miR-425-3p	76,456	8,072	69,622	7,149
hsa-miR-4253	63,123	6,701	57,481	7,121
hsa-miR-1238	88,767	7,862	80,840	8,657
hsa-miR-597	49,731	7,112	45,293	4,769
hsa-miR-33a-3p	63,067	8,124	57,445	12,317
hsa-miR-943	50,439	6,525	45,944	6,058
hsa-miR-567	48,700	6,068	44,361	5,883
ebv-miR-BART2-5p	59,248	5,864	53,976	4,900
hsa-miR-3180-5p	60,155	3,930	54,804	4,968
hsa-miR-548aa	75,711	6,734	68,988	13,244
hsv2-miR-H20	62,407	6,064	56,866	5,769
hsa-miR-18b-3p	55,984	5,001	51,014	5,712
hsa-miR-668	51,523	8,955	46,980	5,129
hcmv-miR-US33-5p	54,991	6,630	50,160	8,615
hsv1-miR-H1	81,669	6,853	74,516	10,295
hsa-miR-423-3p	52,278	5,780	47,706	4,045
hsv1-miR-H15	63,087	8,866	57,580	6,750
hsa-miR-596	42,802	4,682	39,096	2,746
hsa-miR-105-5p	45,658	3,940	41,705	2,887
hsa-miR-3622b-3p	54,158	8,048	49,486	6,897
hsa-miR-1276	50,887	5,454	46,506	7,280
hsa-miR-3185	51,124	5,930	46,725	6,787
hsa-miR-196a-3p	44,859	5,627	40,999	5,981
hsa-miR-513a-3p	45,353	6,867	41,455	5,333
hsa-miR-657	54,108	6,798	49,480	7,104
hsa-miR-516a-3p	46,541	4,867	42,561	4,223
hsa-miR-505-5p	115,085	14,298	105,270	12,138
hsa-miR-16-1-3p	49,504	4,989	45,309	6,128
hsa-miR-302a-5p	47,392	6,809	43,378	7,019
hsa-miR-4296	49,700	6,392	45,497	5,631
hsa-miR-1229	74,293	5,628	68,014	8,771
hcmv-miR-US5-1	41,479	3,366	37,982	3,076
hsa-miR-4321	47,107	5,675	43,139	5,053
hsa-miR-624-3p	53,858	5,888	49,324	6,291
hsa-miR-602	71,227	5,763	65,233	6,818
hsa-miR-4294	52,866	5,975	48,421	7,023
hsa-miR-182-3p	45,894	6,632	42,038	4,224
hsa-miR-3667-5p	76,562	21,556	70,136	13,232
hsa-miR-760	66,583	8,383	61,005	6,914
hsa-miR-548h-3p	51,275	5,602	46,985	5,513

hsa-miR-583	52,280	6,676	47,935	4,427
hsa-miR-933	79,882	5,078	73,244	9,710
hsa-miR-4262	51,681	6,096	47,410	6,855
hsa-miR-299-5p	69,170	5,967	63,480	10,234
hsa-miR-101-5p	63,654	8,675	58,456	3,775
hsa-miR-708-5p	48,848	8,027	44,871	4,461
hsa-miR-3622a-3p	60,574	7,458	55,643	7,612
kshv-miR-K12-8*	66,171	7,258	60,796	6,705
hcmv-miR-US5-2	41,850	4,098	38,453	3,526
hsa-miR-3178	43,056	6,366	39,562	5,179
hsa-miR-585	46,683	3,691	42,897	6,610
hsa-miR-1200	49,098	5,230	45,121	5,697
hsa-miR-3615	43,857	7,103	40,305	3,720
hsa-miR-1539	82,273	7,787	75,640	8,741
hsa-miR-550a-3p	71,068	7,154	65,340	4,432
hsa-miR-3146	47,278	4,426	43,469	6,259
hsa-miR-149-3p	53,245	9,158	48,961	7,002
hsa-miR-148a-5p	65,641	9,172	60,364	17,793
hsv2-miR-H2	51,295	4,886	47,174	4,769
hsa-miR-196b-3p	56,876	5,736	52,313	6,190
ebv-miR-BHRF1-1	54,872	4,847	50,471	5,545
hsa-miR-1224-3p	84,906	10,286	78,097	17,300
hsa-miR-92b-3p	65,019	8,592	59,808	5,749
hsa-miR-432-3p	62,884	4,869	57,850	9,265
hsa-miR-146a-3p	54,556	5,841	50,200	5,087
hsa-miR-3181	42,815	3,889	39,397	4,708
hsa-miR-891a	49,638	5,497	45,684	5,889
hsv2-miR-H22	96,070	19,385	88,431	26,182
ebv-miR-BHRF1-2	41,075	5,358	37,811	4,106
hsa-miR-485-3p	178,752	24,486	164,599	23,648
hsa-miR-640	77,165	6,562	71,118	11,198
hcmv-miR-US25-2-3p	41,178	5,359	37,956	4,346
hsa-miR-22-3p	5150,044	143,817	4748,311	189,383
hcmv-miR-UL22A*	44,838	5,702	41,344	5,768
hsa-miR-182-5p	66,840	6,771	61,635	18,558
hsa-miR-33b-3p	79,523	10,277	73,347	7,717
hsa-miR-139-3p	50,665	6,370	46,739	3,959
hsa-miR-144-5p	46,673	3,843	43,063	5,697
hsa-miR-613	58,284	7,250	53,779	7,509
hsa-miR-4279	50,521	5,256	46,643	5,547
kshv-miR-K12-8*	66,629	7,502	61,516	8,015
hsa-miR-922	41,674	6,339	38,478	5,358
hsa-miR-296-3p	44,284	5,411	40,889	6,929
hsa-miR-647	79,181	8,377	73,125	19,667
hsa-miR-4263	46,104	4,910	42,593	3,221
hsa-miR-936	55,273	5,498	51,070	7,606
ebv-miR-BART2-5p	59,786	8,176	55,240	6,834

hsa-miR-1253	51,290	5,683	47,412	6,361
hsa-miR-548v	73,253	9,431	67,717	9,639
hsa-miR-99b-3p	53,991	5,565	49,918	4,949
hsa-miR-409-5p	44,024	5,105	40,704	3,102
hsa-miR-634	64,198	5,701	59,380	4,615
hsa-miR-4277	51,075	5,025	47,244	7,567
hcmv-miR-UL112	45,209	5,021	41,822	4,581
hsa-miR-623	62,039	8,870	57,418	3,228
hcmv-miR-UL148D	59,552	6,725	55,120	5,491
hsa-miR-563	62,809	7,978	58,137	8,169
hsa-miR-561-3p	47,248	7,623	43,737	4,698
hsa-miR-1911-3p	55,180	4,160	51,095	6,519
hsa-miR-943	52,817	6,639	48,908	6,809
hsa-miR-4258	55,909	7,953	51,772	8,480
hsa-miR-194-3p	46,582	2,005	43,136	3,996
hsa-miR-520b	55,085	8,029	51,015	7,238
hsa-miR-1229	61,467	7,389	56,927	5,926
hsa-miR-138-1-3p	43,961	5,613	40,728	6,803
hsa-miR-133b	54,475	5,339	50,471	7,072
ebv-miR-BHRF1-1	60,190	5,202	55,770	3,928
hsa-miR-381	84,872	9,716	78,647	8,272
hsa-miR-4263	45,862	5,112	42,499	5,404
hsv2-miR-H22	91,539	18,986	84,830	25,518
ebv-miR-BART9*	42,608	5,500	39,494	3,526
hsa-miR-597	44,499	6,316	41,249	4,558
hsa-miR-1264	50,769	4,376	47,079	5,448
hsa-miR-767-3p	50,561	3,992	46,886	4,960
ebv-miR-BART7	59,870	6,474	55,523	5,184
hsa-miR-181a-2-3p	47,161	5,237	43,739	4,830
hsa-miR-4279	50,270	5,747	46,623	5,349
hsa-miR-1203	51,734	5,776	47,991	6,169
hsa-miR-3621	53,267	6,218	49,416	5,911
hsa-miR-3605-5p	60,704	4,965	56,328	8,842
hsa-miR-520e	53,614	5,589	49,777	5,364
hsa-miR-518a-5p	50,108	5,144	46,529	5,869
hsa-miR-16-2-3p	53,488	6,091	49,671	7,611
hsa-miR-29c-5p	219,920	41,291	204,237	16,948
hsa-miR-185-3p	49,485	3,783	45,976	3,040
hsa-miR-3714	58,448	6,420	54,303	6,700
hsa-miR-616-3p	47,658	6,308	44,279	5,333
hsa-miR-636	57,047	5,064	53,015	4,709
hsa-miR-200c-5p	53,538	7,987	49,756	5,960
hsa-miR-541-5p	63,869	7,160	59,367	15,630
hsa-miR-4258	50,536	7,109	46,989	3,134
hsa-miR-217	42,217	7,136	39,264	2,895
hsa-miR-92b-5p	44,270	7,032	41,174	6,432
hsa-miR-3689a-5p	47,043	5,102	43,760	4,764

hsa-miR-4253	60,536	5,429	56,312	6,519
hbv-miR-B4	53,678	7,002	49,949	7,278
hsa-miR-1280	236,738	25,575	220,304	27,692
ebv-miR-BART5*	50,223	6,166	46,745	6,406
hsa-miR-101-5p	72,591	9,623	67,566	5,057
hsa-miR-548b-5p	50,382	6,581	46,903	4,127
hsa-miR-642a-5p	48,416	5,341	45,082	6,434
hsa-miR-4288	45,754	5,097	42,606	6,059
hsa-miR-4283	43,913	4,038	40,899	5,713
ebv-miR-BART11-3p	52,130	6,100	48,553	7,439
hsa-miR-3667-5p	80,640	15,429	75,111	9,277
hsv1-miR-H7*	86,387	7,116	80,466	7,745
hsa-miR-1322	56,082	5,686	52,239	5,301
hsa-miR-3926	94,823	8,736	88,356	7,558
hsa-miR-3910	44,774	4,433	41,722	5,790
hsa-miR-504	50,255	4,299	46,834	2,956
hsa-miR-1238	84,907	5,201	79,145	6,568
hsa-miR-635	48,673	6,584	45,373	6,094
ebv-miR-BART1-3p	53,997	4,951	50,340	3,714
hsa-miR-412	44,465	4,060	41,475	5,970
hsa-miR-302b-5p	45,960	4,679	42,871	5,856
hsa-miR-3687	44,601	4,909	41,608	4,818
hsa-miR-875-3p	46,846	3,884	43,715	8,692
hsa-miR-224-3p	46,940	5,481	43,805	4,637
ebv-miR-BART17-3p	57,148	5,012	53,342	6,937
hsa-miR-3621	46,883	5,416	43,765	5,385
hsa-miR-600	43,884	4,274	40,971	4,168
hsa-miR-130a-5p	60,619	4,904	56,599	8,443
hsv1-miR-H16	74,774	10,896	69,820	9,055
hsa-miR-3181	42,867	4,764	40,027	6,030
hsa-miR-548v	73,521	6,243	68,653	11,151
hsa-miR-3614-3p	46,249	4,374	43,189	5,362
hsa-miR-1226-3p	56,197	7,755	52,479	5,006
hsa-miR-412	43,114	5,355	40,266	4,722
hsa-miR-588	52,679	7,969	49,200	5,629
hsa-miR-520f	68,636	9,337	64,119	8,383
hsa-miR-569	43,687	5,976	40,822	4,847
hsa-miR-593-5p	45,358	5,377	42,385	4,272
hsa-miR-668	48,522	6,888	45,358	5,248
hsa-miR-671-3p	57,635	4,968	53,882	6,180
hsa-miR-3180-3p	49,096	5,729	45,909	6,300
hsa-miR-188-5p	159,807	73,950	149,448	40,889
hsa-miR-625-5p	83,520	13,969	78,109	6,329
hsa-miR-3944-3p	48,554	5,335	45,417	5,862
hsa-miR-4308	53,011	6,292	49,590	7,603
hsa-miR-892a	48,503	5,382	45,387	4,016
hsa-miR-1238	90,393	8,780	84,586	9,472

hsa-miR-3159	49,393	5,686	46,221	7,346
hsa-miR-634	59,235	5,908	55,436	6,341
hsa-miR-493-3p	49,040	7,434	45,899	5,090
hsa-miR-1237	70,198	7,071	65,703	7,438
hsv1-miR-H4-5p	50,229	5,203	47,015	6,706
hsa-miR-1278	59,237	7,136	55,450	6,849
ebv-miR-BHRF1-1	48,549	5,702	45,447	6,290
hsa-miR-200c-5p	53,172	5,685	49,780	4,292
hsa-miR-3622a-5p	48,174	6,655	45,108	4,167
hsa-miR-1182	51,746	5,756	48,453	6,279
hsv1-miR-H4-3p	46,584	5,859	43,620	4,889
hsa-miR-423-3p	52,169	4,127	48,852	6,773
hsa-miR-563	63,646	5,204	59,602	6,769
hsa-miR-555	45,531	7,559	42,643	4,212
hsa-miR-3678-5p	48,548	5,832	45,475	4,423
kshv-miR-K12-9*	56,274	4,010	52,722	5,764
hsa-miR-1264	53,251	4,845	49,893	4,791
ebv-miR-BART14*	44,774	3,774	41,952	5,347
hcmv-miR-US33-5p	54,161	6,957	50,747	6,811
ebv-miR-BART9*	50,356	8,360	47,195	5,644
hsa-miR-1307-3p	49,793	5,842	46,681	5,467
ebv-miR-BART14*	45,284	5,291	42,461	3,402
hsv1-miR-H4-5p	49,850	6,716	46,743	5,993
hsa-miR-330-5p	46,573	4,624	43,672	4,403
ebv-miR-BART17-5p	41,499	3,720	38,942	5,011
hsa-miR-29a-5p	61,660	6,386	57,866	4,924
hbv-miR-B2RC	57,856	9,362	54,304	6,795
hsa-miR-1284	52,734	2,925	49,498	6,048
kshv-miR-K12-3*	42,458	5,649	39,853	3,698
hsa-miR-149-5p	100,222	10,076	94,086	16,953
hsa-miR-621	48,335	7,875	45,380	8,136
hsa-miR-1182	52,749	5,275	49,528	6,902
hsa-miR-193b-5p	54,256	7,636	50,948	7,066
hsa-miR-124-5p	43,848	5,864	41,176	5,735
hsa-miR-4318	48,312	5,687	45,377	6,104
hsa-miR-525-3p	46,259	4,462	43,451	4,468
hsa-miR-3184-5p	45,736	4,537	42,965	4,926
ebv-miR-BART20-3p	47,002	5,318	44,160	5,682
hsa-miR-130a-3p	143,249	12,190	134,595	23,866
hsa-miR-3690	45,119	4,950	42,394	6,188
hsa-miR-1227	68,457	5,320	64,328	9,561
kshv-miR-K12-9*	52,139	4,617	48,997	5,376
hsa-miR-556-5p	42,274	4,902	39,728	4,920
hsa-miR-125b-1-3p	47,129	4,456	44,294	5,663
hsa-miR-643	46,033	5,394	43,268	6,258
hsa-miR-99b-3p	54,032	4,065	50,787	7,852
hsa-miR-548h-5p	45,180	4,802	42,473	5,334

hsa-miR-200b-3p	57,753	5,519	54,293	7,415
hsa-miR-767-3p	52,488	5,158	49,349	6,867
hsa-miR-637	48,694	3,135	45,782	4,307
hsa-miR-182-3p	47,819	3,794	44,962	6,666
hsa-miR-33b-3p	74,546	7,515	70,098	6,533
hsa-miR-2052	45,210	5,905	42,512	3,737
hsa-miR-15a-5p	11312,151	403,547	10639,067	606,959
ebv-miR-BART3*	89,307	10,590	84,025	12,559
hsa-miR-147a	48,407	5,436	45,561	6,478
hsa-miR-1297	44,067	6,156	41,481	5,195
hsa-miR-601	67,658	8,429	63,693	8,230
hsa-let-7e-3p	52,718	5,440	49,635	5,368
hsa-miR-589-5p	44,498	5,168	41,896	3,740
hsa-miR-583	52,745	6,657	49,661	6,772
hsa-let-7e-3p	56,510	6,463	53,213	7,348
kshv-miR-K12-2	40,754	3,606	38,382	5,424
hsa-miR-576-3p	47,859	8,860	45,088	6,198
kshv-miR-K12-3*	40,091	5,735	37,772	3,553
hsa-miR-3182	51,463	6,608	48,490	5,939
hsa-miR-518a-5p	48,724	5,078	45,919	4,941
hsa-miR-520a-3p	49,724	5,347	46,862	6,163
hsa-miR-623	63,166	5,839	59,538	8,063
hcmv-miR-US25-1*	43,944	5,323	41,424	5,395
hsa-miR-183-3p	43,426	5,062	40,941	4,306
hsa-miR-520f	68,784	9,832	64,854	11,327
hsa-miR-504	49,088	6,609	46,283	3,119
hsa-miR-138-5p	45,615	6,849	43,013	5,825
hsa-miR-661	45,553	5,274	42,955	6,887
ebv-miR-BART18-5p	49,984	3,197	47,135	3,493
hsa-miR-4311	58,148	3,533	54,848	6,159
hsa-miR-676-5p	44,667	5,653	42,133	5,744
hsa-miR-16-1-3p	47,793	5,096	45,089	5,413
kshv-miR-K12-4-3p	42,201	5,543	39,830	5,779
hsa-miR-33a-3p	82,431	5,925	77,801	18,700
hsv1-miR-H5-3p	50,351	5,651	47,533	6,312
hsa-miR-1247-5p	44,423	6,057	41,941	3,410
hsa-miR-183-3p	40,731	5,446	38,459	6,513
hsa-miR-130a-5p	66,528	4,810	62,821	8,105
hsa-miR-217	40,969	4,854	38,686	3,758
hsa-miR-19b-2-5p	48,721	5,941	46,010	5,378
hsa-miR-302c-5p	46,351	5,273	43,775	5,765
hsa-miR-92a-2-5p	46,360	3,430	43,793	5,234
hsa-miR-509-3p	44,075	4,979	41,638	4,315
kshv-miR-K12-6-5p	45,701	5,106	43,178	2,200
hsa-miR-196b-3p	57,370	6,452	54,219	7,256
hsa-miR-3682-3p	67,638	6,247	63,928	7,051
hsa-miR-187-5p	44,820	5,234	42,364	2,570

hsa-miR-550a-3p	73,146	5,819	69,148	5,808
hbv-miR-B4	52,597	6,465	49,734	4,569
hsa-miR-146a-3p	49,297	3,602	46,615	5,551
hsa-miR-3120-3p	50,043	4,455	47,324	4,331
hsa-miR-200a-3p	52,695	5,901	49,833	5,760
hsa-miR-3678-3p	44,196	5,050	41,808	3,814
hsa-miR-3153	45,544	5,202	43,086	5,002
hsa-miR-3926	87,710	10,687	82,987	9,206
hsa-miR-4326	70,354	5,622	66,569	10,590
hsa-miR-600	41,953	5,909	39,696	4,095
hsa-miR-550a-5p	66,610	6,679	63,036	5,644
hsa-miR-4328	46,246	5,690	43,765	5,074
hsv2-miR-H7-3p	59,026	6,289	55,862	5,899
hsa-miR-4293	47,470	6,065	44,926	5,246
hsa-miR-16-1-3p	46,751	4,380	44,251	4,753
hsa-miR-1253	49,401	6,180	46,760	6,512
hsa-miR-581	45,289	5,496	42,867	5,681
hsa-miR-302d-3p	48,613	5,428	46,015	5,362
hsa-miR-19a-5p	47,458	5,326	44,932	6,183
hsa-miR-31-5p	56,340	6,514	53,346	5,967
hsa-miR-3144-3p	48,360	5,369	45,795	6,375
hsa-miR-1303	55,421	6,146	52,482	5,477
hsa-miR-124-3p	44,111	7,338	41,772	6,869
hsa-miR-885-3p	44,762	4,931	42,389	4,982
hsa-miR-25-5p	42,172	4,789	39,937	4,650
hsa-miR-1226-3p	49,578	5,828	46,956	5,103
hsa-miR-873-5p	50,668	3,470	47,989	4,624
hsa-miR-432-3p	61,692	5,339	58,432	6,320
hsa-miR-452-3p	41,369	3,481	39,204	4,877
hsa-miR-1257	48,321	7,145	45,796	5,506
hsa-miR-222-5p	43,725	4,864	41,446	4,828
hsa-miR-635	48,218	5,021	45,712	5,982
hsa-miR-4320	49,435	3,073	46,867	5,689
ebv-miR-BART8	46,645	4,788	44,224	7,224
hsa-miR-3675-3p	64,607	8,110	61,257	8,035
hsa-miR-619	50,032	4,630	47,437	5,062
hsa-miR-1236	73,518	9,081	69,709	11,452
hsa-miR-194-3p	48,211	4,773	45,715	5,807
hsa-miR-665	54,737	6,210	51,937	6,751
hsa-miR-581	46,547	5,015	44,173	4,807
hsa-miR-654-5p	62,246	10,614	59,074	8,829
hsa-miR-4329	50,841	4,620	48,252	6,210
hsa-miR-1203	52,835	5,592	50,152	5,143
hsa-miR-1539	79,227	7,792	75,205	7,166
hsa-miR-27b-5p	52,998	7,140	50,313	7,409
hsa-miR-582-3p	51,144	8,145	48,558	7,610
hsa-miR-181c-3p	69,813	12,747	66,299	9,352

hsa-miR-583	45,975	6,407	43,661	5,406
hsa-miR-621	50,189	5,723	47,663	7,398
hsa-miR-548t-5p	51,700	5,193	49,102	5,441
hsa-miR-18a-3p	49,281	5,030	46,805	2,596
hsa-miR-18a-3p	47,997	4,770	45,591	5,363
kshv-miR-K12-6-5p	46,054	4,695	43,745	3,352
ebv-miR-BHRF1-2	39,064	4,992	37,118	4,678
hsa-miR-890	42,480	4,900	40,368	5,532
hsa-miR-1227	79,037	6,718	75,109	12,333
hsa-miR-632	49,850	4,045	47,376	5,202
hsa-miR-760	56,411	5,506	53,612	5,943
hsa-miR-154-5p	51,238	5,861	48,697	6,239
hsa-miR-1538	61,695	5,499	58,650	9,410
hsa-miR-204-5p	62,955	6,852	59,852	6,709
hsa-miR-1260b	4504,935	157,602	4283,023	1121,252
hsa-miR-455-3p	47,345	5,526	45,013	5,082
hsa-miR-646	41,347	6,135	39,315	3,389
hsa-miR-216a	43,675	6,418	41,534	3,721
hsa-miR-4282	46,486	5,724	44,207	5,953
hsa-miR-3617	45,972	4,150	43,723	4,527
hsa-miR-99b-3p	58,661	4,818	55,796	7,190
hsa-miR-409-3p	51,840	4,482	49,323	6,053
hsa-miR-519b-3p	47,267	5,350	44,977	6,102
ebv-miR-BART2-3p	40,965	5,208	38,981	3,722
hsa-miR-541-3p	46,037	4,990	43,811	5,552
hsa-miR-662	61,012	5,828	58,078	6,129
hsa-miR-24-2-5p	40,309	4,517	38,374	4,391
hsa-miR-654-5p	50,504	5,690	48,084	5,046
ebv-miR-BART11-3p	46,026	5,214	43,825	5,484
hsa-miR-634	64,629	5,318	61,557	8,892
hcmv-miR-UL148D	48,259	5,259	45,966	6,676
hsa-miR-523-3p	43,632	4,120	41,574	6,282
hsa-miR-106b-3p	42,801	5,845	40,782	5,921
hsa-miR-2277-5p	45,312	6,470	43,180	6,200
hsa-miR-4273	46,790	6,357	44,593	5,669
hsa-miR-619	50,487	8,331	48,116	5,632
hsa-miR-20b-3p	47,412	6,449	45,190	6,264
hsa-miR-16-5p	14641,762	664,936	13955,602	804,950
ebv-miR-BART13	160,615	18,896	153,118	11,348
hsa-miR-191-3p	78,227	8,278	74,580	6,653
hcmv-miR-UL70-5p	42,939	3,764	40,938	2,953
hsa-miR-16-5p	27696,645	3001,550	26406,567	3771,427
hsa-miR-379-3p	45,652	5,223	43,531	5,130
hsa-miR-3128	42,922	4,975	40,930	4,639
hsa-miR-576-5p	50,561	4,243	48,228	6,454
hsa-miR-130a-5p	62,901	7,730	60,010	9,106
hsa-miR-3166	45,860	5,629	43,755	5,623

hsa-miR-21-5p	187646,038	18553,818	179036,228	26747,265
hsa-miR-433	46,088	4,971	43,974	4,439
kshv-miR-K12-4-3p	43,580	6,371	41,597	4,767
hsa-miR-760	65,870	8,605	62,881	7,127
hsa-miR-409-3p	52,858	5,694	50,463	5,250
hsa-miR-3690	53,252	6,135	50,841	7,417
hsa-miR-367-5p	47,845	7,106	45,680	6,537
hsa-miR-517a-3p	47,581	5,150	45,428	4,981
hsa-miR-4268	43,393	5,249	41,436	4,870
hsa-miR-3911	80,321	8,004	76,709	7,101
hsa-miR-190b	52,585	4,783	50,232	6,338
hsa-miR-1200	48,159	5,713	46,007	6,428
hsa-miR-365b-5p	47,089	6,881	44,994	6,321
hsa-miR-1251	43,484	6,278	41,550	5,267
ebv-miR-BHRF1-2*	44,024	4,898	42,068	4,271
hsa-miR-1256	46,144	5,271	44,095	5,754
hsa-miR-99a-3p	46,312	5,755	44,261	5,941
ebv-miR-BART17-3p	49,945	6,578	47,735	5,993
hsa-miR-3675-3p	64,967	4,720	62,094	6,993
hcmv-miR-US4	60,606	7,422	57,933	6,942
hsa-miR-556-5p	45,627	5,218	43,617	4,608
hsa-miR-601	72,324	10,645	69,138	9,472
hsv1-miR-H6-5p	67,528	15,609	64,558	9,594
ebv-miR-BART5	50,227	4,265	48,018	4,724
hsa-miR-2115-5p	45,112	3,516	43,135	4,598
hsa-miR-640	70,120	7,910	67,056	9,062
ebv-miR-BART5	44,676	3,972	42,727	4,585
hsa-miR-3647-5p_v17.0	135,981	13,587	130,049	21,041
hsa-miR-200a-5p	47,504	5,129	45,433	5,617
bkv-miR-B1-5p	44,878	4,860	42,926	4,918
hsa-miR-30c-1-3p	50,605	5,766	48,404	4,953
hsa-miR-935	45,816	5,167	43,826	4,072
hsa-miR-3924	51,063	3,423	48,851	3,638
hsa-miR-3152-3p	42,685	5,390	40,838	4,772
hsa-miR-525-5p	48,916	5,373	46,806	3,764
hsa-miR-148a-5p	70,678	4,556	67,631	20,422
hsa-miR-518a-3p	49,452	5,001	47,321	5,847
hsa-miR-380-3p	48,549	5,203	46,458	5,468
hsa-miR-504	47,105	5,442	45,077	5,868
ebv-miR-BART19-5p	39,663	6,174	37,956	6,760
hsa-miR-551b-5p	41,203	4,562	39,430	4,535
hsa-miR-4310	67,677	5,368	64,767	6,832
hsa-miR-1322	49,724	5,975	47,593	5,663
hcmv-miR-US25-2-3p	42,507	5,391	40,690	5,879
hsa-miR-921	45,338	5,467	43,409	5,951
hsa-miR-936	48,881	5,502	46,806	5,083
hsa-miR-205-5p	47,205	6,221	45,208	6,272

hsa-miR-4330	44,439	4,965	42,562	5,512
hsa-miR-3134	43,899	5,745	42,046	5,516
hsa-miR-196a-3p	45,410	3,527	43,494	4,865
hcmv-miR-UL36	48,556	4,615	46,516	4,642
ebv-miR-BART20-3p	45,907	4,653	43,979	4,861
hsa-miR-3135a	42,283	4,836	40,507	4,870
hsa-miR-218-2-3p	45,227	6,352	43,333	5,038
hsv1-miR-H13	45,025	4,271	43,140	6,111
hcmv-miR-UL36*	44,280	4,410	42,435	6,342
hsa-miR-196b-5p	57,951	5,413	55,541	6,374
ebv-miR-BART18-3p	46,083	6,334	44,167	4,286
hsa-miR-3684	47,934	4,787	45,941	6,228
hsa-miR-548b-5p	51,199	6,073	49,081	5,761
hsa-miR-376a-3p	45,285	5,518	43,414	5,938
hsa-miR-33b-5p	46,329	7,264	44,417	5,523
hsa-miR-623	65,626	7,636	62,919	5,325
hsa-miR-4276	45,799	4,410	43,924	5,354
hsa-miR-1233	46,943	5,494	45,023	5,379
hsa-miR-105-5p	47,209	4,081	45,283	5,396
hsa-miR-618	47,074	5,947	45,158	4,240
hsa-miR-675-3p	62,580	7,232	60,033	8,468
hsa-miR-559	45,463	4,903	43,613	3,729
hsa-miR-490-3p	45,164	6,377	43,328	5,986
hsa-miR-582-3p	53,326	6,464	51,161	7,909
hsa-miR-3688-3p	48,894	8,050	46,909	5,357
hsa-miR-1303	55,690	7,560	53,441	7,097
hsa-miR-4264	45,297	4,366	43,472	6,939
hsa-miR-146b-3p	41,222	5,400	39,565	6,171
hsa-miR-4320	50,874	6,079	48,831	5,699
hsa-miR-1256	49,274	5,476	47,299	5,806
hsa-miR-1253	48,949	5,849	46,989	6,401
hsa-miR-3155a	47,463	5,658	45,563	5,520
hsa-miR-1202	919,092	87,210	882,521	122,238
hsa-miR-3650	50,263	4,962	48,264	4,616
hsa-miR-760	57,983	9,491	55,684	8,204
hsa-miR-3142	43,399	5,317	41,679	5,106
hsa-miR-4293	46,184	5,603	44,357	6,074
hsa-miR-3183	40,190	5,012	38,601	4,809
hsa-miR-769-3p	53,247	4,995	51,151	5,849
hsa-miR-92b-5p	44,834	4,182	43,069	4,673
hsa-miR-1284	50,374	6,047	48,391	6,261
hsa-miR-519d	53,909	4,196	51,794	6,188
hsa-miR-218-1-3p	38,410	5,597	36,904	5,301
hsa-miR-3126-3p	55,646	6,312	53,468	8,966
hsa-miR-548e	58,635	7,222	56,343	6,951
hsa-miR-1250	49,992	5,327	48,044	5,538
kshv-miR-K12-1	41,398	4,545	39,787	5,528

hsa-miR-18b-3p	58,666	5,328	56,385	7,301
hsa-miR-562	46,539	6,239	44,731	8,009
hsa-miR-515-3p	49,225	5,210	47,314	6,545
hsa-miR-223-5p	55,492	6,415	53,342	5,205
hsa-miR-637	49,159	4,671	47,256	3,382
hcmv-miR-US33-5p	53,792	3,748	51,712	5,756
hsa-miR-3152-3p	45,445	5,556	43,689	5,303
hsa-miR-3670	48,095	7,083	46,237	5,046
hsa-miR-149-3p	42,260	4,237	40,628	4,865
hsa-miR-4278	46,725	6,402	44,922	4,845
hsa-miR-941	37,599	5,228	36,153	3,849
hsa-miR-644a	43,303	5,606	41,638	5,910
hsa-miR-3911	74,064	9,265	71,222	8,073
hsa-miR-548h-5p	42,916	4,996	41,270	3,258
hsv1-miR-H14-5p	44,437	5,402	42,734	4,615
bkv-miR-B1-3p	51,212	7,710	49,251	6,925
hsa-miR-1228-5p	115,504	9,152	111,085	11,880
hsa-miR-875-5p	49,511	5,232	47,619	5,602
hsa-miR-1914-5p	49,419	5,640	47,540	4,106
hsa-miR-331-5p	42,763	4,872	41,140	7,064
hsa-miR-2113	56,015	6,466	53,896	6,927
kshv-miR-K12-6-3p	41,534	5,891	39,964	2,537
hsa-miR-623	59,141	6,143	56,910	5,475
hsa-miR-593-3p	56,451	4,210	54,325	6,104
ebv-miR-BART1-3p	48,528	5,239	46,704	5,043
hsa-miR-302a-5p	46,098	4,180	44,368	7,012
hsa-miR-218-2-3p	40,336	4,860	38,830	4,036
hsa-miR-889	43,145	5,244	41,538	2,935
hsa-miR-374b-3p	51,720	5,898	49,797	4,041
hsa-miR-18a-3p	48,022	5,103	46,242	5,934
hsa-miR-1911-5p	46,782	3,863	45,049	4,454
hsa-miR-518f-5p	47,187	4,673	45,441	5,096
hsa-miR-3167	44,918	4,849	43,260	5,599
hsa-miR-2117	62,927	6,180	60,606	8,688
ebv-miR-BART3	42,742	4,798	41,167	6,302
hsa-miR-185-3p	48,377	5,978	46,598	7,001
hsa-miR-195-5p	52,933	7,017	50,996	5,145
hsa-miR-609	107,536	10,320	103,606	14,419
hsa-miR-192-3p	48,516	6,202	46,744	5,055
hsa-miR-1233	47,747	7,789	46,008	2,885
hsa-miR-2117	61,857	7,119	59,616	6,430
hsa-miR-499a-3p	43,854	6,003	42,267	5,260
hsa-miR-644a	44,108	6,865	42,517	3,640
hsa-miR-4252	106,000	7,937	102,177	16,912
hsa-miR-548h-3p	45,633	6,491	43,992	5,335
hsa-miR-337-5p	44,012	3,965	42,433	5,075
hsa-miR-325	48,370	5,275	46,638	7,562

hsa-miR-2116-3p	71,191	5,222	68,644	6,634
hsa-miR-455-5p	45,773	5,363	44,144	4,961
hsa-miR-200b-5p	45,541	3,574	43,922	6,014
hsa-miR-4252	117,100	9,587	112,941	19,067
hsa-miR-656	48,592	5,624	46,869	4,953
hsa-miR-589-3p	41,858	4,055	40,378	4,998
hsa-miR-1227	58,385	7,283	56,321	7,498
hsa-miR-555	43,514	3,792	41,979	2,963
hsa-miR-1297	43,641	5,008	42,103	3,287
hsa-miR-3185	42,571	6,094	41,071	3,843
hsa-miR-553	46,944	4,463	45,291	5,345
hsa-miR-671-3p	57,813	6,635	55,778	5,640
hsa-miR-924	50,166	3,944	48,400	4,512
hcmv-miR-UL22A*	42,400	5,755	40,908	5,105
hsa-miR-101-5p	55,896	6,920	53,931	7,103
hsa-miR-491-5p	46,283	3,842	44,665	3,131
hsa-miR-3147	60,300	8,207	58,193	7,265
hsa-miR-544a	49,058	6,075	47,345	5,121
hsa-miR-3161	55,214	5,710	53,285	6,542
hsa-miR-567	47,849	6,715	46,183	4,326
hsa-miR-92a-2-5p	43,990	5,904	42,460	5,586
hsa-miR-2116-3p	72,442	6,568	69,926	5,467
hsa-miR-338-3p	1372,577	768,776	1325,042	240,244
ebv-miR-BART8*	40,263	5,075	38,870	4,159
hsa-miR-569	43,967	3,968	42,450	3,793
hsa-miR-3147	54,443	6,563	52,570	6,845
hsa-miR-3692-3p	41,261	6,058	39,843	3,825
hsa-miR-3612	49,243	8,222	47,551	6,363
hsa-miR-2052	43,422	5,040	41,931	5,675
kshv-miR-K12-1	41,742	4,329	40,310	6,557
hsa-miR-3186-5p	49,151	5,569	47,471	5,057
hsa-miR-875-5p	47,711	6,562	46,084	5,344
hsa-miR-3675-5p	44,170	6,341	42,665	5,435
hsa-miR-657	46,714	5,846	45,125	6,508
hsa-miR-4295	44,942	5,595	43,419	5,015
hsa-miR-3924	50,517	8,277	48,806	4,834
hsa-miR-802	44,775	5,490	43,259	6,457
hsa-miR-4282	47,992	4,445	46,368	6,382
hsa-miR-1260b	1127,738	128,570	1089,586	351,549
hsa-miR-541-5p	63,096	4,996	60,964	16,967
hsa-miR-636	55,491	5,425	53,615	6,152
hsa-miR-602	69,751	7,176	67,400	5,574
hsv1-miR-H2*	47,973	6,971	46,359	5,027
hsa-miR-93-3p	52,868	7,514	51,094	8,007
hsa-miR-3683	44,179	5,005	42,698	3,632
ebv-miR-BART5	48,658	4,646	47,033	4,206
hsa-miR-187-3p	42,589	4,676	41,170	3,686

hsa-miR-596	41,890	3,111	40,497	5,049
kshv-miR-K12-9*	56,711	6,376	54,827	4,908
hsa-miR-616-3p	39,078	5,110	37,782	4,913
hsa-miR-486-3p	44,176	5,313	42,717	3,978
hsa-miR-3617	43,890	4,766	42,444	4,738
kshv-miR-K12-5	43,200	4,436	41,779	5,165
hsa-miR-625-5p	60,808	9,014	58,809	7,228
hsa-miR-363-5p	41,606	3,215	40,240	5,582
hsa-miR-552	44,388	4,937	42,936	5,369
ebv-miR-BART1-5p	52,610	4,414	50,889	7,734
ebv-miR-BART16	171,676	22,894	166,085	11,206
hsa-miR-3151	46,903	5,109	45,376	5,022
hsa-miR-127-5p	44,567	4,218	43,121	6,055
hsa-miR-103b	50,335	4,765	48,705	7,036
hsa-miR-1298	44,124	5,713	42,704	5,770
hsa-miR-26b-5p	9883,795	578,626	9566,234	813,888
hsa-miR-4265	45,675	6,311	44,213	5,410
hsa-miR-3913-5p	45,576	5,997	44,120	4,458
hsa-miR-4295	45,648	6,258	44,191	5,843
hsa-miR-211-5p	55,652	5,564	53,881	7,826
hsa-miR-579	46,231	5,987	44,762	5,852
hsa-miR-454-5p	73,125	13,477	70,812	15,521
hsa-miR-558	44,347	4,476	42,948	5,736
hsa-miR-665	52,816	6,295	51,153	7,125
hsa-miR-20b-3p	45,877	5,094	44,438	2,531
hsa-miR-625-3p	59,986	6,305	58,106	9,385
hsa-miR-1178	47,577	5,839	46,089	7,684
hsv1-miR-H7*	69,205	7,655	67,042	8,713
hsa-miR-3678-5p	46,336	4,458	44,889	5,955
ebv-miR-BART7*	44,421	5,608	43,039	5,297
hsa-miR-3673	42,266	5,125	40,951	5,322
hsa-miR-3939	42,355	5,768	41,038	4,927
hsa-miR-548a-5p	50,233	3,775	48,672	4,641
hsa-miR-450b-3p	51,895	5,409	50,284	7,157
hsa-miR-204-5p	56,561	6,031	54,807	10,041
hcmv-miR-US33-3p	78,973	7,046	76,527	21,166
hsa-miR-497-3p	42,496	7,396	41,182	6,075
hsa-miR-3194-5p	79,321	12,968	76,873	6,618
hsa-miR-410	44,072	5,764	42,711	5,555
hsa-miR-27b-5p	50,583	6,505	49,026	2,955
hsa-miR-578	43,939	3,063	42,592	5,530
hsa-miR-1283	41,151	5,416	39,891	3,942
hsa-miR-3614-5p	64,554	5,271	62,580	6,789
hsa-let-7f-2-3p	55,453	5,114	53,766	8,065
hsa-miR-4315	45,669	5,969	44,286	5,275
hsa-miR-3180	40,868	5,780	39,631	4,716
hsa-miR-641	52,999	6,935	51,397	8,203

hsa-miR-548a-3p	46,302	5,507	44,904	5,238
hsa-miR-3944-3p	43,590	6,774	42,275	4,385
ebv-miR-BART13*	43,072	3,602	41,776	6,071
hsa-miR-146b-3p	43,344	5,385	42,042	5,929
hsa-miR-379-3p	44,917	6,073	43,568	5,598
hsa-miR-490-3p	44,424	5,153	43,091	6,413
hsa-miR-125b-1-3p	41,122	3,739	39,892	4,862
kshv-miR-K12-11	43,641	5,661	42,340	5,016
hsa-miR-551a	47,336	6,004	45,926	3,596
hsa-miR-21-5p	117551,240	14940,736	114066,830	6499,055
hsa-miR-3180	40,289	4,926	39,101	5,207
hsa-miR-196a-3p	43,258	6,583	41,984	6,974
hsa-miR-3190-3p	64,479	7,022	62,582	8,076
hsa-miR-614	45,921	3,778	44,575	5,982
hsa-miR-1322	50,173	5,962	48,703	6,155
kshv-miR-K12-11	44,653	4,403	43,348	5,914
ebv-miR-BART16	110,350	11,349	107,129	14,319
hsa-miR-218-1-3p	40,717	5,370	39,533	5,409
hsa-miR-1231	38,647	6,293	37,525	4,967
hsa-miR-1914-5p	47,768	5,950	46,383	6,706
hsa-miR-510	43,748	6,558	42,480	5,340
hsa-miR-302e	49,913	5,945	48,468	6,809
hsa-miR-374a-3p	41,475	3,472	40,276	5,752
hsv1-miR-H7	41,157	4,648	39,974	4,255
kshv-miR-K12-9	41,569	5,463	40,375	5,592
hsa-let-7c*_v16.0	46,102	3,579	44,778	5,778
hsa-miR-4302	42,536	4,840	41,315	5,471
hsa-miR-625-3p	64,260	4,981	62,416	8,143
hsa-miR-521	49,710	4,463	48,284	7,999
hsa-miR-519d	54,420	6,140	52,862	7,031
hsa-miR-302a-3p	50,439	4,801	48,996	6,414
hsa-miR-373-5p	43,733	5,514	42,484	4,127
hsa-miR-4255	44,121	4,171	42,861	5,061
hsa-miR-371a-3p	48,343	6,413	46,964	6,736
hsa-miR-1286	46,925	5,553	45,589	4,603
hsa-miR-3144-5p	40,676	6,405	39,529	5,904
hsa-miR-618	46,062	6,219	44,763	8,000
hsa-miR-3675-5p	44,381	4,253	43,131	4,878
hsv1-miR-H2	42,217	4,410	41,035	5,092
hsa-miR-3186-3p	45,809	6,224	44,528	5,490
ebv-miR-BART5*	50,209	6,045	48,818	5,055
hsa-miR-525-3p	46,561	4,142	45,271	4,772
hsa-miR-1254	47,349	5,377	46,038	7,792
hsa-miR-1254	43,874	5,151	42,661	5,131
hsa-miR-4318	51,586	6,181	50,161	4,497
hsa-miR-3124-5p	43,709	5,117	42,509	5,565
hsa-miR-493-3p	46,717	5,878	45,437	6,331

hsa-miR-654-5p	47,102	6,399	45,813	6,295
hsa-miR-199a-5p	49,051	4,672	47,710	6,331
hsa-miR-512-3p	52,932	5,495	51,492	6,161
hsa-miR-608	44,643	4,346	43,431	5,184
hsa-miR-1248	40,143	4,619	39,056	5,350
hsa-miR-624-3p	50,669	7,950	49,298	6,355
hsa-miR-891b	47,126	4,649	45,857	6,325
hsa-miR-496	43,767	3,611	42,596	4,555
hsa-miR-219-2-3p	45,200	3,670	43,991	6,060
hsa-miR-329	49,407	4,664	48,087	6,532
hsv1-miR-H3*	49,051	4,184	47,741	3,502
hsa-miR-182-5p	65,929	6,317	64,170	17,702
hsa-miR-2113	63,530	6,467	61,838	8,084
hsa-miR-148b-5p	47,620	3,886	46,355	3,961
hsa-miR-99a-3p	45,980	5,297	44,759	5,252
kshv-miR-K12-10b	40,730	6,230	39,659	5,404
hcmv-miR-US5-2	40,458	4,588	39,396	3,845
kshv-miR-K12-6-3p	44,181	4,268	43,028	4,409
hsa-miR-412	44,070	6,104	42,921	4,544
hsa-miR-20b-3p	43,569	4,835	42,439	3,670
hsa-miR-187-5p	48,433	4,856	47,188	4,937
hsa-miR-888-5p	46,462	5,476	45,273	5,584
hsa-miR-548d-3p	48,700	4,911	47,456	6,184
hsa-miR-154-3p	45,872	4,877	44,702	5,289
hsa-miR-186-3p	46,334	4,155	45,155	6,340
hsa-miR-302d-5p	40,110	5,638	39,090	3,747
hsa-miR-3614-5p	64,570	4,745	62,930	8,102
kshv-miR-K12-11	47,368	5,657	46,167	4,383
hsa-miR-337-3p	57,314	5,710	55,868	6,489
hsa-miR-574-5p	485,774	100,550	473,528	42,499
hsa-miR-643	45,056	5,405	43,920	5,592
hsa-miR-488-5p	45,156	5,721	44,020	6,008
hsa-miR-658	46,695	4,277	45,521	8,686
hsa-miR-452-3p	39,750	5,169	38,756	4,109
hsa-miR-3150b-3p	45,527	4,721	44,389	5,805
hsa-miR-1236	53,680	3,951	52,338	9,064
hsa-miR-556-3p	47,586	5,017	46,400	5,609
hsv1-miR-H3	47,770	5,535	46,588	4,831
hsa-miR-187-5p	47,507	6,857	46,334	5,495
hsa-miR-4330	47,893	7,890	46,711	5,545
hsa-miR-548m	49,917	5,538	48,686	5,099
ebv-miR-BART6-5p	43,693	6,090	42,615	7,087
hsa-miR-3115	46,820	4,506	45,665	6,421
hsa-miR-27b-5p	49,983	6,721	48,755	5,361
hsa-miR-302b-5p	46,054	4,201	44,928	5,716
hsa-miR-548c-3p	47,200	5,422	46,047	6,185
ebv-miR-BART1-3p	46,973	5,850	45,844	5,214

hsa-miR-18b-3p	60,007	3,781	58,567	3,792
hsa-miR-302c-3p	46,388	4,793	45,276	5,191
hsa-miR-106a-3p	42,312	4,989	41,299	3,646
hsa-miR-1245a	45,390	6,330	44,310	6,065
hsa-miR-522-3p	47,731	5,526	46,605	6,324
hsa-miR-23c	65,172	4,535	63,638	7,062
hsa-miR-659-3p	49,345	3,681	48,184	5,691
hsa-miR-105-5p	48,953	5,495	47,802	4,675
ebv-miR-BART8	45,445	3,300	44,377	5,345
hsa-miR-665	52,101	3,243	50,878	6,305
hsv2-miR-H19	40,722	5,595	39,767	5,334
hsa-miR-19b-2-5p	46,914	4,143	45,814	6,132
hsa-miR-569	41,941	5,385	40,961	5,543
hsa-miR-609	97,382	6,904	95,109	17,595
hsa-miR-614	44,370	5,380	43,335	5,388
ebv-miR-BART1-5p	50,276	4,595	49,114	3,697
hsa-miR-559	44,479	4,616	43,451	3,380
hsa-miR-802	43,396	3,939	42,397	6,637
hsa-miR-19a-5p	44,966	3,096	43,933	5,832
hsa-miR-29b-2-5p	51,210	5,163	50,036	4,352
hsa-miR-18b-5p	50,077	6,884	48,930	6,372
hsa-miR-3920	48,128	6,389	47,025	6,191
hsa-miR-523-3p	43,587	5,701	42,591	6,367
hsa-miR-514a-3p	44,679	5,699	43,659	4,579
hcmv-miR-UL112	41,616	6,489	40,668	4,329
hsa-miR-1247-5p	46,671	4,532	45,611	4,797
hsa-miR-377-5p	46,270	6,160	45,220	5,395
hsa-miR-4315	45,500	5,797	44,475	5,687
hsa-miR-3662	51,213	5,430	50,062	5,623
hsa-miR-520b	52,709	8,305	51,527	6,204
hsa-miR-3131	44,858	5,517	43,856	6,683
hsa-miR-517c-3p	46,292	4,730	45,260	5,268
ebv-miR-BART17-3p	51,714	6,052	50,565	6,868
hsa-miR-574-5p	433,427	70,400	423,835	34,542
hsa-miR-296-3p	44,519	5,584	43,534	5,434
hsa-miR-671-3p	54,273	7,546	53,075	4,185
hsa-miR-133b	52,206	7,255	51,056	5,196
hsa-miR-938	43,125	5,544	42,183	4,451
hsa-miR-1263	46,344	5,512	45,335	5,941
hsa-miR-429	50,110	5,442	49,021	6,596
hsa-miR-181a-2-3p	46,414	2,501	45,407	4,850
hsa-miR-34c-5p	45,294	4,538	44,312	5,950
hsa-miR-544a	49,898	4,963	48,818	7,596
hsa-miR-662	57,833	3,696	56,581	5,484
hsa-miR-323b-5p	46,293	4,306	45,292	7,512
hsa-miR-196a-3p	42,943	5,042	42,015	5,328
hsa-miR-3910	44,184	3,728	43,230	5,719

hsa-miR-1263	44,839	6,076	43,872	5,843
hsa-miR-875-3p	44,650	5,763	43,691	5,688
hsa-miR-892a	50,839	7,213	49,749	6,115
hsa-miR-615-5p	42,171	6,037	41,267	5,452
hsa-miR-146a-3p	47,687	6,020	46,670	5,531
hsa-miR-218-2-3p	45,327	3,155	44,369	4,760
ebv-miR-BHRF1-3	40,979	4,392	40,115	4,248
hsa-miR-3674	45,394	5,193	44,438	5,038
kshv-miR-K12-7	44,060	4,259	43,135	5,634
hsa-miR-4263	46,505	4,031	45,533	3,934
hsa-miR-345-5p	59,977	5,636	58,728	5,513
hsa-miR-4285	39,331	5,633	38,515	4,828
hsa-miR-3150a-3p	42,353	6,098	41,477	5,346
hsa-miR-3122	44,812	5,987	43,890	7,013
ebv-miR-BART16	175,476	25,349	171,877	22,912
hsa-miR-514b-5p	62,995	7,993	61,703	8,355
hsa-miR-34c-3p	47,044	7,601	46,080	6,806
hsa-miR-588	48,395	6,479	47,403	5,093
hsa-miR-548a-3p	44,408	5,173	43,502	5,070
hsa-miR-548n	46,120	5,226	45,179	5,772
hsa-miR-1286	46,106	6,328	45,169	5,718
hsa-miR-488-3p	44,948	4,436	44,040	6,012
hsa-miR-708-5p	46,734	5,517	45,794	7,219
hsa-miR-143-5p	46,968	5,265	46,024	5,423
hsa-miR-569	40,322	6,448	39,516	4,628
hsa-miR-3928	44,133	4,172	43,255	5,886
hsa-miR-610	62,147	6,435	60,911	5,138
hsa-let-7a-3p	49,384	6,520	48,402	5,225
hsa-miR-3121-3p	46,353	5,304	45,432	5,213
hsa-miR-3605-3p	52,787	5,882	51,741	7,111
hsa-miR-599	43,564	4,355	42,702	3,259
hsa-miR-363-5p	43,205	5,426	42,354	3,688
hsa-miR-218-5p	48,666	5,890	47,708	6,839
kshv-miR-K12-6-3p	39,306	6,417	38,536	6,148
hsa-miR-1273e	55,010	7,135	53,937	4,837
hsa-miR-432-3p	55,033	7,464	53,960	6,739
hsa-miR-192-3p	47,844	6,363	46,911	5,600
hsa-miR-616-5p	47,428	6,686	46,506	6,604
hsa-miR-429	49,819	4,592	48,854	5,454
hsa-miR-551a	51,996	6,737	50,989	5,313
kshv-miR-K12-9*	52,546	4,305	51,534	6,163
hsa-miR-875-5p	46,383	4,918	45,490	7,334
hsa-miR-556-3p	50,512	5,836	49,541	6,160
hsa-miR-607	48,270	6,636	47,341	6,476
hsa-miR-548g-3p	46,922	5,196	46,021	5,058
hsa-miR-296-3p	42,547	4,910	41,732	4,739
hiv1-miR-N367	53,610	5,181	52,590	5,984

hsa-miR-155-3p	48,580	6,925	47,659	7,589
hsa-miR-509-3p	45,425	7,250	44,568	5,703
ebv-miR-BHRF1-2	41,101	2,894	40,339	5,346
hsa-miR-3613-5p	49,824	4,895	48,905	5,969
hsa-miR-3942-5p	47,783	3,481	46,909	6,070
hsa-miR-548x_v16.0	47,284	4,287	46,419	3,991
hsa-miR-921	48,882	3,885	47,988	6,450
hsa-miR-124-5p	43,533	6,023	42,738	5,960
hsa-miR-486-3p	44,479	6,536	43,667	6,191
hsa-miR-490-5p	51,094	5,361	50,167	6,003
hsa-miR-4251	42,565	5,364	41,794	5,006
ebv-miR-BART20-3p	45,343	4,737	44,527	4,660
hcmv-miR-UL22A	48,228	5,311	47,363	5,197
ebv-miR-BHRF1-2	41,357	4,890	40,616	7,275
hsa-miR-3649	43,060	4,980	42,289	5,345
hsa-miR-183-5p	46,593	4,193	45,763	3,494
hsa-miR-490-5p	47,972	6,895	47,120	4,084
hsa-miR-4311	58,029	6,155	57,001	4,700
ebv-miR-BART6-3p	39,720	4,445	39,016	4,008
hsa-miR-548h-5p	45,326	4,513	44,526	6,054
hsa-miR-634	52,369	4,786	51,445	7,406
hsa-miR-26b-5p	5618,276	169,315	5519,234	406,859
hsa-miR-3144-3p	47,690	5,466	46,850	8,056
hsa-miR-1224-3p	65,948	5,676	64,788	9,203
hsa-miR-596	44,603	4,213	43,820	5,459
hsa-miR-938	40,790	5,057	40,075	4,172
hsa-miR-153	42,872	4,677	42,124	5,551
ebv-miR-BART11-5p	45,538	7,537	44,745	6,606
hsa-miR-624-3p	52,375	6,528	51,464	8,370
hsa-miR-551b-5p	40,740	7,625	40,032	3,787
hsv2-miR-H11*	45,912	5,242	45,120	5,936
hsa-miR-488-5p	46,245	5,563	45,449	5,456
hsa-miR-562	47,002	4,564	46,194	5,306
hsa-miR-648	49,892	4,967	49,036	7,304
hsv2-miR-H5	66,454	4,729	65,315	8,415
hsa-miR-3161	48,288	4,951	47,462	5,418
hsa-miR-4288	47,840	4,762	47,022	4,749
hsa-miR-620	42,116	5,102	41,396	5,794
hsa-miR-4265	44,296	4,444	43,544	5,098
hsa-miR-518c-5p	46,665	4,873	45,877	5,795
hsa-miR-545-5p	43,117	4,368	42,393	4,619
hsv1-miR-H11	41,866	5,189	41,163	5,661
hsa-miR-938	40,305	6,989	39,635	5,376
hsa-miR-129-5p	50,975	4,714	50,129	5,997
hsa-miR-184	48,416	5,673	47,616	6,132
hsa-miR-3679-3p	53,261	5,698	52,382	7,103
hsa-miR-3158-3p	47,124	5,388	46,353	5,798

hsa-miR-330-5p	44,412	6,120	43,688	6,840
hsa-miR-3936	42,862	5,579	42,164	4,702
hsa-miR-1184	45,409	4,585	44,675	6,983
hsa-miR-1224-3p	64,522	6,137	63,482	12,463
hsa-miR-216b	39,655	3,493	39,020	4,591
hsa-miR-609	91,232	7,362	89,771	12,589
hsa-miR-325	44,680	5,535	43,965	6,308
hcmv-miR-US25-1	46,614	5,100	45,873	7,572
hsa-miR-2277-3p	89,034	11,352	87,629	8,885
hsa-miR-562	48,315	5,153	47,554	6,454
hsa-miR-579	53,333	7,507	52,494	5,777
hsa-miR-761	48,656	4,884	47,893	7,784
hsa-miR-513a-3p	49,605	7,629	48,827	6,121
hsa-miR-573	44,123	4,756	43,432	5,793
hsa-miR-759	44,410	5,306	43,720	5,440
ebv-miR-BART9	46,948	4,758	46,225	5,973
hsa-miR-610	57,523	6,247	56,640	6,320
hsa-miR-3669	44,959	4,820	44,274	6,291
ebv-miR-BART10*	51,923	4,666	51,134	5,236
hsa-miR-135a-3p	114,570	24,859	112,831	28,590
hsa-miR-4275	48,465	5,893	47,734	5,925
hsa-miR-629-5p	59,917	8,721	59,014	7,447
hsa-miR-652-3p	107,504	17,328	105,886	9,628
ebv-miR-BART13*	44,069	5,540	43,408	3,932
hsa-miR-223-5p	123,504	12,540	121,664	10,910
hsa-miR-450b-3p	56,316	6,263	55,479	5,712
hsa-miR-3613-3p	91,974	8,549	90,614	22,782
hsa-miR-889	45,389	4,830	44,719	5,998
hsa-miR-375	40,724	5,649	40,130	5,497
hsv1-miR-H12	40,889	4,491	40,296	5,818
ebv-miR-BHRF1-3	42,171	6,009	41,560	5,884
hsa-miR-548l	47,319	5,582	46,634	6,253
hsa-miR-4308	48,960	5,277	48,256	5,776
hcmv-miR-UL112	43,186	4,589	42,566	4,160
hsa-miR-548a-5p	53,762	7,872	52,992	9,087
hsv1-miR-H16	77,206	7,825	76,106	5,159
hsa-miR-325	44,034	6,457	43,407	3,309
hsa-miR-526b-5p	45,862	5,809	45,211	6,527
hsa-miR-105-5p	46,451	6,441	45,801	8,433
ebv-miR-BART21-3p	47,580	4,734	46,916	5,212
hsa-miR-455-3p	45,369	4,172	44,738	5,025
hsa-miR-922	41,607	6,007	41,030	6,855
hsa-miR-4316	47,918	5,276	47,256	5,875
hsa-miR-136-3p	44,901	6,864	44,288	5,060
hsa-miR-548b-3p	49,638	6,221	48,961	6,843
hsa-miR-3115	47,188	7,639	46,544	4,957
hsa-miR-3119	45,901	5,250	45,275	5,372

hsa-miR-1204	47,508	5,990	46,870	6,091
hsa-miR-3929	47,873	5,948	47,232	8,298
hsa-miR-2114-5p	43,880	5,392	43,294	6,102
hsa-let-7f-2-3p	52,802	5,899	52,103	8,194
hsa-miR-942	44,481	3,442	43,898	5,594
hsa-miR-3173-3p	50,617	4,488	49,959	5,803
hsa-miR-105-3p	44,283	5,418	43,713	6,056
hsa-miR-302f	48,707	5,380	48,082	5,896
hsa-miR-548d-3p	48,923	5,959	48,297	6,238
hsa-miR-129-2-3p	53,504	5,905	52,822	7,899
hsa-miR-654-5p	60,074	6,496	59,312	8,073
hsa-miR-3143	41,278	4,654	40,758	5,060
kshv-miR-K12-4-5p	45,837	3,450	45,260	5,760
hsa-miR-19b-2-5p	45,097	4,268	44,530	4,970
ebv-miR-BART1-5p	47,178	7,182	46,590	5,706
hsa-miR-3201	40,497	4,864	39,996	5,362
hsa-miR-297	40,842	4,789	40,338	4,537
hsa-miR-580	41,252	4,012	40,744	4,983
hsa-miR-555	46,783	4,775	46,213	5,525
hsa-miR-26a-2-3p	48,166	4,140	47,579	4,971
hsv1-miR-H5-5p	46,079	5,465	45,518	3,621
hsa-miR-590-3p	45,794	4,335	45,240	4,876
hsa-miR-3924	48,815	5,553	48,227	4,989
hsa-miR-92a-2-5p	45,353	5,657	44,807	6,082
hsa-miR-933	55,565	3,874	54,897	6,158
hsa-miR-488-3p	46,611	3,754	46,056	5,142
hsa-miR-632	48,619	6,647	48,042	5,207
hsa-miR-449c-5p	54,921	6,663	54,272	4,569
hsa-miR-606	44,319	5,963	43,799	5,254
hsa-miR-708-3p	43,490	6,440	42,981	4,076
kshv-miR-K12-6-5p	42,607	5,455	42,110	5,528
hsa-miR-96-3p	46,160	5,944	45,623	5,278
hsa-miR-1267	59,763	4,191	59,069	7,061
hsa-miR-541-5p	58,728	5,987	58,047	11,866
hsa-miR-216a	44,078	5,719	43,569	4,182
ebv-miR-BART8*	41,214	5,321	40,738	4,993
hsv1-miR-H14-3p	43,173	5,707	42,676	4,773
hsa-miR-183-5p	44,664	6,194	44,151	4,310
hsa-miR-653	45,246	5,869	44,727	4,501
hsa-miR-4294	53,881	5,245	53,267	5,158
hsv1-miR-H7	40,909	4,453	40,444	4,461
hsa-miR-27b-5p	46,922	4,116	46,390	4,196
hsa-miR-205-5p	46,034	4,654	45,515	5,674
hsa-miR-657	51,824	6,238	51,245	4,786
hsa-miR-3168	44,498	5,299	44,003	5,819
hsa-miR-759	43,078	4,830	42,600	5,779
hsa-miR-1226-3p	43,684	5,975	43,199	7,374

hsa-miR-493-3p	45,637	5,649	45,131	4,713
hsa-miR-3169	40,729	4,144	40,279	5,736
hsa-miR-4305	55,569	5,244	54,958	9,747
hsa-miR-452-3p	40,142	5,009	39,702	6,150
hsa-miR-485-5p	42,127	5,214	41,668	3,714
kshv-miR-K12-4-5p	43,692	4,337	43,220	5,430
hsa-miR-516a-3p	47,124	5,480	46,616	4,273
hsa-miR-1273a	43,988	5,056	43,516	5,498
hsa-miR-636	57,206	6,944	56,595	5,755
hsa-miR-454-5p	89,510	12,507	88,560	14,048
hsa-miR-3200-5p	43,987	5,484	43,525	7,236
hsa-miR-3674	43,322	6,089	42,867	3,257
hsa-miR-4326	59,251	7,129	58,630	7,906
hsa-miR-568	65,496	8,201	64,814	16,280
hsa-miR-141-5p	44,737	6,331	44,273	5,466
hsa-miR-4268	44,537	3,342	44,077	4,921
hsa-miR-3190-3p	59,266	5,699	58,663	8,050
hsa-miR-365b-5p	44,485	4,968	44,035	7,549
hsa-miR-526b-3p	49,083	6,320	48,592	6,993
hsa-miR-612	46,130	5,069	45,668	3,669
hsa-miR-944	44,956	4,763	44,507	3,769
kshv-miR-K12-2*	43,794	6,010	43,358	4,919
hcmv-miR-US25-2-5p	51,415	7,587	50,904	2,942
hsa-miR-585	43,904	4,519	43,467	5,973
ebv-miR-BART6-3p	40,316	5,416	39,920	5,102
ebv-miR-BART10	85,274	9,110	84,444	5,366
hsa-miR-373-3p	55,086	5,023	54,555	5,890
hsa-miR-1323	51,242	3,956	50,748	4,598
hsa-miR-3662	47,644	7,289	47,186	6,888
hsa-miR-520e	50,063	5,577	49,582	4,140
hsa-miR-591	43,233	5,233	42,820	3,641
hsa-miR-3650	58,114	5,970	57,564	4,920
hsa-miR-25-5p	43,053	3,868	42,650	6,478
hsa-miR-499a-3p	44,160	3,570	43,748	4,669
hsa-miR-3199	43,861	5,495	43,454	4,465
hsa-miR-587	43,351	4,333	42,951	4,288
hsa-miR-3169	42,032	5,093	41,646	3,948
hsa-miR-767-3p	51,386	5,559	50,921	4,617
hsa-miR-2909	44,101	4,534	43,703	5,433
hsa-miR-103b	52,152	5,105	51,686	5,291
hsa-miR-371a-3p	47,230	6,372	46,808	4,867
hsa-miR-1908	44,093	4,914	43,703	4,796
hsa-miR-1204	45,679	5,555	45,277	5,990
hsa-miR-548av-3p	46,124	6,404	45,723	6,257
hsa-miR-1267	59,881	3,734	59,367	5,978
hsa-miR-593-5p	41,138	4,938	40,786	4,956
hsa-miR-597	46,328	5,640	45,934	6,158

hsa-miR-1289	44,098	4,135	43,723	4,538
hsa-miR-1185-5p	44,679	4,287	44,299	6,844
hsa-miR-432-5p	59,255	5,454	58,752	5,908
hsa-miR-633	47,838	6,111	47,435	5,424
hsa-miR-1251	42,788	5,460	42,428	5,382
kshv-miR-K12-5	42,576	4,491	42,219	5,715
hsa-miR-92a-2-5p	48,170	5,926	47,769	5,009
ebv-miR-BART15	46,594	5,394	46,212	6,239
hsa-miR-2116-3p	69,757	5,733	69,186	5,480
hsa-miR-512-3p	49,793	5,399	49,389	5,423
hsa-miR-1283	40,069	3,830	39,745	5,921
ebv-miR-BART11-5p	45,214	5,303	44,850	6,222
hsa-miR-518f-3p	45,499	6,052	45,137	4,764
hsa-miR-550b-3p	43,732	4,584	43,386	3,936
hsa-miR-544a	51,903	5,819	51,491	7,094
hsa-miR-28-3p	43,676	6,591	43,330	4,968
hsa-miR-1252	41,639	4,457	41,310	5,168
hsa-miR-3115	45,599	4,447	45,240	5,962
hsa-miR-626	45,765	7,693	45,406	6,858
hsa-miR-512-5p	46,552	4,774	46,188	5,442
hsa-miR-3173-3p	47,988	5,585	47,614	5,922
hsa-miR-888-3p	43,716	5,269	43,379	6,562
hsa-miR-624-5p	48,454	3,211	48,082	4,608
hsa-miR-526b-5p	50,673	5,919	50,286	5,488
hsa-miR-1193	41,056	6,067	40,746	7,290
hsa-miR-876-3p	42,417	4,283	42,097	5,342
hsa-miR-224-3p	45,262	5,458	44,922	5,872
hsa-miR-217	41,959	5,140	41,650	5,489
hsa-miR-3668	41,411	4,946	41,107	5,864
hsa-miR-135b-5p	51,071	5,243	50,701	5,846
hsa-miR-626	44,816	3,390	44,493	6,961
hsa-miR-3939	45,297	5,403	44,976	5,636
hsa-miR-3908	44,519	5,091	44,206	5,950
hsa-miR-3157-5p	44,896	5,299	44,584	5,836
hsa-miR-4309	46,997	5,081	46,674	6,622
hsa-miR-196a-5p	51,340	4,251	50,996	6,465
hsa-miR-3151	43,715	5,202	43,423	6,064
hsa-miR-1912	40,917	4,850	40,644	5,317
ebv-miR-BART18-3p	43,211	5,756	42,925	4,745
hsa-miR-376c	41,661	4,564	41,386	4,487
hsa-miR-522-3p	46,101	4,720	45,801	6,185
hsa-miR-1276	47,698	5,847	47,393	6,971
hsa-miR-323a-5p	42,577	3,437	42,314	4,152
hsa-miR-3605-3p	52,685	5,389	52,360	6,400
hsa-miR-520g	48,866	4,514	48,573	7,031
hsa-miR-518f-5p	45,582	4,473	45,311	5,888
hsa-miR-154-5p	52,431	5,193	52,120	6,086

hsa-miR-1321	44,869	5,670	44,604	4,065
hsa-miR-26b-3p	48,494	5,840	48,208	5,120
hsa-miR-662	58,315	6,711	57,972	7,550
hsa-miR-495	47,206	5,927	46,928	6,572
hsa-miR-550b-3p	42,507	4,897	42,258	5,431
ebv-miR-BART10	76,652	8,898	76,208	6,980
hsa-miR-3177-3p	47,536	6,217	47,263	5,768
hsa-miR-518e-5p	42,267	6,104	42,027	5,882
hsa-miR-647	120,078	8,988	119,397	37,355
hsa-miR-566	59,402	6,063	59,074	8,993
hsa-miR-944	48,495	7,053	48,227	6,686
hsa-let-7c*_v16.0	47,658	4,531	47,395	4,645
hsa-miR-515-5p	46,972	5,135	46,716	5,173
hsa-miR-612	44,590	5,141	44,348	6,537
hsa-miR-200a-5p	48,408	5,386	48,150	7,062
hsa-miR-376c	44,018	3,659	43,783	5,323
hsa-miR-214-5p	47,182	7,494	46,932	6,268
hsa-miR-3914	41,275	6,140	41,059	3,789
hsa-miR-29b-2-5p	49,275	5,702	49,020	6,022
hsa-miR-556-5p	43,549	6,693	43,324	5,241
hsa-miR-345-5p	67,081	18,575	66,738	16,531
hsa-miR-3175	42,270	6,378	42,057	5,212
hsv2-miR-H13	39,923	4,304	39,723	4,843
hsa-miR-518e-5p	43,849	5,218	43,631	7,597
hsa-miR-603	47,053	4,655	46,822	4,934
hcmv-miR-US25-1*	45,206	4,025	44,985	4,557
hsa-miR-15b-3p	47,877	6,055	47,644	6,152
ebv-miR-BART8*	38,756	3,350	38,568	3,709
hsa-miR-3605-5p	56,352	9,006	56,080	7,772
hsa-miR-4292	40,530	5,122	40,335	5,783
hsa-miR-558	43,032	5,760	42,826	7,602
ebv-miR-BART6-5p	40,645	5,527	40,452	3,972
hsa-miR-141-5p	40,313	3,350	40,121	5,073
hsa-miR-711	55,468	6,343	55,205	6,626
ebv-miR-BART19-5p	45,877	5,604	45,659	6,565
hsa-miR-191-3p	79,316	9,225	78,940	6,012
hsa-miR-219-1-3p	38,437	3,130	38,257	4,991
hsa-miR-338-3p	473,437	246,205	471,218	56,122
hsa-miR-1469	43,107	5,491	42,906	6,408
hsa-miR-15a-3p	50,495	4,921	50,261	6,865
ebv-miR-BART11-3p	47,504	5,581	47,289	5,483
hsa-miR-552	43,678	6,742	43,483	5,351
hsa-miR-641	57,555	6,705	57,299	7,659
kshv-miR-K12-1*	61,170	6,785	60,900	12,330
hsa-miR-548a-3p	45,404	3,868	45,207	6,014
hsa-miR-518b	45,869	5,033	45,670	4,609
hsa-miR-3160-3p	45,249	3,231	45,054	5,277

hsa-miR-524-3p	46,887	4,549	46,696	4,898
hsa-miR-454-5p	78,816	11,078	78,498	13,235
hsa-miR-3660	45,291	3,705	45,112	4,538
hsa-miR-647	86,869	9,153	86,526	20,799
hsa-miR-559	44,855	5,036	44,678	5,580
hsa-miR-379-3p	45,146	4,145	44,972	4,810
hsa-miR-3175	40,257	5,192	40,102	5,427
hsa-miR-517-5p	49,196	5,051	49,009	5,564
hsa-let-7g-3p	54,613	5,440	54,407	4,560
hsa-miR-633	45,350	5,254	45,179	2,662
hsa-miR-1193	42,568	7,485	42,409	4,865
hsa-miR-651	50,360	6,141	50,172	6,145
ebv-miR-BART10	80,497	6,488	80,200	7,379
hsv1-miR-H13	41,347	7,104	41,199	4,787
hsa-miR-524-3p	45,523	5,186	45,360	5,638
kshv-miR-K12-1	40,961	4,502	40,814	4,542
hsa-miR-520g	51,110	5,561	50,931	6,102
hsa-miR-188-3p	41,116	4,693	40,974	4,916
hsa-miR-124-5p	40,049	5,746	39,911	3,575
hsa-miR-31-3p	46,431	6,097	46,277	5,841
hsa-miR-193b-3p	616,810	104,633	614,814	91,683
hsa-miR-3928	42,931	5,157	42,793	6,041
hsa-miR-521	46,992	5,464	46,842	4,183
hsa-miR-211-5p	57,756	5,448	57,575	5,448
hsa-miR-4292	40,246	5,480	40,123	5,726
hsa-miR-100-3p	44,664	5,483	44,533	5,428
hsa-miR-675-3p	68,729	8,613	68,531	16,210
hsa-miR-520h	46,405	5,916	46,272	4,381
hsa-miR-191-3p	73,344	6,807	73,135	2,785
hsa-miR-3120-3p	48,504	6,798	48,367	5,656
hsa-miR-92a-1-5p	42,291	5,742	42,174	5,871
hsa-miR-29b-2-5p	51,622	5,550	51,479	5,498
hsa-miR-122-3p	48,836	5,368	48,702	5,833
hsa-miR-600	43,903	3,276	43,785	6,684
hsa-miR-372	45,130	6,055	45,011	3,911
hsa-miR-3619-5p	41,765	6,860	41,655	4,172
ebv-miR-BART1-3p	46,304	5,411	46,185	4,819
hsa-miR-1269a	42,447	5,798	42,339	5,799
hsa-miR-515-3p	47,980	5,824	47,860	6,016
hsa-miR-374a-3p	44,308	4,965	44,201	5,455
hsa-miR-373-3p	55,368	5,337	55,236	6,467
hsa-miR-4310	67,144	6,016	66,985	6,047
hsa-miR-3183	43,472	4,319	43,370	3,621
hsa-miR-320c	279,057	121,280	278,401	79,765
hsa-miR-224-5p	53,205	5,339	53,081	7,341
hsa-miR-1911-5p	44,335	5,169	44,233	5,794
hsa-miR-3166	43,932	4,945	43,832	5,839

kshv-miR-K12-12	44,152	4,299	44,056	6,891
hsa-miR-548c-3p	48,481	8,040	48,380	4,236
hsa-miR-505-5p	100,132	22,850	99,928	9,009
ebv-miR-BART6-3p	39,908	4,162	39,828	6,490
hsa-miR-518d-3p	47,193	5,302	47,101	6,427
hsa-miR-369-5p	48,480	4,744	48,387	4,846
hsa-miR-3909	40,717	5,859	40,638	6,111
hsa-miR-2355-5p	56,522	9,057	56,418	5,114
ebv-miR-BART18-5p	48,344	6,177	48,259	3,525
hcmv-miR-UL36	45,209	5,567	45,131	5,292
hsa-miR-606	44,519	5,984	44,451	5,691
hsa-miR-2277-5p	45,380	4,780	45,312	6,731
hsa-miR-561-3p	43,798	6,010	43,733	5,277
hsa-miR-147b	41,789	5,845	41,729	4,363
kshv-miR-K12-4-5p	42,928	4,997	42,868	6,776
hsa-miR-888-3p	42,483	7,174	42,425	5,421
hsa-miR-514a-3p	44,641	5,558	44,581	4,863
hsa-miR-671-3p	55,537	3,342	55,463	6,675
hsa-miR-3139	43,806	3,680	43,752	6,792
hsa-miR-3133	46,406	5,297	46,353	6,515
hsa-miR-551b-5p	43,461	5,050	43,412	5,673
hsa-miR-941	42,187	5,342	42,139	5,630
hsa-miR-1184	48,834	5,712	48,780	5,543
hsa-miR-3177-3p	48,688	4,937	48,635	6,789
ebv-miR-BART17-5p	39,094	5,058	39,053	6,399
hsa-miR-655	45,385	5,053	45,338	4,941
hcmv-miR-US25-2-5p	51,155	5,227	51,109	7,390
hsa-miR-3134	45,303	5,440	45,263	6,207
hsa-miR-485-5p	41,662	4,702	41,626	5,023
hsa-miR-181a-2-3p	47,841	3,587	47,800	4,812
hsa-miR-651	42,383	5,896	42,348	6,236
hsa-miR-4309	47,366	6,205	47,328	5,234
hsa-miR-346	57,003	6,284	56,969	7,393
hsa-miR-337-3p	57,120	5,540	57,088	5,672
ebv-miR-BART4	55,437	7,217	55,415	7,563
hsa-miR-339-3p	50,957	4,709	50,937	5,356
hsa-miR-566	62,558	4,659	62,534	11,915
hsa-miR-191-3p	74,889	7,732	74,864	7,912
hsa-miR-3199	41,847	4,866	41,836	4,674
hsa-miR-499a-3p	43,963	6,296	43,951	3,462
hsa-miR-3667-3p	47,178	5,383	47,168	5,815
hsa-miR-508-5p	42,574	5,594	42,566	4,534
hsa-miR-200b-5p	42,114	3,644	42,108	3,092
hsa-miR-1226-3p	41,119	4,555	41,115	4,849
hsa-miR-625-3p	62,569	4,980	62,564	11,875
hsa-miR-23c	67,656	5,397	67,653	5,957
hsa-miR-548a-3p	46,600	4,995	46,602	4,798

hsa-miR-323b-3p	38,970	4,874	38,973	5,211
hsa-miR-297	44,713	6,442	44,719	5,220
hsa-miR-934	39,367	3,073	39,373	4,660
hsa-miR-3927	43,544	4,534	43,551	3,969
hsa-miR-34c-5p	46,470	6,024	46,478	5,595
hsa-miR-934	44,157	6,937	44,168	4,930
hsa-miR-205-3p	136,240	16,830	136,276	37,155
hsa-miR-3128	42,790	6,147	42,802	5,562
hsa-miR-548x_v16.0	44,081	5,946	44,094	3,924
hsa-miR-369-5p	47,690	4,865	47,705	6,664
hsa-miR-4328	45,147	3,630	45,164	5,230
hsa-miR-3925-5p	56,930	6,897	56,953	10,895
hsa-miR-520a-5p	43,082	5,460	43,099	6,869
hsa-miR-302b-3p	47,895	7,760	47,921	3,348
ebv-miR-BART6-5p	42,637	4,432	42,660	6,680
hsa-miR-3661	41,753	5,142	41,778	4,900
ebv-miR-BART17-5p	41,252	3,513	41,277	3,607
hsa-miR-379-5p	42,859	5,946	42,888	3,996
hsa-miR-1324	45,489	5,058	45,520	3,356
hsa-miR-676-5p	43,777	5,473	43,808	5,721
hsa-miR-507	44,168	4,786	44,201	6,185
hsa-miR-591	46,423	4,005	46,460	6,971
hsa-miR-610	59,386	6,125	59,433	6,137
hsa-miR-618	44,424	5,545	44,460	4,481
hsa-miR-92a-1-5p	43,133	4,904	43,175	5,962
hsa-miR-590-3p	44,560	6,202	44,605	4,124
hsa-miR-3137	67,633	8,662	67,712	10,998
hsa-miR-4266	41,460	4,118	41,512	5,094
hsa-miR-3150b-3p	43,784	5,322	43,841	5,514
hsv2-miR-H4-3p	44,011	6,882	44,069	5,675
hsa-miR-124-5p	42,138	4,677	42,199	4,934
hsa-miR-921	47,778	3,712	47,848	6,422
hsa-miR-196a-5p	51,980	6,000	52,062	5,939
hsa-miR-875-3p	43,132	5,387	43,201	7,114
hsa-let-7c*_v16.0	46,321	4,333	46,397	4,950
hsa-miR-4255	43,692	4,340	43,764	4,839
hsa-miR-16-2-3p	138,380	25,947	138,611	6,649
hsa-miR-3915	43,858	5,794	43,931	4,609
hsa-miR-223-5p	148,417	12,297	148,680	13,806
hsa-miR-3672	44,544	4,995	44,625	4,460
hsa-miR-183-3p	38,808	3,686	38,877	5,080
hsa-miR-591	43,885	4,844	43,969	4,346
hsa-miR-3179	42,411	5,838	42,492	7,540
hsa-miR-3606	44,764	5,706	44,850	4,864
hsa-miR-888-5p	45,163	4,870	45,251	5,517
hsa-miR-3158-3p	48,231	6,512	48,326	5,433
hsa-miR-3920	48,367	4,667	48,462	4,276

kshv-miR-K12-2*	44,195	5,122	44,284	6,291
hsa-miR-302f	47,137	5,355	47,235	4,173
hsa-miR-452-3p	43,000	5,803	43,089	4,111
hsa-miR-626	43,547	5,697	43,642	5,982
hsa-miR-3913-5p	44,622	3,914	44,719	5,161
hsa-miR-3133	47,197	6,311	47,301	5,164
hsa-miR-224-5p	53,892	6,248	54,013	8,620
hsa-miR-187-5p	44,182	4,884	44,285	4,937
kshv-miR-K12-3*	41,587	4,886	41,687	4,388
hsa-miR-767-5p	45,459	3,160	45,568	4,905
hsa-miR-556-3p	49,770	5,044	49,892	4,863
hsa-miR-1270	46,480	5,516	46,597	4,794
hsa-miR-1272	43,304	4,992	43,413	5,401
hsa-miR-708-5p	47,378	4,950	47,498	3,919
hsa-miR-645	56,400	6,240	56,544	8,043
kshv-miR-K12-1	43,176	4,378	43,289	4,422
hsa-miR-1273e	62,493	7,162	62,666	9,751
hsa-miR-3681-3p	75,056	8,704	75,271	23,478
hsa-miR-127-3p	63,023	5,431	63,206	6,906
hsa-miR-4266	41,604	3,861	41,727	5,638
hsa-miR-588	47,385	4,094	47,526	6,833
hsa-miR-23b-5p	44,161	5,054	44,295	6,716
hsa-miR-504	45,841	3,507	45,984	7,075
hsa-miR-519e-3p	51,852	5,754	52,013	3,097
hsa-miR-302c-3p	51,213	7,035	51,376	6,947
hsa-miR-4273	45,361	5,322	45,512	5,026
hsa-miR-2053	48,726	4,247	48,894	5,731
hsa-miR-611	43,392	6,747	43,542	4,080
hsa-miR-635	46,068	4,063	46,229	4,373
hsa-miR-100-3p	44,245	5,903	44,402	4,713
hsa-miR-548d-3p	45,661	3,388	45,827	5,309
hsa-miR-563	63,427	6,255	63,661	5,600
hsa-miR-487a	44,540	5,502	44,705	4,382
hsa-miR-610	54,330	6,536	54,531	7,605
hsa-miR-19a-5p	49,319	5,238	49,506	8,572
kshv-miR-K12-11	45,919	5,865	46,095	4,038
hsa-miR-3171	47,941	4,479	48,130	5,770
hsa-miR-127-5p	41,026	4,872	41,188	5,212
hsa-miR-3126-5p	44,098	5,176	44,274	4,953
hsa-miR-331-5p	41,263	3,831	41,429	5,813
hsa-miR-155-3p	45,304	5,849	45,486	3,933
hsv1-miR-H5-3p	50,685	4,714	50,892	8,894
hsa-miR-663b	41,376	5,148	41,554	5,799
ebv-miR-BART3	38,550	2,671	38,727	5,064
ebv-miR-BART2-3p	40,904	5,160	41,091	4,912
hsa-miR-3131	47,701	5,291	47,922	5,864
hsa-miR-548t-5p	50,187	5,738	50,420	5,708

hsa-miR-92b-5p	40,974	5,061	41,165	5,398
hsa-miR-4282	46,828	4,548	47,049	4,747
hsa-miR-1243	48,147	5,300	48,375	4,347
hsa-miR-517-5p	48,767	6,913	48,998	7,581
hsa-miR-1257	45,629	5,579	45,848	5,017
hsa-miR-19a-5p	46,083	5,056	46,310	6,699
hiv1-miR-N367	48,549	5,579	48,789	4,456
hcmv-miR-UL22A*	42,251	7,092	42,462	6,447
hsa-miR-143-5p	48,540	4,847	48,782	8,431
hsa-miR-515-5p	45,634	5,757	45,863	6,195
hsa-miR-3150b-3p	44,506	4,452	44,732	7,336
hsa-miR-375	42,530	5,177	42,747	5,780
hsa-miR-769-3p	48,965	4,423	49,227	3,773
hsa-miR-376a-5p	46,805	5,832	47,058	4,901
hsa-miR-566	60,457	7,718	60,786	11,319
hsa-miR-3074-3p	45,994	5,625	46,247	6,721
hsa-miR-203	44,616	6,296	44,863	5,300
hsa-miR-944	45,338	4,731	45,589	4,896
hsv2-miR-H11*	47,024	5,860	47,286	8,074
hsa-miR-3139	40,633	5,710	40,861	4,961
hsa-miR-617	46,860	6,273	47,129	5,607
hsa-miR-3655	48,018	5,176	48,298	5,516
hsa-miR-1255b-5p	42,461	5,797	42,708	4,956
hsa-miR-4285	39,913	4,346	40,146	4,058
hsa-miR-384	44,156	4,834	44,415	6,028
ebv-miR-BART9	45,027	5,790	45,296	6,450
hsa-miR-668	46,503	5,332	46,784	4,185
hsa-miR-619	48,887	4,638	49,194	6,321
hsa-miR-127-3p	64,024	5,085	64,429	4,744
hsa-miR-3191-3p	40,432	5,862	40,688	5,950
hsa-miR-133a	53,137	5,611	53,477	6,947
hsa-miR-95	44,956	5,904	45,247	5,451
hsa-miR-3611	46,221	5,140	46,522	5,546
hsa-miR-934	43,921	5,369	44,208	5,353
hsa-miR-578	42,640	5,226	42,921	4,861
hsa-miR-553	46,402	4,997	46,710	5,335
hsa-miR-96-3p	40,688	3,976	40,959	3,948
hsa-miR-661	43,438	6,367	43,732	5,470
hsa-miR-145-3p	47,142	5,785	47,465	5,385
hsa-miR-491-3p	132,144	23,403	133,052	32,109
hsa-miR-4278	44,759	4,608	45,070	5,427
hsa-miR-802	42,324	6,272	42,619	4,873
ebv-miR-BART5	45,371	5,278	45,697	8,783
hsa-miR-3681-3p	74,592	10,060	75,130	26,635
hsa-miR-129-5p	53,606	5,359	53,995	7,000
hsa-miR-890	41,603	4,806	41,909	6,394
hsa-miR-185-3p	50,313	4,780	50,684	6,906

hsa-miR-412	45,280	3,296	45,617	6,025
hsa-miR-130b-5p	47,023	5,867	47,379	6,291
hsa-miR-3655	45,324	4,195	45,668	5,347
hsa-miR-559	44,984	4,763	45,325	5,162
hsa-miR-676-3p	43,160	4,311	43,498	4,140
hsa-miR-187-3p	44,532	5,672	44,882	3,509
hsa-miR-16-2-3p	52,004	4,373	52,414	4,586
hsa-miR-3155a	45,374	4,149	45,732	4,197
hsa-miR-374b-3p	47,825	5,151	48,203	7,333
hsa-miR-541-3p	40,264	4,049	40,582	4,043
hsa-miR-3687	42,633	5,705	42,971	5,714
hsa-miR-615-5p	42,338	6,331	42,674	4,288
hsa-miR-2110	44,538	4,058	44,895	5,691
hsa-miR-452-5p	42,270	4,612	42,610	5,309
hsa-miR-129-1-3p	69,163	5,928	69,722	11,869
hsa-miR-941	39,747	5,869	40,072	7,153
hsa-miR-29b-1-5p	65,276	4,753	65,815	6,397
hsa-miR-548l	44,632	6,603	45,005	6,288
hsa-miR-632	44,120	5,174	44,490	5,236
hcmv-miR-US5-2	40,970	4,858	41,317	5,941
hsa-miR-4264	43,792	5,104	44,163	4,635
ebv-miR-BART2-3p	40,901	5,320	41,251	6,073
hsa-miR-4272	40,957	4,017	41,307	5,622
hsa-miR-3611	40,874	5,634	41,226	5,278
hsa-miR-432-5p	62,391	6,456	62,931	6,182
ebv-miR-BART14	42,024	4,793	42,388	4,709
hsa-miR-556-5p	43,294	2,534	43,672	4,472
hsa-miR-579	44,338	5,636	44,729	6,125
hsa-miR-587	42,409	5,079	42,783	3,677
hsa-miR-486-3p	41,454	5,718	41,820	5,644
hsa-miR-450b-3p	51,024	4,996	51,475	4,902
hsa-miR-1289	45,621	5,544	46,025	5,107
hsa-miR-3613-3p	96,042	8,976	96,894	24,484
hsa-miR-3192	45,572	6,983	45,978	7,271
hsa-miR-642a-5p	43,411	5,763	43,798	5,933
hsa-miR-3916	47,315	6,212	47,738	5,251
hsa-miR-3186-5p	48,750	4,017	49,186	5,944
hsa-miR-769-3p	59,018	3,752	59,556	7,687
hsa-miR-1202	887,493	86,481	895,697	126,149
hsa-miR-518e-3p	46,207	4,183	46,640	4,383
hsa-miR-24-2-5p	39,156	3,516	39,526	5,423
hsa-miR-1293	41,795	4,718	42,189	6,070
hsa-miR-920	39,986	4,393	40,366	5,037
hsa-miR-611	43,944	5,339	44,363	4,787
kshv-miR-K12-7	45,347	5,497	45,785	6,949
hsa-miR-3618	45,358	6,737	45,797	5,865
hsa-miR-518e-3p	46,693	5,649	47,145	4,213

hsa-miR-520c-3p	47,273	4,675	47,733	4,716
hsa-miR-3924	50,833	7,117	51,333	5,806
hsa-miR-3922-3p	45,986	3,921	46,439	4,720
hsa-miR-219-2-3p	44,783	6,076	45,224	7,151
hsa-miR-1269a	40,545	4,415	40,947	5,552
hsa-miR-616-5p	42,362	5,166	42,784	5,403
hsa-miR-184	47,192	6,285	47,665	6,529
ebv-miR-BART20-3p	42,262	6,250	42,688	5,291
hsa-miR-148a-5p	66,639	4,181	67,322	16,097
hsa-miR-941	39,664	4,431	40,073	3,140
hsa-miR-302c-3p	46,974	5,207	47,461	3,958
hsa-miR-122-3p	47,187	5,960	47,682	5,094
hsa-miR-586	44,264	4,472	44,732	6,798
hsa-miR-604	47,319	4,822	47,821	5,483
hsa-miR-222-5p	43,709	3,791	44,175	4,640
hsa-miR-1255b-5p	40,980	5,036	41,423	5,099
hsa-miR-297	48,875	6,493	49,408	5,991
hsa-miR-3123	41,182	4,958	41,633	5,247
hsa-miR-302a-5p	46,067	5,135	46,572	6,744
hsa-miR-607	44,755	7,115	45,247	4,965
hsa-miR-3176	48,029	5,670	48,568	7,421
hsa-miR-135b-5p	51,465	5,577	52,052	5,637
hsa-miR-30a-3p	44,570	5,864	45,079	3,690
hsa-miR-4256	42,276	4,904	42,762	4,553
hsa-miR-3616-5p	42,873	4,458	43,369	4,740
hsa-miR-4329	49,878	5,235	50,456	7,651
hsa-miR-517a-3p	45,605	6,772	46,135	5,689
hsa-miR-1261	65,424	7,250	66,185	8,059
hsa-miR-4324	88,281	9,391	89,309	30,275
hsa-miR-668	46,392	6,390	46,934	5,276
hsa-miR-3684	46,816	4,876	47,365	6,684
hsa-miR-645	51,910	5,931	52,524	6,972
hsa-miR-612	45,787	5,997	46,330	4,973
hsa-miR-96-5p	50,350	5,996	50,949	5,695
hsa-miR-891a	47,740	5,282	48,313	5,068
kshv-miR-K12-2	42,355	4,474	42,868	4,221
hsa-miR-604	45,804	5,889	46,359	5,817
hsa-miR-3142	43,248	5,294	43,772	5,472
hsa-miR-3918	41,485	5,074	41,990	6,226
ebv-miR-BHRF1-2*	40,912	7,052	41,410	5,243
hsa-miR-873-5p	46,211	2,174	46,774	5,456
hsa-miR-4325	43,349	4,717	43,879	7,221
hsa-miR-576-5p	52,491	4,913	53,141	4,427
hsa-miR-889	46,238	3,124	46,811	3,141
hsa-miR-938	44,609	4,478	45,163	6,921
hsa-miR-934	44,261	4,134	44,813	5,533
ebv-miR-BART17-5p	40,975	2,908	41,493	3,887

hsa-miR-302a-5p	46,270	6,240	46,856	4,098
hsa-miR-3671	43,811	4,476	44,367	4,118
hsa-miR-216a	44,241	5,035	44,805	4,948
hsa-miR-96-3p	41,303	3,174	41,830	6,648
hsa-miR-141-5p	45,357	6,679	45,937	6,301
hsa-miR-1255a	42,887	5,904	43,437	3,754
hsa-miR-363-5p	41,676	5,533	42,216	4,898
hsa-miR-548b-3p	47,157	5,638	47,770	4,874
hsa-miR-629-5p	50,804	7,946	51,467	5,718
hsa-miR-3167	42,358	4,203	42,913	3,825
hsa-miR-195-5p	176,924	35,447	179,256	16,866
hsa-miR-1236	71,309	9,272	72,256	14,549
hsa-miR-3174	66,436	7,248	67,329	8,470
hsa-miR-556-3p	47,548	1,485	48,189	4,095
hsa-miR-18a-3p	50,146	4,692	50,825	5,623
hsa-miR-617	51,386	5,417	52,086	6,012
hsa-miR-518a-3p	45,940	4,911	46,570	5,651
hsa-miR-548x_v16.0	45,085	6,712	45,703	3,967
hsa-miR-550a-5p	65,476	5,661	66,374	6,723
hsa-miR-1273d	42,634	6,692	43,219	6,463
hsa-miR-25-5p	40,916	3,836	41,481	4,810
hsv2-miR-H7-5p	40,613	5,390	41,178	4,620
hsa-miR-200b-5p	41,863	5,634	42,446	5,341
hsa-miR-920	41,130	7,473	41,709	7,584
hsa-miR-662	60,611	5,127	61,463	7,699
hsa-miR-767-5p	43,085	6,815	43,697	5,604
hsa-miR-26a-2-3p	49,364	5,264	50,066	7,575
hsa-miR-3663-5p	45,296	4,337	45,941	5,532
hsa-miR-1243	47,764	5,701	48,444	7,077
hsa-miR-378c	52,494	10,777	53,246	6,192
kshv-miR-K12-12	44,160	5,707	44,798	6,273
hsa-miR-208a	40,392	3,959	40,980	4,707
hsa-miR-106a-3p	45,195	4,693	45,862	6,453
hsa-miR-363-5p	41,845	5,874	42,466	5,491
hsa-miR-1205	45,259	6,387	45,931	7,180
bkv-miR-B1-3p	49,510	6,833	50,250	2,543
hsa-miR-3664-5p	43,171	6,798	43,817	6,427
hsa-miR-1265	45,827	5,282	46,514	5,367
hsa-miR-302d-5p	41,540	6,202	42,165	6,006
hsa-miR-330-5p	46,239	9,240	46,936	5,109
ebv-miR-BART18-5p	46,526	4,512	47,229	5,638
hsa-miR-3118	44,381	5,279	45,057	4,551
hsa-miR-205-3p	122,426	11,272	124,302	31,156
hsa-miR-3914	40,752	4,120	41,380	5,311
hsa-miR-323b-3p	39,668	5,324	40,279	4,759
hsa-miR-518b	47,268	6,034	47,997	5,859
hsa-miR-4272	41,238	6,274	41,875	6,191

hsa-miR-4260	49,315	6,059	50,078	6,613
hsa-miR-302e	49,052	4,905	49,811	4,923
hsa-miR-3916	48,836	6,041	49,596	5,981
hsa-miR-586	48,604	6,046	49,363	7,755
hsv2-miR-H7-5p	39,828	6,043	40,452	5,809
hsa-miR-124-3p	46,586	2,709	47,316	7,001
hsa-miR-675-5p	40,381	4,001	41,022	6,360
hsa-miR-138-5p	45,626	3,983	46,353	5,556
hsa-miR-380-5p	41,892	3,828	42,564	4,678
hsa-miR-3140-3p	43,845	4,386	44,552	7,305
hsa-miR-1302	43,526	5,136	44,229	6,136
hsa-miR-199a-5p	52,260	6,610	53,104	6,270
hsa-miR-568	65,206	6,786	66,261	14,548
hsa-miR-3657	51,681	5,848	52,518	7,094
hsa-miR-125b-1-3p	43,951	5,976	44,665	5,558
hsa-miR-323b-5p	41,256	5,589	41,927	6,412
ebv-miR-BART4*	38,813	3,749	39,446	5,111
hsa-miR-3667-3p	44,697	5,140	45,426	4,868
hsa-miR-3912	44,251	5,907	44,976	5,994
hsa-miR-3688-3p	49,486	3,858	50,300	6,299
hsa-miR-3678-3p	41,536	5,526	42,221	5,400
hsa-miR-548s	43,132	5,795	43,846	6,343
hsa-miR-1912	39,983	4,499	40,646	5,773
hsv2-miR-H4-3p	44,851	4,818	45,596	5,019
hsa-miR-497-3p	44,049	4,656	44,781	7,637
hsa-miR-506-3p	46,549	6,382	47,328	6,282
hsa-miR-1273e	49,820	5,427	50,660	4,445
hsa-miR-1207-3p	54,038	7,306	54,952	6,747
hsa-miR-3146	42,482	6,165	43,201	7,508
hsa-miR-603	44,768	3,909	45,526	8,072
hsa-miR-433	43,315	6,034	44,051	5,650
hsv1-miR-H2	39,480	4,479	40,154	5,185
hsa-miR-1270	45,477	5,205	46,254	6,086
hsa-miR-23a-5p	47,531	3,474	48,346	8,232
hsa-miR-3657	52,882	5,473	53,792	7,175
hsa-miR-548u	46,895	5,590	47,702	5,090
hsa-miR-639	42,727	5,073	43,463	6,576
hsa-miR-208b	42,511	5,046	43,243	6,007
hsv2-miR-H7-3p	59,679	5,556	60,710	5,822
hsa-miR-657	43,754	3,565	44,511	4,943
hsa-miR-769-3p	50,287	6,421	51,157	7,964
hsa-miR-515-5p	43,204	4,393	43,953	5,720
hsa-miR-1298	40,562	4,198	41,272	5,121
hsa-miR-548c-3p	46,663	5,127	47,481	6,337
hsa-let-7c*_v16.0	44,288	4,920	45,064	4,814
hsa-miR-1909-3p	44,201	6,185	44,984	6,102
hsa-miR-525-3p	47,267	6,531	48,106	5,206

hsa-miR-485-5p	45,745	6,353	46,563	6,173
hsa-miR-577	43,458	5,674	44,236	6,005
hsa-miR-876-3p	42,567	4,366	43,331	6,913
hsa-miR-513a-3p	47,469	5,599	48,323	6,517
hsa-miR-30c-1-3p	47,600	5,301	48,460	3,767
hsa-let-7a-3p	50,671	5,818	51,594	9,657
hcmv-miR-US5-2	42,575	4,753	43,355	6,167
hsa-miR-518c-5p	44,846	4,594	45,673	5,454
hsa-miR-376b	41,839	4,880	42,614	3,683
hsa-miR-135a-5p	49,042	5,188	49,951	5,162
hsa-miR-3200-3p	45,151	5,005	45,990	5,791
hsa-miR-570-3p	45,330	6,106	46,172	6,391
hsa-miR-506-3p	44,922	6,174	45,758	4,735
hsa-miR-599	42,687	5,967	43,483	4,663
hsa-miR-637	46,754	6,079	47,627	5,749
hsa-miR-369-3p	45,249	6,501	46,095	5,500
hiv1-miR-TAR-5p	40,186	6,758	40,937	6,475
hsa-miR-216b	40,821	5,427	41,585	5,431
hsa-miR-517c-3p	45,835	4,961	46,693	5,568
hsa-miR-106a-3p	51,253	7,273	52,213	3,825
hsa-miR-3126-5p	43,524	5,088	44,344	4,451
hsa-miR-214-5p	46,948	4,318	47,833	6,285
hsa-miR-3681-5p	40,748	5,734	41,518	5,670
kshv-miR-K12-4-3p	39,496	4,277	40,243	6,354
hsa-miR-3686	39,738	4,416	40,491	5,552
hsa-miR-3130-3p	42,382	4,118	43,187	4,809
hsa-miR-3670	44,060	7,545	44,899	5,525
hsa-miR-3160-3p	45,757	7,214	46,629	6,177
hsa-miR-659-3p	47,689	6,550	48,606	5,083
hsa-miR-3669	41,378	5,347	42,174	4,396
kshv-miR-K12-7	43,670	6,481	44,510	6,835
hsa-miR-664-3p	116,822	12,124	119,081	7,734
hsa-miR-3163	48,759	4,557	49,704	8,549
hsa-miR-509-3-5p	39,432	3,645	40,197	3,386
hsv1-miR-H3	49,051	5,809	50,003	6,309
hsa-miR-3200-5p	41,706	4,590	42,518	4,148
hsv1-miR-H14-5p	42,355	4,926	43,181	5,445
hsa-miR-645	52,747	5,143	53,775	5,410
hsa-miR-3168	44,012	4,452	44,871	6,530
hsa-miR-191-5p	40,929	4,707	41,729	4,577
hsa-miR-3191-3p	42,458	3,950	43,288	6,033
hsa-miR-1273d	46,601	5,238	47,513	5,040
hsa-miR-493-5p	48,347	4,106	49,295	5,635
hsa-miR-1292	43,222	4,913	44,071	4,193
hsa-miR-302a-3p	52,026	4,994	53,048	5,606
kshv-miR-K12-6-3p	40,323	5,600	41,115	4,951
hsa-miR-3150a-3p	42,255	5,000	43,086	4,477

hsa-miR-2355-5p	43,430	6,106	44,292	6,553
hsa-miR-497-5p	49,898	6,100	50,890	5,128
hsa-miR-517b_v17.0	46,300	4,851	47,222	6,961
hsa-miR-194-3p	49,691	5,882	50,680	5,707
hsa-let-7f-2-3p	66,860	11,119	68,191	7,243
hsa-miR-492	45,296	5,359	46,198	2,985
hsa-miR-1273a	42,961	5,471	43,818	5,884
hsa-miR-933	56,243	6,527	57,365	5,323
hsa-miR-432-3p	58,642	6,012	59,813	8,155
hsa-miR-3675-3p	61,334	5,076	62,559	8,397
hsa-miR-617	48,249	5,796	49,214	6,304
hsa-miR-627	68,238	6,560	69,604	8,795
hsa-miR-520c-3p	47,930	4,106	48,893	6,394
hsa-miR-126-5p	48,399	5,686	49,371	6,841
hsa-miR-676-3p	42,159	4,682	43,018	5,546
hsa-miR-147a	45,392	4,894	46,317	5,548
hsv2-miR-H23*	44,179	4,871	45,082	4,050
hsa-miR-3680-3p	47,815	6,984	48,792	6,000
hsa-miR-4256	42,244	4,317	43,112	5,808
hsa-miR-922	40,971	4,751	41,815	3,973
hsa-miR-145-3p	46,074	5,795	47,025	6,219
hsa-miR-646	46,199	4,094	47,158	6,468
hsa-miR-135a-5p	48,018	4,335	49,017	4,886
hsa-miR-1283	40,739	3,929	41,588	5,975
hsa-miR-593-3p	49,410	6,546	50,441	3,925
hsa-miR-302e	48,305	5,550	49,315	7,365
hsa-miR-1265	47,612	4,820	48,609	4,311
hcmv-miR-UL22A	45,534	5,860	46,495	6,498
hsa-miR-606	43,051	4,349	43,962	5,000
hsa-miR-545-5p	47,947	3,033	48,962	6,007
hsa-miR-664-3p	98,511	8,877	100,600	8,741
hsa-miR-642a-5p	43,440	5,200	44,366	7,957
hsa-let-7a-3p	47,948	5,862	48,970	5,186
hsa-miR-491-5p	43,761	4,463	44,697	3,769
hsa-miR-519c-3p	45,237	5,311	46,212	5,014
hsa-miR-4307	54,207	4,879	55,375	4,804
hsa-miR-3713	53,622	3,531	54,781	6,998
hsa-miR-2114-5p	41,798	4,467	42,702	7,129
hsa-miR-4283	41,073	4,785	41,966	4,737
hsa-miR-491-5p	50,391	4,119	51,489	5,792
hsa-miR-649	43,474	3,927	44,430	5,433
hsa-miR-3927	43,273	6,037	44,237	5,235
hsa-miR-1266	44,692	5,827	45,691	5,927
hsa-miR-130a-5p	58,532	4,708	59,846	6,380
hsa-miR-218-5p	44,780	5,900	45,785	5,652
hsa-miR-3130-5p	46,716	6,797	47,773	7,116
hsa-miR-129-1-3p	53,755	6,928	54,972	5,475

hsa-miR-302d-3p	45,893	5,937	46,937	5,091
hsa-miR-924	51,057	4,736	52,220	5,770
hsa-miR-29c-5p	94,242	12,224	96,393	8,272
hsa-miR-551b-5p	44,427	6,019	45,441	9,239
hsa-miR-675-5p	39,549	6,082	40,452	4,788
hsa-miR-616-5p	46,524	6,770	47,590	4,984
hsa-miR-29a-5p	51,238	6,083	52,414	7,196
hsa-miR-3919	39,385	3,844	40,289	5,665
hsv2-miR-H3	50,999	6,045	52,170	6,327
hsv2-miR-H4-5p	40,369	3,767	41,297	6,573
hsa-miR-588	50,455	7,255	51,617	6,643
hsa-miR-1277-3p	46,071	5,250	47,134	5,330
hsa-miR-3167	42,933	6,069	43,925	6,158
hsa-miR-410	44,618	5,865	45,656	5,615
hsa-miR-1255a	41,467	3,778	42,433	5,428
hsa-miR-548k	42,972	4,038	43,977	5,644
hsa-miR-3929	47,566	6,296	48,684	7,735
hsa-miR-658	47,798	4,242	48,922	4,620
hsa-miR-548f	45,213	4,760	46,277	6,076
hcmv-miR-UL70-5p	42,332	5,760	43,333	6,911
hsa-miR-449c-5p	56,171	5,461	57,502	5,010
hsa-miR-3658	44,959	5,338	46,026	6,253
hsa-miR-217	41,295	3,683	42,277	5,661
hsa-miR-16-1-3p	49,428	5,313	50,609	5,062
hsa-miR-3616-5p	43,295	6,328	44,337	6,078
hsa-miR-4300	40,246	3,434	41,220	5,149
hsa-miR-3941	43,236	6,194	44,282	5,979
hsa-miR-553	46,660	6,738	47,792	5,468
kshv-miR-K12-11*	45,077	4,556	46,172	5,797
hsa-miR-590-3p	45,958	4,615	47,077	4,182
hsa-miR-2909	42,669	4,969	43,711	5,921
ebv-miR-BART10*	46,016	6,473	47,146	4,703
hsa-miR-143-3p	41,910	5,138	42,940	6,039
hsa-miR-155-3p	46,220	6,657	47,359	5,785
hsa-miR-1255a	42,202	5,014	43,242	4,720
hsa-miR-3942-5p	44,931	5,116	46,042	4,639
hsa-miR-658	42,644	5,882	43,700	2,751
hsa-miR-1468	43,310	4,742	44,387	6,074
hsa-miR-600	42,391	6,204	43,446	5,655
hsa-miR-4272	42,901	5,077	43,971	4,936
hcmv-miR-US25-1*	44,884	5,938	46,004	4,201
hsa-miR-3671	41,626	4,141	42,668	5,447
hsa-miR-1294	48,170	5,778	49,376	6,259
hsa-miR-30a-3p	46,335	5,640	47,499	4,979
hsa-miR-153	43,021	6,368	44,110	5,485
hsa-miR-4307	53,208	5,272	54,555	6,245
hsa-miR-3135a	39,984	4,771	40,996	3,449

hsa-miR-3661	40,617	5,449	41,647	3,783
hsa-miR-3130-5p	45,299	4,932	46,453	6,522
hsa-miR-3918	43,283	5,298	44,390	4,468
hsa-miR-1197	44,426	5,414	45,561	6,320
hsv2-miR-H4-3p	42,009	6,159	43,086	4,967
hsa-miR-576-5p	54,375	4,891	55,772	6,296
hsa-miR-3119	45,439	5,418	46,608	5,302
hsa-miR-616-5p	42,187	5,864	43,272	5,521
hsa-miR-4303	41,966	5,295	43,046	5,137
hsa-miR-367-5p	46,777	7,285	47,989	6,791
hsa-miR-3165	42,665	4,498	43,772	5,108
hsa-miR-2115-3p	43,352	6,843	44,478	4,184
hsa-miR-611	42,694	4,584	43,803	5,330
hsa-miR-3202	47,092	6,453	48,320	6,563
hsa-miR-873-5p	49,290	5,268	50,575	5,300
hsa-miR-95	48,597	5,267	49,868	5,294
hsa-miR-767-3p	49,930	5,777	51,246	4,789
hsa-miR-200a-5p	48,154	4,488	49,424	5,273
hsa-miR-558	43,216	4,581	44,356	4,921
hsa-miR-518f-3p	43,341	3,711	44,486	5,126
hsa-miR-377-5p	43,501	5,026	44,652	6,958
hsa-miR-27a-5p	43,856	5,609	45,027	5,996
hsa-miR-3664-5p	43,124	6,438	44,281	4,675
hsa-miR-608	43,883	6,491	45,061	5,262
hsa-miR-603	45,301	5,804	46,520	5,533
hsa-miR-302c-5p	43,784	5,628	44,964	4,621
hsa-miR-30c-2-3p	45,374	6,342	46,599	4,018
hsa-miR-4289	47,109	5,776	48,383	4,016
hsa-miR-218-1-3p	39,972	6,641	41,055	5,814
hsa-miR-601	70,281	6,714	72,186	9,870
hsa-miR-3187-3p	39,282	4,925	40,347	5,527
hsa-miR-302d-5p	40,175	3,279	41,266	4,451
hsa-miR-3186-3p	44,580	5,129	45,794	5,342
hsa-miR-196b-5p	62,605	6,123	64,312	8,681
hsa-miR-3934	56,295	4,587	57,836	8,145
hsa-miR-942	46,975	6,476	48,263	4,370
hsa-miR-1266	44,502	5,696	45,724	5,392
hsa-miR-519e-3p	48,225	5,085	49,550	6,484
hsa-miR-3688-3p	50,946	5,325	52,350	9,484
hsa-miR-4260	45,965	4,902	47,233	5,279
hsa-miR-99a-3p	47,323	5,171	48,631	5,179
hsa-miR-136-5p	59,177	7,383	60,814	7,588
hsa-miR-122-5p	53,598	5,609	55,094	6,890
hsa-miR-922	41,943	5,432	43,120	5,287
hsa-miR-187-3p	40,164	5,611	41,292	5,279
hsa-miR-154-3p	46,560	5,333	47,870	6,798
hsa-miR-376a-5p	45,081	4,350	46,353	5,464

hsa-miR-3614-3p	45,894	4,726	47,193	4,300
hsa-miR-3943	47,902	5,095	49,269	6,645
hsa-miR-1282	49,067	5,246	50,468	6,602
hsa-miR-3666	46,384	5,962	47,716	6,774
hsa-miR-301b	42,303	4,363	43,523	4,921
hsa-miR-553	44,256	6,301	45,536	4,926
hsa-miR-3184-5p	42,408	4,538	43,639	4,736
ebv-miR-BART10*	49,294	6,482	50,727	4,016
hsa-miR-924	49,242	6,163	50,675	5,133
hsa-miR-15a-3p	55,140	5,155	56,746	5,849
hsa-miR-604	41,864	6,390	43,089	4,421
hsa-miR-15b-5p	5622,573	429,592	5787,958	478,590
hsv2-miR-H19	42,734	4,980	43,991	6,523
hsa-miR-3936	42,464	4,618	43,714	6,086
hsa-miR-4275	44,453	5,100	45,764	6,312
hsa-miR-599	44,051	5,629	45,352	6,058
hsa-miR-2110	43,203	5,940	44,478	4,719
hsa-miR-544b	41,467	5,648	42,695	6,403
bkv-miR-B1-5p	44,793	4,405	46,119	5,000
hsa-miR-935	44,236	5,925	45,547	4,633
hsa-miR-649	44,248	3,845	45,567	5,339
hsa-miR-3916	47,256	4,508	48,665	2,835
hsa-miR-187-3p	42,302	6,732	43,564	6,769
hsa-miR-3137	70,824	8,685	72,953	12,047
hsa-miR-3159	43,494	5,268	44,803	5,577
ebv-miR-BART19-5p	39,881	5,107	41,084	6,301
hsa-miR-548m	49,536	5,803	51,034	5,842
kshv-miR-K12-2	42,072	5,382	43,346	4,219
hsa-miR-3689a-5p	46,117	4,345	47,515	5,249
ebv-miR-BART4*	40,589	6,074	41,820	5,733
hsa-miR-1285-3p	53,407	5,402	55,027	6,919
hsa-miR-526b-3p	45,927	4,984	47,321	6,752
hsa-miR-190a	46,050	5,373	47,451	5,644
hsa-miR-3666	46,060	5,700	47,463	6,358
bkv-miR-B1-3p	54,391	7,856	56,054	11,426
hsa-miR-561-3p	42,430	4,690	43,728	7,362
hsv1-miR-H2*	47,552	6,403	49,009	7,442
hsa-miR-3680-3p	45,095	5,742	46,479	5,993
hsa-miR-339-5p	38,138	3,166	39,309	4,367
hsa-miR-639	43,303	4,950	44,639	4,367
hsa-miR-31-3p	43,732	5,918	45,083	6,248
hsa-miR-3157-5p	45,902	4,963	47,321	6,568
hsa-miR-1324	44,277	5,668	45,649	5,750
hsa-miR-4287	46,325	5,219	47,760	7,848
hsa-miR-148b-5p	48,167	4,266	49,661	7,064
hsa-miR-2114-3p	44,863	4,518	46,263	5,566
hsa-miR-1206	43,913	5,704	45,284	4,839

hsa-miR-146b-3p	45,825	4,759	47,263	6,473
hsa-miR-2052	41,831	2,921	43,154	4,620
ebv-miR-BHRF1-2*	42,775	4,159	44,129	7,909
hsa-miR-29b-2-5p	55,825	4,673	57,592	5,538
hsa-miR-208a	41,391	4,379	42,703	6,463
hsa-miR-589-5p	44,711	5,321	46,136	6,505
hsa-miR-297	47,645	4,657	49,167	4,100
ebv-miR-BART9	41,076	3,926	42,394	4,095
hsa-miR-520d-3p	58,012	4,750	59,876	6,999
hsa-miR-1292	39,922	6,338	41,206	5,251
hsa-miR-624-3p	55,523	6,349	57,315	7,158
hsa-miR-497-3p	39,700	3,811	40,984	4,777
hsa-miR-3683	40,639	5,701	41,954	4,325
hsa-miR-655	42,905	4,742	44,297	6,243
hsa-miR-3619-5p	41,478	5,919	42,825	6,089
kshv-miR-K12-8	54,637	6,440	56,410	7,218
hsa-miR-589-5p	43,394	5,264	44,806	4,587
hsa-miR-34c-3p	38,597	5,891	39,853	4,441
hsa-miR-1304-5p	42,876	4,178	44,275	6,020
hsa-miR-3200-3p	48,308	6,288	49,884	5,462
hsa-miR-505-5p	53,147	6,813	54,882	4,211
hsa-miR-1205	45,475	4,305	46,961	8,107
hcmv-miR-US25-2-3p	42,177	3,635	43,559	5,613
ebv-miR-BART8	43,817	4,097	45,253	5,477
hsa-miR-4286	2906,745	136,354	3002,306	424,947
hsa-miR-376a-3p	42,788	4,148	44,196	4,511
hsa-miR-518c-3p	45,468	6,314	46,965	5,603
hsa-miR-890	39,654	3,491	40,962	5,065
hsa-miR-302b-3p	46,350	4,530	47,881	5,790
hsv1-miR-H14-3p	44,592	5,811	46,073	5,374
hsa-miR-711	46,165	5,923	47,699	6,086
hsa-miR-455-5p	46,370	5,145	47,918	5,653
hsa-miR-3143	42,106	5,437	43,513	5,535
hsa-miR-614	46,756	3,655	48,323	6,263
hsa-miR-377-5p	44,237	5,924	45,723	4,823
hsa-miR-3121-3p	46,245	5,535	47,804	6,079
hsa-miR-1294	49,482	4,026	51,152	5,780
hsa-miR-653	43,466	6,604	44,933	4,803
hsa-miR-548s	41,873	5,052	43,290	6,075
hsa-miR-616-3p	41,852	7,356	43,269	6,891
hcmv-miR-US33-5p	52,651	6,076	54,441	5,207
hsa-miR-644a	42,467	5,224	43,911	5,927
hsa-miR-4301	46,115	5,413	47,684	5,809
hsa-miR-920	39,799	3,251	41,153	6,358
hsa-miR-3673	43,074	3,628	44,541	6,068
hsa-miR-2277-3p	68,204	8,474	70,531	11,085
hsa-miR-505-5p	81,752	20,781	84,542	7,084

hsa-miR-3618	44,956	6,975	46,493	5,941
hsa-miR-3612	41,493	5,408	42,915	3,852
hsa-miR-508-3p	45,078	5,484	46,628	6,813
hsa-miR-612	45,758	6,222	47,334	5,117
hsa-miR-4321	43,153	5,088	44,639	5,230
hsa-miR-127-5p	40,863	4,226	42,271	5,376
hsa-miR-511	46,394	5,228	48,001	4,956
hsa-miR-325	46,677	5,293	48,295	5,742
hsa-miR-4287	43,968	5,302	45,494	5,352
hsa-miR-4301	43,739	5,667	45,260	5,148
hsa-miR-2052	39,886	4,685	41,274	4,768
hsa-miR-96-5p	47,497	5,385	49,151	6,391
hsa-miR-26a-1-3p	49,445	6,082	51,168	6,976
hsa-miR-3658	43,348	5,267	44,869	4,953
hsa-miR-655	46,887	5,934	48,534	5,713
hsv1-miR-H12	38,675	4,314	40,033	5,328
ebv-miR-BART2-3p	39,831	6,749	41,234	4,932
hsa-miR-519b-3p	46,020	4,685	47,644	7,044
hsa-miR-3165	41,953	4,481	43,434	6,110
hsa-miR-486-3p	41,541	4,961	43,009	5,420
hsa-miR-449b-5p	53,130	5,920	55,008	6,184
ebv-miR-BART14	46,043	5,079	47,678	7,744
hsa-miR-3074-3p	47,326	7,638	49,009	5,186
hsa-miR-497-3p	41,966	5,020	43,462	4,520
hsa-miR-548p	41,549	5,215	43,032	4,173
hsa-miR-573	39,602	4,531	41,017	4,474
hsa-miR-548g-3p	45,729	4,293	47,364	6,497
hsa-miR-875-5p	47,966	4,333	49,683	4,579
hsa-miR-551a	43,583	5,506	45,143	8,750
ebv-miR-BART22	43,910	4,635	45,484	5,597
hsa-miR-3145-3p	44,150	6,239	45,735	5,312
hsa-miR-143-3p	44,790	5,793	46,403	5,768
hsa-miR-891b	44,643	5,487	46,255	4,964
hsa-miR-639	40,220	6,438	41,672	4,983
hsa-miR-1468	44,511	3,851	46,122	5,024
hsa-miR-92a-1-5p	41,261	3,408	42,755	5,230
hsa-miR-27a-5p	41,826	4,822	43,343	6,387
ebv-miR-BART14*	43,724	4,203	45,313	3,690
hsa-miR-655	45,130	6,152	46,776	5,976
hsa-miR-518e-5p	42,648	5,119	44,204	4,060
hsa-miR-577	42,535	5,628	44,090	4,687
hsa-miR-592	49,921	5,121	51,752	7,506
hsa-miR-3149	281,524	31,700	291,851	22,099
hsa-miR-708-3p	41,471	6,095	43,002	5,257
hsa-miR-1262	44,505	4,473	46,151	4,092
hsa-miR-4300	43,519	4,917	45,131	6,626
hsa-miR-520a-5p	43,264	4,188	44,867	5,754

hsa-miR-675-5p	40,286	5,057	41,780	5,974
hsa-miR-649	40,663	3,785	42,174	4,790
hsa-miR-3660	47,386	6,174	49,148	5,015
hsa-miR-3691-5p	40,132	5,764	41,626	4,434
hsa-miR-411-5p	42,453	5,994	44,036	5,276
hsa-miR-3129-5p	45,863	5,799	47,578	5,509
hsa-miR-646	44,336	6,340	45,997	3,999
hsv1-miR-H5-5p	43,016	5,949	44,632	3,971
hsa-miR-4311	54,587	7,040	56,640	6,354
ebv-miR-BART2-5p	55,281	6,487	57,363	7,102
hsa-miR-585	44,068	4,938	45,732	6,292
hsa-miR-924	49,219	2,621	51,089	6,891
ebv-miR-BART3	43,741	5,108	45,406	5,713
hsa-miR-203	45,579	4,725	47,316	5,398
hsa-miR-3193	46,292	4,389	48,057	6,185
hsa-miR-579	46,550	5,864	48,327	6,384
hsa-miR-26a-2-3p	50,627	6,169	52,562	6,642
hsa-miR-944	46,542	4,877	48,324	3,916
hsa-miR-3606	45,627	6,731	47,377	7,048
hsa-miR-374a-3p	46,756	4,354	48,551	6,816
hsa-miR-508-5p	43,530	5,897	45,202	5,812
hsa-miR-323a-3p	42,621	6,099	44,259	5,755
hsa-miR-337-5p	42,837	5,721	44,484	6,156
hsa-miR-34c-3p	39,435	4,051	40,954	5,985
ebv-miR-BART15	49,288	6,995	51,188	8,438
ebv-miR-BART13*	41,764	5,051	43,376	5,877
kshv-miR-K12-11*	43,437	4,140	45,123	5,768
hsa-miR-185-3p	56,551	4,987	58,752	4,556
hsa-miR-367-5p	44,895	4,738	46,647	6,236
hsa-miR-586	46,605	5,475	48,429	5,337
hsa-miR-615-5p	40,011	7,287	41,579	2,881
hsa-miR-628-3p	47,640	5,389	49,509	7,664
hsa-miR-544b	42,521	4,216	44,193	5,257
ebv-miR-BHRF1-2*	40,659	5,637	42,261	4,278
hsa-miR-382-5p	52,540	7,218	54,617	5,029
hcmv-miR-UL36*	45,607	5,230	47,410	6,130
hsa-miR-1291	50,691	6,871	52,697	5,912
hsv1-miR-H11	39,507	4,796	41,070	5,352
hsa-miR-379-5p	41,032	4,551	42,658	7,254
hsa-miR-490-5p	48,246	4,218	50,167	4,952
hsa-miR-548b-3p	47,169	5,576	49,050	4,971
hsa-miR-135b-3p	39,211	5,883	40,775	5,439
hsa-miR-190b	50,820	5,829	52,846	5,605
hsa-miR-3611	42,377	6,507	44,069	5,551
hsa-miR-1252	42,024	4,709	43,705	6,211
ebv-miR-BART4	67,159	6,442	69,849	6,857
hsa-miR-377-3p	42,962	5,855	44,685	6,387

hsa-miR-3943	44,822	5,586	46,622	5,962
hsa-miR-3153	41,157	4,138	42,812	5,847
hsa-miR-3192	43,512	4,915	45,263	5,336
hsa-miR-450b-3p	51,819	5,168	53,905	7,548
hsa-miR-935	42,610	5,950	44,325	5,745
hsa-miR-548a-5p	57,401	8,282	59,712	8,948
hsa-miR-578	42,442	4,833	44,165	5,359
hsa-miR-3144-5p	40,771	5,146	42,431	5,307
hsa-miR-1279	49,855	5,860	51,887	6,229
hsa-miR-199a-3p	182,099	31,069	189,533	12,291
hsa-miR-593-5p	43,617	4,077	45,410	4,159
jcv-miR-J1-5p	42,250	5,804	43,990	4,192
hsa-miR-190b	53,178	3,668	55,372	7,845
hsa-miR-1471	73,751	14,726	76,803	18,699
hsa-miR-802	44,301	4,765	46,140	2,344
hsa-miR-219-1-3p	38,163	5,135	39,749	5,007
hsa-miR-493-5p	44,842	5,298	46,707	3,862
hsa-miR-1297	41,784	4,928	43,524	7,414
hsa-miR-382-5p	55,087	5,804	57,385	7,718
ebv-miR-BART7*	46,657	4,989	48,606	5,506
hsa-miR-183-5p	41,689	5,088	43,435	5,167
hsa-miR-377-5p	44,197	4,852	46,049	5,368
hsa-miR-1272	42,182	5,548	43,951	6,105
hsa-miR-599	43,389	5,897	45,210	6,062
hsa-miR-744-3p	91,430	10,435	95,272	33,796
hsv2-miR-H23*	42,494	5,509	44,279	5,730
hsa-miR-4296	44,006	5,937	45,857	4,908
hsa-miR-367-3p	45,502	4,442	47,417	4,876
hsa-miR-563	61,181	5,208	63,762	4,446
ebv-miR-BART14*	40,099	5,316	41,791	6,123
hsa-miR-367-3p	47,133	5,871	49,130	6,141
hsa-miR-520h	46,603	4,499	48,580	5,430
hsa-miR-491-3p	86,707	17,848	90,389	21,154
hsa-miR-656	48,683	4,918	50,755	5,905
hsa-miR-202-5p	46,160	3,789	48,129	4,754
hsa-miR-377-3p	43,905	5,465	45,783	5,856
hsa-miR-548a-5p	45,467	5,306	47,415	3,761
hsa-miR-3164	45,174	4,527	47,110	5,485
hsa-miR-616-3p	48,297	5,024	50,367	8,014
hsa-miR-3912	45,333	5,393	47,284	6,455
ebv-miR-BART8	44,526	5,911	46,443	4,415
kshv-miR-K12-8	52,610	6,459	54,881	6,079
hsa-miR-302b-3p	44,650	5,549	46,579	5,838
hsa-miR-639	39,399	5,158	41,103	4,826
hsa-miR-541-5p	54,968	3,839	57,347	10,571
hsa-miR-23b-5p	43,702	2,742	45,594	5,882
hsa-miR-1278	54,883	7,769	57,262	7,924

hsa-miR-181c-3p	59,823	9,903	62,422	6,815
hsa-miR-3713	50,220	7,221	52,404	8,639
hsa-miR-1185-5p	44,658	5,243	46,603	6,582
hsa-miR-3649	42,948	6,056	44,819	5,327
hsa-miR-589-5p	44,376	6,774	46,312	5,149
hsa-miR-1293	42,957	5,352	44,833	5,859
hsa-miR-3686	40,388	4,874	42,153	4,705
hsa-miR-3616-3p	57,079	7,476	59,581	8,183
kshv-miR-K12-9	37,418	5,388	39,067	4,092
hsa-miR-651	44,026	7,976	45,974	7,411
hsa-miR-3179	42,952	5,629	44,855	4,590
hsa-miR-3145-3p	46,715	4,812	48,786	4,393
hsa-miR-302b-5p	41,882	5,218	43,744	4,571
hsa-miR-1206	44,391	5,785	46,370	7,919
hsa-miR-548e	49,006	5,525	51,200	7,570
hsa-miR-1197	42,435	6,405	44,336	5,424
ebv-miR-BART6-3p	40,775	6,216	42,605	4,792
hsa-miR-100-3p	44,183	4,675	46,166	5,717
hsa-miR-606	40,638	5,652	42,468	5,562
hsa-miR-580	43,404	5,706	45,359	5,699
hsa-miR-576-5p	52,722	5,653	55,099	4,579
hsa-miR-621	48,653	5,923	50,849	8,708
hsa-miR-10b-5p	46,093	6,052	48,178	6,073
hsa-miR-615-5p	43,666	5,294	45,644	5,847
hsa-miR-329	44,479	5,341	46,497	4,326
kshv-miR-K12-2	45,985	4,587	48,076	6,155
hsa-miR-548b-5p	46,739	5,462	48,868	6,076
hbv-miR-B20	44,131	5,177	46,142	6,199
hsa-miR-411-5p	42,481	4,590	44,419	4,764
hsa-miR-3909	39,188	5,101	40,981	4,423
hsa-miR-2114-3p	43,094	5,557	45,071	5,590
hsa-miR-519c-3p	45,080	6,022	47,151	6,717
hsa-miR-2053	47,853	5,775	50,052	5,737
hsa-miR-490-5p	51,864	4,861	54,255	6,457
hsa-miR-1282	48,856	5,607	51,114	6,386
kshv-miR-K12-4-3p	40,821	5,687	42,710	7,828
hsa-miR-1207-3p	47,783	5,992	50,001	7,172
hsa-miR-644a	47,920	7,107	50,149	5,808
hsa-miR-653	44,151	6,843	46,204	6,526
hsa-miR-138-2-3p	67,093	6,710	70,214	9,624
hsa-miR-3116	40,944	4,424	42,850	5,643
hsa-miR-4304	58,496	6,147	61,223	5,682
hsa-miR-3921	47,543	5,924	49,762	5,907
hsa-miR-3654	62,296	4,031	65,210	7,534
hsa-miR-3680-5p	41,602	4,428	43,553	6,050
hsa-miR-633	42,874	2,207	44,887	7,014
hsa-miR-1228-5p	95,738	6,968	100,232	13,172

ebv-miR-BART18-3p	46,418	7,407	48,603	6,754
hsa-miR-512-5p	43,633	6,052	45,688	5,578
ebv-miR-BART11-5p	41,236	3,535	43,185	6,101
hsa-miR-761	47,462	5,126	49,709	7,682
hsa-miR-19b-1-5p	105,532	21,865	110,528	6,906
hsa-miR-122-5p	55,114	7,418	57,732	8,811
hsa-miR-216a	41,409	3,889	43,382	4,657
hsa-miR-374b-3p	73,105	18,232	76,594	5,863
hsa-miR-595	124,454	16,206	130,404	16,435
hsa-miR-183-3p	42,210	6,399	44,232	4,751
hcmv-miR-UL22A	48,681	6,963	51,017	3,253
hsa-miR-1179	52,207	5,800	54,713	5,519
ebv-miR-BART3	41,433	5,578	43,423	5,371
hsa-miR-708-5p	43,656	3,228	45,754	5,445
hsa-miR-190b	50,550	5,711	52,985	6,965
kshv-miR-K12-6-5p	45,794	4,731	48,000	5,513
hsa-miR-2355-3p	44,292	5,371	46,429	5,883
hsa-miR-642a-5p	48,653	6,397	51,002	5,364
hsa-miR-649	43,125	5,655	45,208	6,356
ebv-miR-BART21-5p	56,798	7,437	59,543	15,668
hsa-miR-661	43,963	6,690	46,101	4,841
hsa-miR-593-3p	51,275	5,731	53,776	6,194
hsa-miR-136-5p	58,076	6,028	60,911	7,353
hsa-miR-1250	43,300	6,567	45,416	6,619
hsa-miR-1301	44,014	4,325	46,168	4,872
hsa-miR-450b-5p	45,024	6,912	47,241	4,003
hsa-miR-567	43,518	3,180	45,666	5,300
hsa-miR-609	80,194	10,689	84,156	14,337
hbv-miR-B20	44,405	5,713	46,601	7,937
hsa-miR-200b-5p	47,548	6,750	49,904	5,854
hsa-miR-3941	42,598	5,271	44,722	6,320
hsa-miR-1827	41,872	4,985	43,964	4,581
hsa-miR-4280	50,482	5,406	53,017	6,177
hsa-miR-222-5p	47,709	5,620	50,110	5,668
hsa-miR-383	47,412	4,227	49,805	3,984
hsa-miR-3202	53,104	5,203	55,786	9,123
hsa-miR-487a	44,683	5,097	46,944	5,623
hsa-miR-302b-5p	45,332	4,811	47,627	5,244
hsa-miR-127-5p	41,355	7,083	43,451	5,315
ebv-miR-BART21-3p	43,039	4,006	45,220	5,840
hsa-miR-580	42,102	4,062	44,239	6,774
hsa-miR-223-5p	67,859	8,672	71,303	8,471
hsa-miR-1260a	1625,166	372,448	1707,762	403,060
hsa-miR-335-5p	45,197	5,085	47,495	5,799
hsa-miR-548u	43,446	5,564	45,657	6,046
hsa-miR-888-3p	42,710	4,490	44,885	6,909
hiv1-miR-TAR-5p	39,844	4,886	41,875	5,388

hsa-miR-3915	42,003	4,359	44,148	6,454
hsa-miR-607	43,318	5,564	45,534	5,237
hsa-miR-921	45,711	3,980	48,053	7,434
hsv2-miR-H3	52,500	6,375	55,195	6,403
hsa-miR-4303	41,728	6,189	43,873	5,467
hsa-miR-1291	42,871	5,362	45,076	5,294
hsa-miR-298	48,348	6,404	50,843	7,972
hsa-miR-126-5p	50,770	6,689	53,395	5,859
hsa-miR-548j	46,995	4,366	49,426	5,400
hsa-miR-3189-3p	61,914	6,073	65,119	8,048
hsa-miR-454-5p	93,245	13,598	98,087	23,136
hsa-miR-30c-1-3p	50,502	4,448	53,129	6,316
ebv-miR-BART4	61,647	6,227	64,862	7,796
hsa-miR-3692-3p	40,004	5,911	42,091	5,193
hsa-miR-558	44,113	4,257	46,415	5,279
ebv-miR-BART7*	43,441	6,671	45,711	6,269
hsa-miR-499a-3p	41,535	3,849	43,709	6,059
hsa-miR-3118	42,955	5,723	45,203	6,139
hsa-miR-323a-3p	43,056	4,837	45,310	5,119
hsa-miR-607	42,922	5,462	45,174	5,707
hsa-miR-1285-3p	73,226	6,356	77,076	8,097
hsa-miR-3200-5p	41,072	5,239	43,238	5,442
hsa-miR-4289	45,368	5,105	47,762	6,494
hsa-miR-3140-3p	40,722	4,651	42,874	4,616
hsa-miR-516a-5p	41,739	5,748	43,949	6,108
hsa-miR-105-3p	44,771	5,096	47,143	4,873
hsa-miR-603	44,728	4,524	47,100	6,344
hsa-miR-1245a	41,871	4,107	44,092	4,337
hsa-miR-4251	41,404	5,507	43,606	6,583
hcmv-miR-UL148D	54,393	4,799	57,289	5,033
hsa-miR-548j	44,667	4,598	47,046	4,985
hsa-miR-198	44,759	6,104	47,149	5,136
hsa-miR-570-3p	44,267	5,620	46,633	4,365
hsa-miR-520a-3p	48,459	4,737	51,055	5,317
hsa-miR-3154	57,186	6,219	60,252	7,287
hsa-miR-643	42,746	3,626	45,045	5,761
hsa-miR-299-3p	40,997	4,948	43,204	5,578
kshv-miR-K12-9	36,607	4,402	38,581	4,953
hsa-miR-32-3p	175,549	17,092	185,067	24,042
hsa-miR-508-3p	43,582	4,428	45,945	6,825
hsa-miR-507	43,813	4,434	46,191	5,909
hsa-miR-4325	43,213	4,554	45,562	4,458
hsa-miR-379-3p	44,706	5,324	47,150	4,375
hsa-miR-449b-5p	55,409	4,805	58,446	6,641
hsa-miR-942	44,103	4,862	46,520	4,329
hsa-miR-758	90,386	11,025	95,344	30,673
hsa-miR-518d-3p	46,813	4,902	49,385	5,219

hsa-miR-765	70,822	6,941	74,715	6,433
hsa-miR-422a	49,388	5,325	52,105	7,174
hsa-miR-591	43,393	5,279	45,781	3,183
hsa-miR-646	41,135	5,096	43,400	6,076
hsa-miR-1279	50,656	5,479	53,446	5,169
hsa-miR-3672	43,074	4,544	45,447	5,389
hsa-miR-578	43,768	6,134	46,180	4,778
hsa-miR-16-2-3p	57,614	6,482	60,795	4,583
hsa-miR-643	40,350	4,699	42,579	4,309
hsa-miR-543	49,237	5,410	51,958	8,143
hsa-miR-548p	42,005	6,977	44,327	5,672
hsa-miR-597	41,936	4,076	44,262	5,913
hsa-miR-1302	42,369	4,966	44,719	6,241
hsa-miR-876-3p	42,087	5,835	44,422	4,477
hsv1-miR-H4-3p	42,551	4,585	44,916	5,606
ebv-miR-BART9*	44,708	3,966	47,197	8,006
hsa-miR-548b-3p	47,549	6,486	50,197	5,951
hsa-miR-380-3p	47,711	5,483	50,369	5,041
hsa-miR-302f	49,385	4,316	52,140	6,577
hsa-miR-216b	40,131	5,370	42,372	4,990
hsa-miR-323a-5p	38,847	5,226	41,017	3,835
hsa-miR-3171	47,621	6,227	50,281	6,738
hsa-miR-496	42,962	5,444	45,364	5,829
hsa-miR-92b-5p	40,386	5,303	42,649	4,603
hsa-miR-29a-5p	46,624	6,486	49,237	6,595
hsa-miR-1304-5p	44,464	4,525	46,961	4,506
ebv-miR-BART6-5p	39,925	5,369	42,167	4,555
hsa-miR-202-5p	46,708	4,456	49,331	6,801
ebv-miR-BART3*	79,828	7,435	84,319	10,248
hsa-miR-548av-3p	44,592	5,116	47,117	7,072
hsa-miR-380-5p	40,784	6,186	43,097	5,197
hsa-miR-299-3p	43,308	4,957	45,766	6,624
hsa-miR-27a-5p	41,905	4,594	44,284	7,688
hsa-miR-26a-2-3p	49,703	3,912	52,527	8,578
hsa-miR-561-3p	40,943	4,341	43,272	3,200
hsa-miR-3919	39,863	5,018	42,137	4,719
hsa-miR-589-3p	41,772	4,217	44,164	5,552
hsa-miR-517b_v17.0	48,362	4,336	51,133	6,783
hiv1-miR-H1	140,991	26,607	149,079	33,375
hsa-miR-331-3p	896,431	201,073	947,939	190,791
hsa-miR-516b-5p	43,790	5,970	46,313	5,371
hsa-miR-920	39,166	3,868	41,425	4,689
hsa-miR-3149	296,304	30,841	313,400	29,096
hsa-miR-875-3p	42,434	6,949	44,888	4,923
hsa-miR-587	42,335	3,352	44,785	6,368
hsa-miR-1306-3p	113,626	15,888	120,214	16,334
hsa-miR-105-3p	44,179	4,946	46,743	2,926

ebv-miR-BART19-5p	39,192	5,551	41,467	5,303
ebv-miR-BART3*	52,544	5,917	55,605	7,762
hsa-miR-15b-5p	3373,494	281,712	3570,366	214,184
hsa-miR-450b-5p	48,747	3,108	51,597	8,284
hsa-miR-645	47,298	5,538	50,063	4,500
hsa-miR-581	45,488	7,365	48,152	6,583
hsa-miR-3922-3p	46,543	6,276	49,273	5,549
hsa-miR-495	43,059	5,049	45,602	4,843
hsa-miR-3115	45,123	6,834	47,789	8,062
hsa-miR-323a-5p	40,686	3,193	43,093	3,502
hsa-miR-323b-5p	41,889	5,658	44,374	6,080
ebv-miR-BART7*	42,594	4,530	45,123	5,035
hsa-miR-3613-5p	48,241	6,605	51,108	5,103
hsa-miR-3148	130,772	12,508	138,555	13,597
ebv-miR-BART22	43,874	5,620	46,486	5,023
hcmv-miR-UL36*	45,819	4,959	48,547	5,434
hsa-miR-2115-5p	45,010	5,998	47,695	5,277
hsa-miR-888-3p	42,621	4,143	45,165	4,051
hsa-miR-552	40,308	3,956	42,714	5,361
hsa-miR-3163	47,758	5,507	50,610	2,869
hsa-miR-186-3p	51,889	5,006	54,991	4,771
hsa-miR-135b-3p	39,342	6,380	41,696	5,322
hsa-miR-500b	57,830	5,122	61,313	5,460
hsa-miR-135b-3p	39,683	5,518	42,079	5,680
hsa-miR-767-5p	42,452	6,507	45,015	4,384
hsa-miR-221-5p	108,962	14,420	115,550	25,016
hsa-miR-372	43,548	4,644	46,188	5,997
hsa-miR-491-3p	119,436	19,021	126,677	34,973
hsa-miR-708-3p	43,452	4,618	46,087	6,290
hsa-miR-409-5p	43,184	4,932	45,816	4,722
hsa-miR-106a-3p	45,187	4,324	47,942	5,632
hiv1-miR-N367	47,638	4,066	50,544	6,825
hsa-miR-3129-5p	43,231	4,199	45,873	5,195
hsa-miR-124-3p	42,655	6,461	45,267	5,977
hsa-miR-491-5p	56,583	11,286	60,050	13,671
hsa-miR-450b-5p	43,838	5,185	46,524	4,489
hsa-miR-298	42,832	4,641	45,459	6,246
jcv-miR-J1-5p	43,104	4,203	45,749	7,122
hsa-miR-198	54,348	8,448	57,683	7,139
hsa-miR-219-1-3p	40,618	5,764	43,110	9,349
hsa-miR-545-5p	40,166	6,094	42,633	6,758
hsa-miR-3908	42,326	4,178	44,933	5,645
hsa-miR-1231	38,635	5,450	41,022	4,639
hsa-miR-145-5p	47,845	5,591	50,826	6,212
hsa-miR-141-5p	40,242	5,899	42,753	4,772
hsa-miR-586	43,540	5,899	46,260	5,473
hsa-miR-1260a	11798,972	622,193	12537,553	2353,802

hsa-miR-1323	49,158	5,518	52,237	6,333
hsa-miR-651	42,350	5,181	45,005	6,161
hsa-miR-544a	44,513	5,651	47,307	5,254
hsa-miR-1537	48,811	5,488	51,877	6,358
ebv-miR-BART9	41,409	4,733	44,016	4,134
hsa-miR-2278	56,639	5,792	60,215	7,449
hsa-miR-425-3p	76,052	7,325	80,867	6,679
hsa-miR-758	73,751	5,258	78,441	24,443
hsa-miR-1228-5p	105,874	7,560	112,609	11,578
hsa-miR-376b	39,855	5,037	42,392	4,852
hsa-miR-663b	43,897	4,429	46,694	5,214
hsa-miR-497-5p	42,673	5,446	45,398	3,935
hsa-miR-448	42,959	6,528	45,709	5,412
hsa-miR-525-5p	47,589	4,888	50,656	7,562
hsa-miR-720	76881,903	8435,303	81837,046	7840,791
hsa-miR-3668	40,937	6,358	43,576	6,171
hsa-miR-302d-5p	41,104	6,175	43,759	6,944
hsa-miR-200c-3p	48,128	4,902	51,243	6,275
hsa-miR-3680-5p	40,556	5,772	43,182	3,467
kshv-miR-K12-3*	39,101	5,419	41,634	4,905
hsa-miR-30d-3p	45,466	3,227	48,417	5,283
hsa-miR-3130-3p	45,285	4,939	48,226	5,193
hsa-miR-1248	41,398	4,660	44,092	5,712
hsa-miR-25-3p	1983,498	215,207	2113,603	192,245
hsa-miR-136-3p	42,156	4,669	44,933	5,736
hsa-miR-4304	59,058	6,240	62,949	6,536
hsa-miR-19b-2-5p	42,983	3,358	45,836	5,940
hsv2-miR-H4-5p	42,011	4,831	44,802	5,622
hsa-miR-302d-3p	42,993	2,948	45,850	6,243
hsa-miR-32-3p	171,392	12,898	182,810	20,478
hsa-miR-1913	60,492	4,753	64,530	9,413
hsa-miR-545-5p	43,243	4,306	46,145	6,182
hsa-miR-23b-5p	45,779	5,451	48,861	5,861
hsa-miR-708-3p	42,695	6,303	45,569	5,085
hsa-miR-555	42,453	4,770	45,315	6,012
hsa-miR-552	43,544	4,364	46,483	6,274
hsa-miR-92a-1-5p	42,500	3,166	45,371	4,807
hsa-miR-589-3p	47,065	7,030	50,253	7,347
hsa-miR-129-2-3p	68,450	5,798	73,098	11,538
hsa-miR-516b-5p	42,991	4,470	45,921	5,674
hsa-miR-562	39,699	5,203	42,409	6,648
hsa-miR-4280	50,728	7,501	54,193	6,511
hsa-miR-409-5p	45,067	6,933	48,146	4,933
hsa-miR-876-3p	44,875	4,926	47,946	5,091
hsa-miR-148b-5p	51,072	4,728	54,582	6,179
hsa-miR-601	66,744	8,236	71,339	8,470
hsa-miR-633	43,059	6,185	46,033	5,417

hsa-miR-548j	44,364	6,056	47,434	3,325
hsa-miR-374b-3p	53,205	5,875	56,887	8,074
hsa-miR-516a-3p	43,268	4,652	46,266	5,258
hsa-miR-298	45,147	3,616	48,289	7,081
hsa-miR-551a	46,100	6,191	49,312	6,970
hsa-miR-138-1-3p	41,682	6,387	44,587	5,784
ebv-miR-BART10*	45,387	4,418	48,554	6,859
hsa-miR-323a-5p	38,451	4,114	41,135	2,885
hsa-miR-1469	67,448	10,811	72,156	17,139
hsa-miR-4267	46,162	5,849	49,389	6,788
hsa-miR-587	42,211	4,472	45,163	8,269
hsa-miR-627	50,183	3,960	53,699	6,784
hsa-miR-106b-3p	41,000	3,824	43,878	5,864
hsa-miR-581	40,770	4,774	43,637	7,118
hsa-miR-448	44,721	6,179	47,874	5,764
hsa-miR-525-5p	44,220	5,222	47,350	5,287
hsa-miR-182-3p	45,340	3,990	48,549	6,434
hsa-miR-130b-5p	45,841	4,188	49,090	7,395
hsa-miR-3925-5p	55,908	6,385	59,873	9,859
hsa-miR-323b-5p	47,638	6,985	51,028	5,608
hsa-miR-188-3p	46,246	6,486	49,538	4,588
hsa-miR-370	50,073	4,320	53,650	9,428
hsa-miR-411-3p	83,878	9,077	89,879	33,263
ebv-miR-BART15	46,163	5,853	49,466	6,412
hsa-miR-4302	43,182	5,128	46,276	7,465
hsa-miR-518c-3p	46,127	5,746	49,439	7,104
hsa-miR-520d-3p	61,295	6,879	65,698	10,591
hsa-miR-23a-5p	74,472	4,350	79,830	10,176
hsa-miR-3921	43,984	5,664	47,160	4,764
hsa-miR-4272	43,270	4,008	46,404	5,612
hsa-miR-216b	40,383	4,177	43,315	5,137
hsa-miR-1262	42,311	4,400	45,386	7,359
hsa-miR-130b-5p	51,094	6,991	54,811	5,046
hsa-miR-3116	41,100	5,483	44,099	5,877
hsa-miR-892a	42,924	5,712	46,073	7,490
hsa-miR-3681-5p	38,506	4,503	41,331	5,373
hsa-miR-593-5p	43,790	5,719	47,007	5,721
hsa-miR-431-3p	54,198	6,161	58,181	8,089
hsa-miR-4286	27371,573	2502,323	29383,784	1209,743
ebv-miR-BART4	82,714	7,227	88,800	11,181
hsa-miR-1909-3p	43,535	5,195	46,746	5,261
hcmv-miR-UL36	48,105	6,812	51,679	4,407
hsa-miR-23a-5p	55,561	7,347	59,694	9,054
hsa-miR-383	49,676	6,412	53,373	7,269
hcmv-miR-UL70-5p	37,893	4,155	40,715	5,191
hsa-miR-151a-5p	235,843	52,448	253,432	36,185
hsa-miR-3689a-3p	44,692	5,691	48,029	7,130

hsa-miR-595	115,196	13,461	123,797	16,643
hsa-miR-10a-3p	43,220	6,046	46,451	7,571
hsa-miR-105-3p	45,727	7,847	49,146	6,963
hsa-miR-431-5p	42,775	4,545	45,979	5,296
ebv-miR-BART13*	40,346	6,084	43,376	6,068
hsa-miR-1180	89,909	8,973	96,664	12,495
hsa-miR-409-5p	43,129	5,458	46,375	5,504
hcmv-miR-UL22A*	42,254	4,640	45,459	6,519
hsa-miR-300	91,801	9,778	98,775	7,595
hsa-miR-1179	53,147	4,639	57,187	5,084
hsa-miR-3174	76,522	8,078	82,346	7,537
hsa-miR-573	38,468	4,786	41,398	5,507
hsa-let-7f-2-3p	54,925	4,867	59,109	9,480
hsa-miR-514b-3p	70,393	17,783	75,767	12,048
hsa-miR-618	46,169	5,104	49,695	6,355
hsa-miR-138-2-3p	73,761	11,604	79,401	13,483
hsa-miR-3609	63,726	6,340	68,599	7,620
hsa-miR-26a-1-3p	48,936	4,460	52,685	8,073
hsa-miR-15b-3p	67,940	11,433	73,149	3,955
hsa-miR-511	42,818	5,439	46,104	5,721
hiv1-miR-N367	50,051	5,766	53,893	4,664
hsa-miR-629-5p	100,422	22,520	108,132	10,217
hcmv-miR-US5-1	37,289	5,184	40,155	5,296
hsa-miR-3691-5p	40,528	5,014	43,644	5,714
hsa-miR-367-5p	41,644	5,640	44,849	6,354
hsa-miR-1	42,116	5,279	45,358	5,253
hsa-miR-3622b-5p	49,202	6,137	52,993	5,246
hsa-miR-449b-5p	51,693	4,172	55,676	4,726
hsa-miR-548n	45,240	3,917	48,728	5,214
hsa-miR-452-5p	41,110	5,398	44,283	5,388
hsa-miR-627	64,962	8,469	69,985	8,780
hsa-miR-219-1-3p	38,100	3,536	41,061	3,870
hsa-miR-541-3p	41,743	5,092	44,993	5,566
hsa-miR-26a-1-3p	47,808	6,184	51,531	4,998
hsa-miR-339-5p	41,567	5,946	44,810	5,685
hsa-miR-1273c	62,657	4,587	67,546	11,839
ebv-miR-BART18-5p	45,503	5,378	49,057	5,835
hsa-miR-26b-3p	49,411	5,081	53,282	4,571
kshv-miR-K12-4-5p	44,892	6,993	48,409	2,430
hsa-miR-509-5p	46,422	5,192	50,064	6,789
hsa-miR-431-5p	44,289	5,372	47,772	6,576
hsa-miR-302f	47,314	5,585	51,040	4,619
hsa-miR-525-3p	43,620	6,034	47,061	8,005
hsa-miR-2115-3p	41,577	3,940	44,860	6,220
hsa-miR-222-5p	41,876	4,248	45,185	5,312
hsa-miR-4276	41,589	5,317	44,884	4,780
hsa-miR-300	75,087	6,793	81,069	12,352

hsa-miR-541-3p	39,722	3,859	42,902	5,087
ebv-miR-BART11-5p	44,159	5,138	47,701	6,810
hsa-miR-449c-5p	50,924	2,672	55,016	5,411
hsa-miR-182-3p	44,173	4,803	47,722	3,998
hsa-miR-580	39,808	4,521	43,010	4,115
hsa-miR-1471	74,679	13,567	80,694	19,611
hsa-miR-218-1-3p	41,370	6,632	44,709	4,906
hsa-miR-627	56,827	5,906	61,415	4,913
ebv-miR-BART14	39,927	4,878	43,153	6,466
hsa-miR-3622b-5p	52,431	6,176	56,671	7,252
hsa-miR-23a-5p	56,820	4,113	61,423	5,898
hsa-miR-550a-5p	65,978	5,776	71,350	8,284
hsa-miR-32-3p	182,924	12,938	197,861	15,172
hcmv-miR-US25-2-5p	44,837	6,633	48,504	6,566
hsa-miR-431-3p	55,287	6,638	59,816	8,950
hsa-miR-2355-3p	46,063	4,605	49,859	5,900
hsa-miR-3685	52,789	3,724	57,166	7,963
hsa-miR-30c-2-3p	48,031	6,001	52,015	6,372
hsa-miR-106b-3p	43,430	4,904	47,038	6,033
hsa-miR-3651	251,247	34,128	272,177	19,134
hcmv-miR-UL36	44,044	4,181	47,714	5,762
hsa-miR-592	50,205	5,845	54,390	5,923
hsa-miR-3164	44,027	5,020	47,703	7,181
hsa-miR-145-5p	53,679	3,602	58,182	6,775
hsa-miR-200c-3p	87,779	7,737	95,147	11,139
ebv-miR-BART9*	40,979	5,184	44,421	4,152
hsa-miR-10b-3p	48,254	5,638	52,319	7,532
hsa-miR-520h	43,415	5,636	47,074	4,365
hsa-miR-3647-3p_v17.0	64,870	5,879	70,339	10,174
hsa-miR-3659	72,348	7,898	78,452	6,555
hsa-miR-3197	45,341	5,890	49,167	6,252
hsa-miR-206	85,058	7,919	92,252	14,612
hsa-miR-148b-5p	55,644	2,531	60,366	3,887
hsa-miR-650	69,576	6,794	75,485	10,919
ebv-miR-BART16	131,561	10,670	142,734	15,497
hcmv-miR-UL36*	43,528	5,669	47,238	4,471
hsa-miR-300	82,170	7,008	89,175	11,980
hsa-miR-514b-3p	57,955	11,721	62,899	7,583
hsa-miR-7-2-3p	63,628	4,715	69,074	19,345
hsa-miR-539-5p	75,505	6,501	81,991	7,640
hsv2-miR-H13	38,522	5,502	41,835	5,271
hsa-miR-4316	43,793	4,363	47,561	5,616
hcmv-miR-US25-2-5p	49,132	6,363	53,363	7,453
hsa-miR-942	47,163	5,537	51,233	6,237
hsa-miR-380-5p	39,461	3,966	42,877	3,929
hsa-miR-1178	45,174	4,327	49,089	5,588
hsa-miR-1236	59,867	5,815	65,058	12,065

hsa-miR-935	42,237	4,323	45,903	6,169
hsa-miR-658	42,822	4,275	46,549	6,550
hsa-miR-126-3p	64,217	7,408	69,813	7,352
hsa-miR-199b-5p	235,663	39,388	256,239	16,823
hsa-miR-30b-3p	51,074	7,154	55,536	6,469
hsa-miR-2116-5p	47,069	5,133	51,187	7,645
hsa-miR-34c-3p	46,366	6,777	50,426	5,876
hsa-miR-448	44,241	6,388	48,118	5,196
hsa-miR-3143	39,704	3,886	43,196	3,848
hsa-miR-509-5p	48,941	5,902	53,252	6,507
hsa-miR-4324	89,537	10,595	97,440	32,849
hsa-miR-892a	44,339	3,172	48,257	4,905
ebv-miR-BHRF1-3	39,266	4,780	42,738	4,637
hsv1-miR-H8*	40,738	4,915	44,346	5,253
hsa-miR-3122	41,672	4,906	45,372	5,924
hsa-miR-3148	135,789	10,424	147,967	18,453
hsa-miR-99a-3p	50,094	4,659	54,596	6,547
hsa-miR-670	123,082	15,030	134,153	17,180
hsa-miR-543	49,300	6,701	53,735	10,243
hsa-miR-1	39,589	5,004	43,153	7,160
hsv2-miR-H9-5p	47,427	5,572	51,699	6,905
hsa-miR-4280	46,042	4,341	50,206	7,161
hsa-miR-208b	41,044	3,487	44,756	6,045
hsa-miR-1827	42,466	5,915	46,309	5,505
hsa-miR-125b-1-3p	41,041	4,855	44,757	5,013
hsa-miR-23b-5p	46,478	6,253	50,698	8,557
hsa-miR-648	42,743	3,916	46,647	5,337
hsa-miR-2116-5p	48,844	6,390	53,313	7,414
ebv-miR-BART14	43,285	4,925	47,258	6,077
hsa-miR-15a-5p	5812,290	337,959	6345,916	277,028
hsa-miR-3197	45,889	5,501	50,122	8,226
hsa-miR-499a-5p	57,381	6,771	62,736	6,744
hsa-miR-3647-3p_v17.0	61,679	7,443	67,442	8,448
hsa-miR-326	59,207	5,854	64,753	6,475
hsa-miR-637	44,607	7,291	48,789	5,414
hsa-miR-622	79,920	12,403	87,426	27,614
hsa-miR-20a-3p	56,828	4,946	62,183	7,412
hsa-miR-369-3p	45,106	5,588	49,369	6,996
hsa-miR-380-5p	40,700	5,814	44,548	3,849
hsa-miR-24-2-5p	42,204	5,536	46,194	5,997
hsa-miR-3189-3p	52,935	7,270	57,945	7,230
hsa-miR-93-3p	44,970	1,235	49,239	6,284
hcmv-miR-UL112	38,614	4,973	42,297	5,378
hsa-miR-330-5p	42,628	3,820	46,713	6,007
hsa-miR-96-3p	39,757	6,331	43,588	6,817
hsa-miR-330-3p	57,196	6,910	62,716	6,008
hsa-miR-570-3p	47,378	3,064	51,978	7,031

hsa-miR-590-3p	44,822	6,713	49,194	5,149
hsa-miR-758	81,449	6,331	89,396	31,482
hsa-miR-1321	44,434	5,763	48,776	5,691
hsa-miR-3671	39,812	5,982	43,728	5,737
hsa-miR-25-5p	41,931	4,553	46,068	4,535
hsa-miR-889	42,528	4,730	46,727	6,842
hsa-miR-3689a-3p	47,470	6,521	52,167	5,571
hsa-miR-100-3p	44,028	5,020	48,393	5,405
hsa-miR-939	345,588	33,927	380,041	41,463
hsa-miR-32-5p	43,865	6,439	48,246	8,751
hsa-miR-3647-3p_v17.0	66,619	6,148	73,275	9,791
hcmv-miR-UL70-5p	39,775	3,427	43,751	4,502
hsa-miR-3609	71,759	8,007	78,948	6,885
hsa-miR-628-5p	42,618	4,286	46,893	6,190
hsa-miR-647	94,694	11,580	104,198	25,406
hsa-miR-28-3p	39,037	5,822	42,972	5,793
hsa-miR-24-2-5p	42,746	2,562	47,057	4,357
hsa-miR-130b-3p	398,042	70,522	438,216	47,763
hsa-miR-3193	48,202	5,113	53,076	6,473
hsa-miR-24-3p	9859,955	559,515	10857,615	641,431
hsa-miR-1295a	47,571	5,926	52,414	7,544
hsa-miR-758	81,576	6,572	89,918	26,600
hsa-miR-3065-3p	44,585	5,089	49,177	7,417
ebv-miR-BHRF1-3	38,465	6,322	42,448	7,431
hsa-miR-27a-5p	41,761	6,679	46,092	8,907
hsa-miR-345-5p	57,962	8,411	63,982	7,250
hsa-miR-3616-3p	56,096	7,482	61,936	7,282
hsa-miR-3685	56,976	5,926	62,916	12,669
hsa-miR-448	43,778	5,043	48,371	6,378
kshv-miR-K12-9	35,094	4,241	38,798	3,866
hsa-miR-10a-3p	40,888	6,835	45,204	7,448
hsa-miR-331-5p	41,176	6,351	45,527	3,557
hsa-miR-3163	48,272	4,016	53,386	9,049
hsa-miR-331-3p	475,702	114,536	526,140	81,149
ebv-miR-BART8*	40,790	3,108	45,142	6,114
hsa-miR-670	112,274	7,797	124,293	17,349
hsa-miR-183-5p	40,451	1,116	44,809	5,596
hsa-miR-26a-5p	2952,281	844,853	3271,684	399,337
hsa-miR-595	143,168	16,573	158,690	24,724
hsa-miR-513a-3p	41,664	5,337	46,181	5,257
ebv-miR-BART15	48,530	3,936	53,796	6,427
hsa-miR-383	49,037	6,430	54,366	8,656
hsa-miR-548c-3p	46,814	6,612	51,934	6,993
hsa-miR-624-5p	72,572	10,913	80,550	8,420
ebv-miR-BART18-3p	40,482	3,679	44,938	4,269
hsa-miR-431-3p	55,475	4,015	61,598	8,213
hsa-miR-767-5p	42,357	4,488	47,035	4,703

hsa-miR-2278	97,673	8,226	108,519	12,070
hsa-miR-186-3p	48,386	4,026	53,796	5,024
hsa-miR-374a-3p	43,606	5,099	48,487	4,830
hsa-miR-626	40,651	5,392	45,203	7,677
hsa-miR-190a	47,835	4,928	53,201	5,311
hsa-miR-499a-5p	54,556	6,583	60,686	7,655
hsa-miR-1180	67,223	6,087	74,807	9,276
hsa-miR-1258	54,512	8,350	60,669	8,370
hsa-miR-3154	60,215	5,594	67,051	7,657
hsa-miR-622	78,515	13,345	87,441	28,680
hsa-miR-30b-3p	62,335	7,171	69,426	6,222
hsa-miR-508-5p	46,304	5,423	51,591	11,346
ebv-miR-BART12	205,216	32,038	228,920	36,241
hsa-miR-24-1-5p	43,973	5,996	49,054	5,427
hsa-miR-653	44,008	3,740	49,127	8,826
hsa-miR-320c	941,102	71,047	1050,906	52,606
hsa-miR-411-3p	83,197	6,408	92,913	31,248
hsa-miR-629-5p	44,766	7,744	49,998	3,079
hsa-miR-1258	58,219	12,177	65,032	10,847
hsa-miR-744-3p	77,383	9,294	86,444	27,318
hsa-miR-32-3p	188,711	15,194	210,877	13,565
hsa-miR-1913	57,579	6,153	64,371	6,115
hsa-miR-589-3p	43,986	5,113	49,217	6,441
hsa-miR-582-3p	48,919	6,602	54,767	6,236
hsa-miR-1275	392,153	190,600	439,062	148,597
hsa-miR-422a	50,020	7,126	56,032	9,706
hsa-miR-301b	97,980	25,499	109,807	5,577
hsa-miR-130b-5p	51,422	4,325	57,708	5,931
hsa-miR-573	38,342	4,981	43,074	4,594
hsa-miR-624-5p	50,705	4,655	57,032	4,555
hsa-miR-550a-5p	62,452	3,539	70,266	6,113
hsa-miR-3689b-3p	48,920	6,762	55,051	7,109
hcmv-miR-US25-1*	42,143	5,055	47,431	5,492
hsa-miR-302b-3p	42,378	3,568	47,700	6,041
hsa-miR-1244	45,547	5,129	51,330	5,435
hsa-miR-661	42,261	5,677	47,717	6,662
hsa-miR-199a-3p	100,485	17,062	113,460	7,173
hsa-miR-650	79,616	8,519	89,901	13,267
hsa-miR-576-3p	43,279	4,990	48,927	5,611
hsa-miR-300	89,534	9,308	101,226	11,095
hsa-miR-548k	44,361	4,599	50,175	6,951
hsa-miR-431-3p	50,927	5,603	57,605	9,586
hsa-miR-206	76,410	8,186	86,434	12,370
hsa-miR-539-5p	73,364	7,482	83,005	10,780
hsa-miR-30d-3p	49,034	6,426	55,500	3,509
hsa-miR-1271-5p	65,583	5,412	74,233	10,723
hsa-miR-4267	44,483	6,040	50,363	7,357

hsa-miR-15b-3p	45,976	4,728	52,056	5,184
hsa-miR-1273c	65,225	5,678	73,883	13,519
hsa-miR-186-3p	48,710	5,573	55,187	4,150
hsa-miR-338-5p	54,197	4,770	61,411	8,269
hsa-miR-138-2-3p	71,920	4,368	81,532	10,741
hsa-miR-223-3p	17014,042	922,742	19301,688	1100,084
hsa-miR-500b	58,011	5,365	65,840	8,514
hsa-miR-7-2-3p	64,254	5,986	72,944	21,588
hsa-miR-3138	72,209	11,803	81,999	16,967
hsa-miR-3689b-3p	53,548	5,591	60,817	7,496
hsa-miR-450b-5p	43,554	6,029	49,497	6,791
hsa-miR-298	43,278	6,691	49,220	7,922
hsa-miR-338-5p	63,561	5,787	72,317	8,696
hsa-miR-125b-2-3p	50,366	4,866	57,360	5,793
hsa-miR-411-3p	78,462	11,036	89,402	33,230
hsa-miR-22-5p	86,591	19,094	98,686	7,628
hsa-miR-509-3-5p	42,206	5,539	48,126	6,984
hsa-miR-1244	50,655	6,017	57,781	5,462
hsa-miR-3685	60,577	6,849	69,119	12,254
hsa-miR-15b-3p	50,749	6,641	57,929	5,342
hsa-miR-3654	55,768	4,458	63,675	8,677
hsa-miR-103a-2-5p	53,454	5,187	61,037	7,947
hsa-miR-576-3p	44,922	5,536	51,420	6,662
hsa-miR-3176	58,143	5,063	66,574	11,224
hsa-miR-424-3p	43,854	5,105	50,214	7,511
hsa-miR-126-3p	87,757	12,991	100,493	12,134
hsa-miR-138-2-3p	79,456	12,888	90,992	11,200
hsa-miR-770-5p	79,134	10,737	90,655	10,500
hsa-miR-539-5p	73,879	6,959	84,679	8,786
hsa-miR-3138	70,030	9,240	80,373	12,508
hsa-miR-7-1-3p	81,282	14,821	93,472	7,852
hsa-miR-624-5p	59,302	6,869	68,214	7,089
ebv-miR-BART12	214,355	25,429	246,934	29,938
hsa-miR-151a-3p	57,327	7,851	66,123	7,247
hcmv-miR-UL22A	46,369	6,851	53,520	5,824
hsa-miR-25-3p	857,831	155,084	990,737	96,757
hsa-miR-3934	57,578	5,102	66,513	9,039
hsa-miR-181a-2-3p	49,084	5,502	56,717	4,916
hsa-miR-374b-5p	1108,306	345,434	1281,293	84,518
hsa-miR-1306-3p	103,067	8,364	119,177	13,781
hsa-miR-1287	69,376	11,503	80,260	11,964
hsa-miR-374c-5p	59,911	9,148	69,315	5,638
hsa-miR-374b-5p	1136,668	363,844	1317,185	66,620
hsa-miR-3065-3p	46,460	6,389	53,846	7,817
hsa-miR-142-3p	42349,026	2236,741	49147,808	8120,115
hsa-miR-26a-1-3p	51,815	6,394	60,168	8,113
hsa-miR-1295a	48,162	6,835	56,013	7,075

ebv-miR-BART21-5p	53,233	3,667	61,925	15,174
hsa-miR-1287	71,156	11,470	82,776	13,278
hsa-miR-765	79,411	7,604	92,408	15,139
hsa-miR-501-5p	71,349	6,368	83,055	9,899
hsa-miR-885-3p	72,799	13,351	84,750	16,433
hsv2-miR-H9-5p	48,713	6,461	56,712	10,486
hsa-miR-564	84,270	15,423	98,125	7,364
hsa-miR-3945	57,535	7,148	67,083	12,444
hsa-miR-501-5p	72,245	6,819	84,238	10,247
hsa-miR-30d-3p	58,620	10,538	68,414	6,790
hsa-miR-564	84,952	13,089	99,150	8,745
hsa-miR-202-3p	77,616	13,872	90,598	21,780
hsa-miR-326	78,476	4,536	91,610	6,627
hsa-miR-30b-5p	4525,998	366,230	5285,328	387,644
hsa-miR-628-3p	47,157	3,541	55,126	7,001
hsa-miR-22-5p	245,070	79,045	286,696	31,304
hsa-miR-378b	58,120	5,989	67,996	11,827
hsa-miR-338-5p	50,304	4,181	58,955	9,187
hsa-miR-330-3p	67,011	9,841	78,617	7,331
hsa-miR-505-3p	237,136	81,968	278,330	14,589
hsa-miR-33b-5p	44,682	6,142	52,498	5,634
hsa-miR-339-3p	65,809	9,126	77,344	5,298
ebv-miR-BART21-5p	52,041	5,621	61,206	12,131
hsa-miR-3945	59,651	7,304	70,161	14,676
hsa-miR-20a-3p	90,112	14,180	106,040	11,216
hsa-miR-10a-3p	43,460	6,472	51,143	8,461
hsa-miR-27a-3p	14092,373	755,908	16589,691	1369,412
hsa-miR-4317	191,580	54,461	225,642	19,254
hsa-miR-301b	53,420	10,254	62,963	4,535
hsa-miR-106b-5p	3035,640	182,272	3580,308	192,986
hsa-miR-103a-2-5p	55,426	5,995	65,377	5,731
hsa-miR-7-1-3p	112,867	20,509	133,146	13,107
hsa-miR-885-3p	52,345	6,589	61,816	7,613
hsa-miR-93-3p	57,813	7,782	68,333	6,708
hsa-miR-10a-3p	40,309	4,528	47,664	5,320
hsa-miR-3659	69,622	7,559	82,372	11,633
hsa-miR-422a	50,199	4,720	59,396	7,963
hsa-miR-223-3p	39860,983	4709,507	47178,543	8546,289
hsa-miR-33b-5p	44,820	5,885	53,060	6,077
hsa-miR-491-3p	90,410	19,805	107,049	30,839
hsa-miR-628-3p	57,494	6,536	68,093	9,915
hsa-miR-3651	123,459	21,458	146,335	14,557
hsa-miR-422a	50,419	7,819	59,873	8,380
hsa-miR-188-5p	172,643	62,867	205,066	39,900
hsa-miR-454-3p	73,018	13,663	86,798	8,783
hsa-miR-26b-3p	59,591	4,318	70,855	5,676
hsa-miR-320e	785,455	70,367	936,679	81,262

hsa-miR-193b-3p	128,670	26,287	153,484	20,362
hsa-miR-1261	71,705	6,494	85,613	12,731
hsa-miR-191-5p	39,604	4,611	47,316	5,828
hsa-miR-320b	773,444	89,322	924,206	99,204
hsa-miR-370	55,407	7,730	66,220	16,980
hsa-miR-628-3p	53,292	8,210	63,766	6,755
hsa-miR-378c	65,973	16,721	79,046	14,986
hsa-miR-3692-5p	102,728	18,178	123,133	34,143
hsa-miR-622	75,367	10,249	90,362	24,772
hsa-miR-545-3p	53,301	3,517	64,052	5,593
hsa-miR-3659	68,566	8,343	82,517	10,645
hsa-miR-1908	44,342	5,435	53,419	7,569
hsa-miR-1275	93,008	16,239	112,229	20,271
hsa-miR-141-3p	115,733	19,627	139,668	20,367
hsa-miR-24-3p	7703,267	536,220	9310,208	662,063
hsa-miR-598	49,132	3,622	59,453	7,412
hsa-miR-576-3p	46,291	7,825	56,038	4,743
hsa-miR-3127-5p	163,290	40,027	197,710	50,962
hsa-miR-3907	125,684	18,415	152,293	18,947
hsa-miR-30c-1-3p	71,735	7,744	86,963	11,917
hsa-miR-454-3p	213,373	53,291	258,803	27,190
hsa-miR-7-2-3p	62,825	8,924	76,227	19,772
hsa-miR-519e-5p	54,671	9,312	66,392	10,334
hsa-miR-885-3p	62,469	12,540	75,884	13,909
hsa-miR-650	65,496	8,534	79,573	11,290
hsa-miR-519e-5p	53,021	8,176	64,418	10,369
hsa-miR-3907	181,413	25,217	220,625	37,217
hsa-miR-320d	905,282	82,803	1101,060	59,055
hsa-miR-7-2-3p	64,170	8,168	78,061	20,136
hsa-miR-106b-3p	48,720	6,803	59,523	8,475
ebv-miR-BART12	132,237	12,029	161,606	18,978
hsa-miR-10b-3p	47,504	5,542	58,076	9,505
ebv-miR-BART12	159,603	15,175	195,244	27,603
hsa-miR-151a-5p	113,284	24,270	138,680	15,472
hsa-miR-744-3p	103,611	16,948	127,201	43,585
hsa-miR-103a-2-5p	60,059	5,592	73,743	7,029
hsa-miR-141-3p	66,302	11,470	81,413	8,782
hsa-miR-411-3p	77,994	8,520	95,907	32,474
hsa-miR-15a-3p	65,933	6,332	81,194	7,421
hsa-miR-939	396,026	84,005	488,714	73,346
hsa-miR-3156-5p	104,401	18,190	129,078	30,943
ebv-miR-BART20-5p	66,305	14,193	82,102	29,438
hsa-miR-590-5p	502,760	130,245	622,869	40,300
hsa-miR-188-3p	49,884	6,649	61,824	4,880
hsa-miR-10b-5p	50,559	3,989	62,674	9,808
hsa-miR-571	52,023	6,914	64,629	18,600
hsa-miR-130b-3p	245,657	68,910	305,558	28,556

hsa-miR-423-3p	67,488	8,356	83,998	6,385
hsa-miR-320d	405,841	36,171	505,880	27,509
ebv-miR-BART20-5p	65,760	9,122	82,037	25,717
hsa-let-7g-5p	13106,741	1086,975	16362,258	1916,380
hsa-miR-4313	100,895	9,704	126,024	8,403
hsa-miR-3156-5p	110,324	17,678	138,012	32,371
hsa-miR-151a-3p	69,037	12,085	86,403	12,078
hsa-miR-335-5p	65,530	11,094	82,083	6,720
hsa-miR-3652	95,199	11,201	119,253	32,348
hsa-miR-628-5p	53,138	6,540	66,606	7,382
hsa-miR-564	75,652	10,247	94,969	6,593
ebv-miR-BART20-5p	75,902	13,485	95,316	32,582
hsa-miR-24-1-5p	46,518	6,638	58,429	5,511
hsa-miR-378b	67,650	7,036	85,126	18,737
hsa-miR-3692-5p	84,282	18,192	106,127	26,286
hsa-miR-1274b_v16.0	43416,726	7311,010	54714,397	7098,468
hsa-miR-4317	63,526	9,763	80,155	9,113
hsa-miR-571	51,578	7,298	65,270	20,270
ebv-miR-BART20-5p	70,089	16,426	88,718	32,326
hsa-miR-221-5p	73,418	11,586	92,953	18,961
hsa-miR-1273c	61,572	7,081	78,072	19,749
hsa-miR-140-3p	550,192	99,813	699,456	37,177
hsa-miR-4313	98,099	7,604	124,745	10,041
hsa-miR-374a-5p	2504,558	571,912	3185,673	171,769
hsa-miR-501-5p	65,282	6,468	83,324	8,433
hsa-miR-892b	82,811	10,464	105,716	20,808
hsa-miR-30d-3p	63,153	12,269	80,755	6,935
hsa-miR-1305	244,933	39,200	313,699	78,320
hsa-miR-628-5p	68,322	8,693	87,757	6,020
hsa-miR-770-5p	82,953	14,381	106,766	14,901
hsa-miR-744-3p	112,311	18,753	144,597	42,988
hsa-miR-421	53,636	6,020	69,210	5,618
hsa-miR-320e	268,684	32,372	346,742	11,436
hsa-miR-3198	268,897	56,709	347,109	82,356
hsa-miR-423-3p	74,508	14,188	96,265	6,066
hsa-miR-30e-5p	3197,144	552,334	4132,076	300,933
hsa-miR-516a-5p	45,248	5,357	58,488	8,540
hsa-miR-331-5p	49,981	7,549	64,923	10,166
hsa-miR-140-3p	1474,313	217,849	1916,370	148,430
hsa-miR-3127-5p	131,038	26,032	170,898	49,181
hsa-miR-374a-5p	2252,097	559,144	2939,392	177,922
hsa-miR-1271-5p	78,908	7,405	103,093	10,245
hsa-miR-30b-5p	2287,764	537,261	2992,370	292,191
hsa-miR-142-5p	4620,685	174,787	6059,780	512,301
hsa-miR-142-5p	4621,207	127,205	6064,332	528,037
hsa-miR-30a-5p	49,546	6,221	65,122	7,148
hsa-miR-202-3p	77,963	11,821	102,852	27,209

hsa-miR-892b	82,910	8,167	109,453	22,567
hsa-miR-374c-5p	174,106	62,621	230,328	16,524
hsa-miR-484	107,008	9,616	141,573	11,055
hsa-miR-770-5p	87,585	18,391	116,062	19,188
hsa-let-7a-3p	64,070	9,979	85,154	7,315
hsa-miR-665	55,960	3,472	74,508	14,560
hsa-miR-892b	84,354	7,467	112,340	22,481
hsa-miR-505-3p	100,418	23,140	133,801	9,552
hsa-miR-501-5p	74,380	5,739	99,219	11,851
hsa-let-7g-5p	8402,347	1044,328	11227,000	1306,687
hsa-miR-484	175,763	12,049	235,538	17,704
hsa-miR-106b-5p	1504,654	274,883	2017,680	91,924
hsa-miR-628-5p	67,712	8,887	90,856	10,156
hsa-miR-4324	102,458	7,777	137,546	29,433
hsa-miR-152	47,519	5,924	63,946	5,305
hsa-miR-135a-3p	96,541	16,642	130,111	32,152
hsa-miR-339-5p	65,099	17,570	88,152	7,545
hsa-miR-423-5p	424,305	119,056	575,071	69,041
hsa-miR-301b	59,505	14,343	80,697	11,162
hsa-miR-590-5p	343,764	98,314	466,391	26,929
hsa-miR-3607-5p	47,511	4,928	64,550	6,779
kshv-miR-K12-7*	58,767	6,539	79,902	13,464
hsa-miR-500a-5p	72,916	7,759	99,148	15,374
hsa-miR-100-5p	53,182	6,096	72,336	10,474
hsa-miR-4299	760,273	123,685	1036,176	87,832
hsa-miR-28-3p	48,209	6,153	65,707	9,371
hsa-miR-125b-2-3p	51,554	4,685	70,348	9,198
hsa-miR-32-5p	271,689	88,605	370,779	31,170
hsa-miR-770-5p	72,755	14,782	99,370	16,567
hsa-miR-142-3p	20138,151	971,853	27505,905	2855,597
hsa-miR-575	259,862	23,669	354,947	27,729
hsa-miR-483-5p	89,528	11,524	122,380	29,537
hsa-miR-32-5p	212,712	65,822	290,905	17,428
hsa-miR-1288	101,623	11,031	139,625	26,284
hsa-miR-3198	285,083	55,190	391,887	96,132
hsa-miR-1288	104,728	9,171	144,173	28,504
hsa-miR-320b	277,265	42,939	381,850	27,094
hsa-miR-4299	860,507	136,865	1185,342	70,662
hsa-miR-511	43,505	5,933	59,949	5,478
hsa-miR-1246	313,076	199,255	431,765	257,015
kshv-miR-K12-5*	57,588	7,769	79,507	16,192
hsa-miR-570-3p	52,244	7,122	72,144	11,200
hsa-miR-892b	78,161	10,348	108,017	21,366
hsa-miR-320a	212,653	37,945	294,009	27,175
hsa-miR-193b-5p	68,846	8,992	95,551	29,644
hsa-miR-320a	433,440	54,555	602,736	53,704
kshv-miR-K12-7*	51,685	7,795	71,899	15,661

hsa-miR-501-3p	48,851	8,471	68,049	6,158
hsa-miR-575	264,410	28,764	368,487	25,711
hsa-miR-1305	228,321	40,733	318,676	63,956
hsa-miR-489	45,875	6,144	64,141	8,527
hsa-miR-30b-5p	389,136	133,005	545,659	70,757
hsa-miR-338-5p	92,529	11,822	129,905	34,482
hsa-miR-30a-5p	79,069	19,914	111,084	8,627
hsa-miR-769-5p	58,825	6,879	82,671	7,046
hsa-miR-3607-3p	64,569	13,599	90,929	7,131
hsa-miR-212-3p	159,446	41,212	225,054	28,781
hsa-miR-339-3p	95,264	18,581	134,877	9,838
hsa-miR-598	59,649	8,205	84,485	9,405
hsa-miR-1301	52,592	8,029	74,506	6,184
hsa-miR-487b	118,839	31,292	168,649	35,437
hsa-miR-32-5p	123,735	33,958	175,629	9,404
hsa-miR-33b-5p	49,879	6,624	70,903	8,785
hsa-miR-545-3p	65,414	8,495	93,131	10,318
hsa-miR-4291	248,183	105,232	353,817	61,316
hsa-miR-19a-3p	1751,210	503,658	2502,158	199,421
ebv-miR-BART13	475,156	120,379	682,031	119,677
hsa-miR-26b-3p	66,899	10,156	96,181	9,002
hsa-miR-483-5p	109,267	16,025	157,160	53,277
hsa-miR-1537	94,575	25,037	136,065	10,046
hsa-miR-4291	375,897	147,719	541,199	98,397
hsv2-miR-H21	48,599	4,890	69,971	21,854
hsa-miR-423-5p	172,880	49,330	249,524	22,365
hsa-miR-3125	146,205	17,585	211,410	49,596
hsv2-miR-H21	46,312	6,991	67,084	21,342
hsa-miR-339-5p	73,378	21,158	106,508	9,691
hsa-miR-3125	124,418	13,400	181,155	43,084
hsa-miR-19a-3p	2323,764	357,406	3387,188	292,550
hsa-miR-1914-3p	189,133	26,284	276,226	57,234
hsa-miR-487b	203,931	76,536	298,250	68,195
hsa-miR-148b-3p	626,367	173,959	916,127	95,972
hsa-miR-19b-3p	6429,033	558,318	9412,257	1025,925
hsa-miR-3607-5p	48,224	5,555	70,645	9,820
hsa-miR-3652	75,783	8,480	111,022	33,792
hsa-miR-1274a_v16.0	4467,346	856,123	6552,085	1524,285
kshv-miR-K12-5*	56,193	6,186	82,591	17,239
hsa-miR-4306	771,659	163,302	1134,300	40,304
hsa-miR-582-3p	49,403	4,138	72,647	7,648
hsa-miR-1914-3p	172,195	20,478	254,198	52,584
hsa-miR-513c-5p	50,425	6,104	75,026	21,231
hsa-miR-500a-5p	76,460	11,467	113,927	18,828
hsa-miR-339-3p	121,653	26,663	181,399	15,167
hsa-miR-4306	1070,718	176,252	1605,106	157,656
hsa-miR-324-3p	709,788	108,167	1069,268	270,199

hsa-miR-15a-3p	84,508	12,041	127,885	7,497
hsa-miR-489	47,955	5,887	72,639	9,279
hsa-miR-301a-3p	343,996	115,931	522,061	30,402
hsa-miR-10a-5p	55,098	5,161	83,850	21,008
hsa-miR-92a-3p	742,228	58,122	1129,809	82,691
hsa-miR-1274a_v16.0	1109,791	372,229	1689,605	388,761
hsv1-miR-H15	193,483	56,975	295,946	51,651
hsa-miR-19b-3p	2832,521	610,337	4343,173	178,704
hsa-miR-1274b_v16.0	19177,434	3985,640	29553,600	5867,131
hsa-miR-20a-5p	3234,257	325,342	5004,912	677,195
hsa-miR-769-5p	72,818	11,523	112,724	9,381
hsa-miR-326	121,636	14,895	188,667	15,515
hsa-miR-345-5p	75,308	7,658	116,817	16,691
hsa-let-7f-5p	9247,233	1128,391	14347,369	799,561
hsa-miR-17-3p	70,281	17,543	109,092	9,068
hsa-miR-20b-5p	247,619	42,430	385,603	32,512
hsa-miR-132-5p	51,139	5,062	79,805	11,630
hsa-miR-92a-3p	333,870	35,475	521,288	47,254
hsa-miR-28-3p	64,565	8,046	100,827	9,948
hsa-miR-551b-3p	45,003	5,767	70,619	11,300
hsa-miR-30d-5p	444,902	68,705	700,113	39,126
hsa-miR-18b-5p	83,000	16,317	130,992	17,623
hsa-miR-3065-5p	45,434	6,211	71,881	12,996
hsa-miR-219-5p	67,348	12,782	107,014	7,638
hsa-miR-140-5p	884,103	180,949	1408,043	168,355
hsa-miR-582-5p	138,617	30,824	221,421	19,094
hsa-miR-502-5p	72,972	11,829	116,887	10,415
hsa-miR-513c-5p	48,856	6,221	78,513	25,873
hsa-let-7d-5p	3128,388	439,021	5037,226	542,394
hsa-miR-219-5p	60,317	9,919	97,184	4,970
hiv1-miR-TAR-3p	78,807	17,090	127,115	33,445
hsa-miR-545-3p	87,797	18,147	141,767	17,041
hsa-miR-582-5p	151,049	31,772	244,392	17,872
hsa-miR-424-3p	66,245	4,970	107,305	29,694
hsa-miR-513b	58,386	6,109	94,581	27,916
hsa-miR-30d-5p	334,928	67,968	542,906	27,084
hsa-miR-500a-5p	127,909	26,759	207,356	23,217
hsa-miR-744-5p	101,129	33,758	164,662	10,200
hsa-miR-148b-3p	133,212	33,586	217,099	11,031
hsa-miR-501-3p	58,770	8,598	96,207	6,945
hsa-miR-199b-5p	68,085	7,885	111,476	6,217
hsa-miR-324-3p	982,277	307,121	1611,175	441,802
hsa-miR-545-3p	100,344	19,465	164,983	18,345
hsa-miR-30c-5p	1359,498	464,813	2250,898	146,793
hsa-miR-27a-3p	5328,060	403,502	8862,822	1080,599
hsa-miR-664-5p	61,311	7,676	102,015	9,814
hsa-miR-30e-5p	829,878	292,226	1381,156	174,007

hsa-miR-140-5p	1008,542	192,229	1682,954	248,642
hsa-miR-10a-5p	68,737	6,015	115,175	32,113
hsa-miR-511	49,674	6,030	83,262	9,028
hsa-miR-188-3p	59,641	8,314	100,292	10,776
hsa-miR-191-5p	66,307	12,219	112,327	14,309
hiv1-miR-TAR-3p	76,080	21,646	128,984	29,319
hsa-miR-20b-5p	1142,910	201,482	1939,578	252,037
hsa-miR-98	290,410	118,712	492,996	43,279
hsa-miR-17-3p	190,067	70,771	322,814	20,421
hsa-miR-98	882,712	280,562	1501,365	123,004
hsa-let-7a-5p	13438,183	783,564	22877,451	3405,388
hsa-miR-3607-3p	94,608	23,936	162,275	16,803
hsa-miR-20a-5p	1893,191	435,297	3267,976	411,364
hsa-miR-340-5p	807,724	229,073	1395,147	84,883
hsa-miR-185-5p	934,622	220,735	1616,654	148,742
hsa-miR-93-5p	1445,094	389,021	2503,094	106,574
hsa-miR-513c-5p	57,269	4,600	99,759	30,658
hsa-miR-4261	97,101	27,871	169,528	30,899
hsa-miR-340-5p	802,222	221,610	1404,315	58,443
hsa-miR-513a-5p	141,395	11,555	247,931	61,718
hsa-miR-33a-5p	52,563	8,006	92,262	9,583
hsa-miR-425-5p	1512,892	449,650	2663,731	271,935
hsa-miR-744-5p	118,474	41,568	209,138	9,551
hsa-miR-502-3p	91,180	16,631	161,372	9,359
hsa-miR-4261	109,406	35,440	194,505	39,257
hsa-miR-513b	53,962	6,339	95,950	31,005
hsa-miR-128	252,385	89,247	450,531	24,742
hsa-miR-107	9558,550	595,935	17137,447	1254,220
hsa-miR-212-3p	161,895	43,727	291,405	35,495
hsa-let-7f-5p	18257,781	1756,960	32913,612	5121,217
hsa-miR-185-5p	558,733	163,015	1009,034	72,649
hsa-miR-191-5p	73,409	20,016	133,862	7,533
hsa-miR-3065-5p	45,959	2,640	83,850	18,449
hsa-miR-23a-3p	14183,498	2571,809	25931,952	2243,048
hsa-miR-18b-5p	225,704	61,474	412,727	29,009
hsa-miR-93-5p	386,384	122,051	711,883	104,008
hsa-miR-425-5p	718,559	214,547	1332,747	138,745
hsa-miR-30e-3p	205,439	68,477	382,703	22,544
hsa-miR-128	133,874	40,886	250,376	21,485
hsa-miR-1973	700,567	186,469	1340,249	86,411
hsa-miR-542-5p	59,081	8,076	113,143	19,295
hsa-miR-500a-3p	75,793	11,412	145,200	16,753
hsa-miR-30e-3p	106,263	29,442	203,718	14,800
hsa-miR-513b	63,373	7,568	122,785	38,164
hsa-miR-513c-5p	65,606	7,844	127,185	32,464
hsa-let-7a-5p	8075,387	796,126	15677,576	1085,689
hsa-let-7d-5p	1416,875	474,606	2760,048	185,310

hsa-miR-421	73,839	18,202	144,015	14,428
hsa-miR-542-5p	73,395	4,581	143,942	25,033
hsa-miR-30c-5p	294,544	108,131	578,327	52,859
hsa-miR-513a-5p	102,718	9,857	201,838	52,568
hsa-miR-3653	666,184	312,192	1309,803	455,805
hsa-let-7b-5p	4093,786	414,772	8083,778	924,134
hiv1-miR-H1	52,150	5,873	102,981	39,019
hsa-miR-148a-3p	180,736	69,002	358,112	35,523
hsa-miR-664-5p	89,615	9,064	178,607	23,327
hsa-miR-542-3p	55,987	3,711	111,742	13,695
hsa-miR-148a-3p	142,602	54,060	286,524	30,977
hsa-miR-502-3p	124,031	24,578	251,753	30,169
hsa-miR-27b-3p	733,678	238,363	1489,577	163,842
hsa-miR-1973	132,732	17,988	270,366	40,924
hsa-miR-107	2604,223	434,478	5311,757	664,245
hsa-miR-502-5p	97,257	14,919	201,706	24,739
hsa-miR-28-5p	249,405	48,298	518,002	12,635
hsa-miR-362-3p	259,522	99,779	544,819	20,442
hsa-miR-301a-3p	126,322	46,419	265,751	16,591
hsa-miR-500a-3p	150,536	26,515	320,870	36,707
hsa-miR-17-5p	1934,549	591,541	4127,924	319,070
hsa-miR-660-5p	388,582	126,855	830,321	70,165
hsa-miR-340-3p	86,426	19,956	186,229	19,864
hsa-miR-28-5p	467,429	74,684	1011,035	74,159
hsa-miR-3653	382,264	169,731	841,750	335,383
hsa-miR-103a-3p	8830,698	1715,730	19653,319	1902,714
hsa-miR-340-3p	158,513	45,028	353,132	39,286
hsa-miR-18a-5p	257,751	87,409	575,537	43,942
hsa-miR-501-3p	86,031	19,853	193,006	10,856
hsa-miR-513b	85,680	11,832	192,277	60,090
hsa-miR-501-3p	99,262	18,488	223,960	13,788
hsa-miR-18a-5p	152,211	46,792	344,685	25,146
hsa-miR-660-5p	949,206	306,189	2157,911	196,420
hsa-let-7b-5p	2751,616	336,829	6281,947	423,638
hsa-miR-17-5p	1092,753	367,926	2508,148	138,424
hsa-miR-221-3p	325,879	54,308	749,480	161,211
hsa-miR-23a-3p	4882,936	1212,005	11235,581	1136,148
hsa-miR-551b-3p	52,097	4,811	121,767	26,808
hsa-miR-23b-3p	491,533	187,340	1161,242	115,685
hsa-miR-362-3p	396,583	139,343	939,239	68,137
hsa-miR-132-5p	60,614	6,328	148,377	30,903
hsa-miR-17-5p	302,751	131,853	746,001	87,094
hsa-miR-221-3p	104,966	15,706	259,049	65,161
hsa-miR-152	92,890	15,588	230,724	15,378
hsa-miR-551b-3p	56,368	6,634	140,168	30,753
hsa-miR-342-5p	71,325	7,304	178,711	31,683
hsa-miR-532-3p	97,310	31,113	244,429	18,435

hsa-miR-324-5p	269,355	95,923	684,695	125,336
hsa-miR-324-5p	320,187	99,535	823,909	170,204
hsa-miR-4284	968,289	378,561	2502,622	256,033
hsa-miR-542-3p	68,972	7,630	178,713	31,123
hsa-miR-33a-5p	80,657	17,563	210,847	25,737
hsa-miR-103a-3p	4139,842	1093,670	11041,118	1253,022
hsa-miR-23b-3p	155,799	56,172	415,733	39,448
hsa-miR-542-3p	73,377	6,411	198,277	30,303
hsa-miR-27b-3p	234,185	74,140	643,127	61,328
hsa-miR-532-3p	207,565	80,099	583,139	32,112
hsa-miR-4284	9543,085	3849,699	26939,174	2403,725
hsa-miR-361-5p	413,307	108,871	1172,263	82,541
hsa-miR-450a-5p	63,480	12,111	184,061	15,062
hsa-miR-362-5p	280,315	120,717	852,856	93,235
hsa-miR-361-5p	169,114	56,702	516,194	16,406
hsa-miR-494	1844,572	240,750	5924,266	1553,868
hsa-miR-362-5p	135,586	53,888	439,527	30,789
hsa-let-7c	1379,585	369,073	4634,522	371,671
hsa-miR-450a-5p	74,040	13,656	254,622	19,060
hsa-miR-494	1020,973	35,245	3544,619	804,105
hsa-miR-378a-5p	60,967	11,896	216,938	33,822
hsa-miR-532-5p	177,067	76,374	650,953	37,799
hsa-miR-532-5p	305,197	127,503	1198,531	57,835
ebv-miR-BART19-3p	69,207	6,385	273,517	72,349
hsa-miR-342-5p	117,481	13,713	469,471	120,613
hsa-miR-378a-5p	64,659	14,741	261,314	37,936
hsa-miR-132-3p	120,496	11,864	517,920	91,841
hsa-miR-503	50,518	11,209	225,041	18,712
hsa-miR-132-3p	164,572	15,180	767,737	160,375
ebv-miR-BART19-3p	86,888	11,406	423,308	106,761
ebv-miR-BART19-3p	78,367	9,922	383,817	79,904
hsa-miR-424-5p	924,142	231,562	4723,832	256,194
ebv-miR-BART19-3p	89,848	12,931	471,322	103,896
hsa-miR-125b-5p	63,235	8,402	343,932	23,622
hsa-let-7c	288,669	108,176	1576,054	69,889
hsa-miR-503	57,203	13,996	344,471	31,311
hsa-miR-424-5p	437,858	123,765	2778,058	199,583
hsa-miR-100-5p	71,730	6,756	488,133	53,437
hsa-miR-503	63,140	19,124	453,342	47,637
hsa-miR-378_v17.0	118,585	51,782	985,173	109,198
hsa-miR-378_v17.0	208,926	117,351	1898,276	86,179
hsa-miR-342-3p	1927,565	760,896	18073,801	5029,573
hsa-miR-342-3p	862,867	428,323	9171,324	1979,378
hsa-miR-99a-5p	46,862	5,834	682,014	81,477
hsa-miR-125b-5p	91,169	10,994	1563,069	178,806
hsa-miR-99a-5p	59,243	5,951	1511,636	229,251
